# SRTsim: spatial pattern preserving simulations for spatially resolved transcriptomics

**DOI:** 10.1186/s13059-023-02879-z

**Published:** 2023-03-03

**Authors:** Jiaqiang Zhu, Lulu Shang, Xiang Zhou

**Affiliations:** 1 Department of Biostatistics, University of Michigan, Ann Arbor, MI 48109 USA; 2 Center for Statistical Genetics, University of Michigan, Ann Arbor, MI 48109 USA

## Abstract

Spatially resolved transcriptomics (SRT)-specific computational methods are often developed, tested, validated, and evaluated in silico using simulated data. Unfortunately, existing simulated SRT data are often poorly documented, hard to reproduce, or unrealistic. Single-cell simulators are not directly applicable for SRT simulation as they cannot incorporate spatial information. We present SRTsim, an SRT-specific simulator for scalable, reproducible, and realistic SRT simulations. SRTsim not only maintains various expression characteristics of SRT data but also preserves spatial patterns. We illustrate the benefits of SRTsim in benchmarking methods for spatial clustering, spatial expression pattern detection, and cell-cell communication identification.

**Supplementary Information:**

The online version contains supplementary material available at 10.1186/s13059-023-02879-z.

## Background

A wide variety of spatially resolved transcriptomics (SRT) techniques have been recently developed to enable gene expression profiling with spatial localization information on complex tissues. Some of these SRT techniques are based on single-molecule fluorescence in situ hybridization (smFISH) and examples include seqFISH [1], seqFISH+ [2], MERFISH [3], and split-FISH [4]. Some are based on in situ array capturing with examples including ST [5], 10x Visium [5, 6], Slide-seq [7, 8], DBiT-seq [9], and Seq-Scope [10]. Some are based on in situ sequencing with examples including FISSEQ [11], ISS [12], and STARmap [13], while some are based on microdissection with examples including LCM [14] and TIVA [15]. The collection of these SRT techniques has revolutionized many areas of biology [16–22] and has motivated the development of computational methods and bioinformatics tools [23] to enable a wide variety of SRT-specific analyses. In particular, the newly developed SRT methods allow us to identify genes with spatial expression patterns [24–28], perform deconvolution [29–34] and clustering [35–39] to characterize the spatial distribution of cell types, detect local structures and microenvironments on the tissue [40–42], and examine cell-cell communications [36, 37, 42–46] and gene-gene interactions [46–48] in a spatially informed fashion.

The development of SRT-specific computational methods and software tools requires the routine use of simulated/synthetic SRT data. Synthetic SRT data contains a known underlying truth, which enables objective evaluation of method performance. Synthetic SRT data also allows method evaluation to be carried out under a wide range of parameter settings, helping characterize the unique benefits and limitations of the developed method across different scenarios. In addition, synthetic SRT data allows the power and accuracy of specific analytical tasks to be evaluated under different SRT techniques, sequencing depths, and other experimental conditions, thus facilitating experimental design for maximizing the benefits of SRT experiments. Unfortunately, many existing simulations of SRT data in different methodology studies are often poorly documented, not reproducible, and/or fail to generate synthetic data that resemble real SRT datasets. In addition, simulations for assessing a particular method are often carried out under the same modeling assumptions underlying the method, which can lead to an overestimation of method performance. Therefore, it is important to develop an independent, reproducible, and realistic SRT simulation framework that can be used to facilitate the development of SRT methods for a wide variety of SRT-specific analyses. Such simulation frameworks have long been established in the parallel research field of single-cell RNA sequencing (scRNA-seq) studies, with multiple popular scRNA-seq simulators available for facilitating scRNA-seq methodology development and scRNA-seq analysis [49–55]. Unfortunately, while both SRT data and scRNA-seq data are in the similar form of over-dispersed counts [56, 57], SRT data contains additional spatial localization information that makes it challenging to directly make use of existing scRNA-seq simulators. Indeed, as we will show below, because scRNA-seq simulators cannot incorporate spatial information, they will fail to preserve the important spatial and structural patterns of the tissue transcriptome that are key features of SRT datasets.

Here, we develop the first SRT-specific simulator, which we call SRTsim (*S*patially *R*esolved *T*ranscriptomic *sim*ulator), for generating synthetic SRT data based on a wide variety of SRT techniques. SRTsim incorporates spatial localization information to simulate SRT expression count data in a reproducible and scalable fashion, thus facilitating SRT experimental design and methodology development. A key benefit of SRTsim is its ability to not only maintain various location-wise and gene-wise SRT count properties but also preserve the spatial expression patterns of the SRT data on the tissue, thus making it feasible to evaluate SRT method performance for various SRT-specific analytic tasks using the synthetic data. We characterize the properties of the synthetic data generated by SRTsim in a comprehensive way and illustrate the benefits of SRTsim for assessing the performance of spatial clustering methods, spatial expression analysis methods, and cell-cell communication identification methods.

## Results

### An overview of SRTsim

**Fig. 1:**
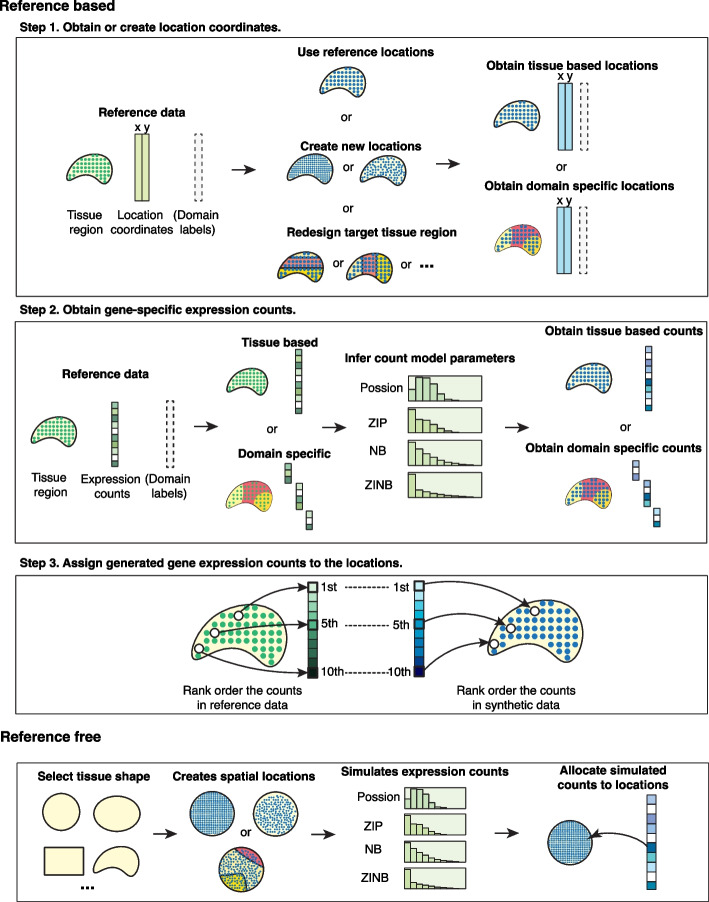
A schematic of SRTsim. SRTsim is a flexible SRT simulator that can perform either reference-based or reference-free simulations. Both types of simulations can be carried out in a tissue-based or a domain-specific fashion. In the reference-based simulations, SRTsim requires a reference SRT data in the form of a gene expression count matrix, a location matrix, and, for domain-specific simulations, an additional domain annotation matrix. SRTsim can directly use the reference data locations or create new locations and can redesign the target tissue region (Step 1). SRTsim then fits an appropriate count distribution to each gene in the reference and simulates the gene-specific counts in the synthetic data (Step 2). Finally, SRTsim assigns the simulated counts to the locations in the synthetic data in a way that preserves the spatial expression pattern observed in the reference data. In reference-free simulations, SRTsim allows users to design spatial patterns either from a customized shape or a predefined shape of interest and generate synthetic data with user-specified model parameters. P, Poisson; NB, negative binomial; ZIP, zero-inflated Poisson; ZINB, zero-inflated negative binomial

SRTsim is a general and flexible computational framework for simulating gene expression count data for spatial transcriptomics. SRTsim allows users to perform either reference-based simulations with a reference spatial transcriptomics dataset or reference-free simulations without such a reference. In the reference-based simulations, SRTsim first relies on the reference spatial transcriptomics data to either obtain the spatial coordinates of the measured locations or generate new spatial locations on the tissue as requested by the user. SRTsim then simulates the expression count data for one gene at a time using count models inferred from the reference data and allocates the simulated counts to spatial locations in the synthetic data in a way that preserves the spatial expression pattern of the gene observed in the reference (Fig. 1; see details in the “Methods” section). In the reference-free simulations, SRTsim allows the user to create a tissue section with an arbitrary shape, generate spatial locations on the tissue, simulate gene expression count data on each location based on a user-specified count model, and allocate the simulated expression counts to locations in the synthetic data based on user-designed domain-specific spatial expression patterns (Fig. 1 and Additional file 1: Fig. S1; see details in the “Methods” section). The key difference between reference-based and reference-free simulations is that the gene expression from the reference-based simulations either preserves or builds upon the spatial expression pattern observed in the reference while the gene expression from the reference-free simulations displays de novo spatial expression patterns defined by the user (regardless whether the tissue shape profile is obtained from an SRT data or created in a de novo fashion). SRTsim is implemented in an R package, which, along with an R-shiny app, is freely available at www.xzlab.org/software.html.

### Synthetic data generated by SRTsim resemble real SRT data

We benchmarked SRTsim along with eight existing single-cell expression simulators that include ZINB-WaVE [49], SPARSim [50], SymSim [51], two variants of scDesign2 (with/without copula) [52], and three variants of Splat in the Splatter package (Splat Simple, Splat, and Kersplat) [53]. To do so, we obtained 49 SRT datasets collected from eight different SRT platforms that include 10x Visium, MERFISH, seqFISH+, STARmap, DBiT-seq, ST, CosMx SMI, and SlideseqV2 (Additional file 1: Table S1). For each SRT dataset in turn, we applied all nine simulators to perform reference-based simulations. In the synthetic data, we calculated six metrics to characterize the critical properties of the simulated expression counts. The six metrics include four gene-wise metrics that characterize the expression properties for each gene across locations and two location-wise metrics that characterize the expression properties for each location across genes. The four gene-wise metrics include gene-wise mean, variance, coefficient of variation, and zero proportion, while the location-wise metrics include location-wise zero proportion and library size. With the calculated metrics, we compared each of them with that calculated in the reference data to evaluate how similar the synthetic data resembles the real data. Because the results are generally consistent across datasets, we will use the simulation results based on the 10x Visium data on the human dorsolateral prefrontal cortex (DLPFC) [17] and CosMx SMI data on the non-small-cell lung cancer (NSCLC) tissue [58] as the primary examples and list the simulation results for the other 47 datasets in the supplements.

**Fig. 2:**
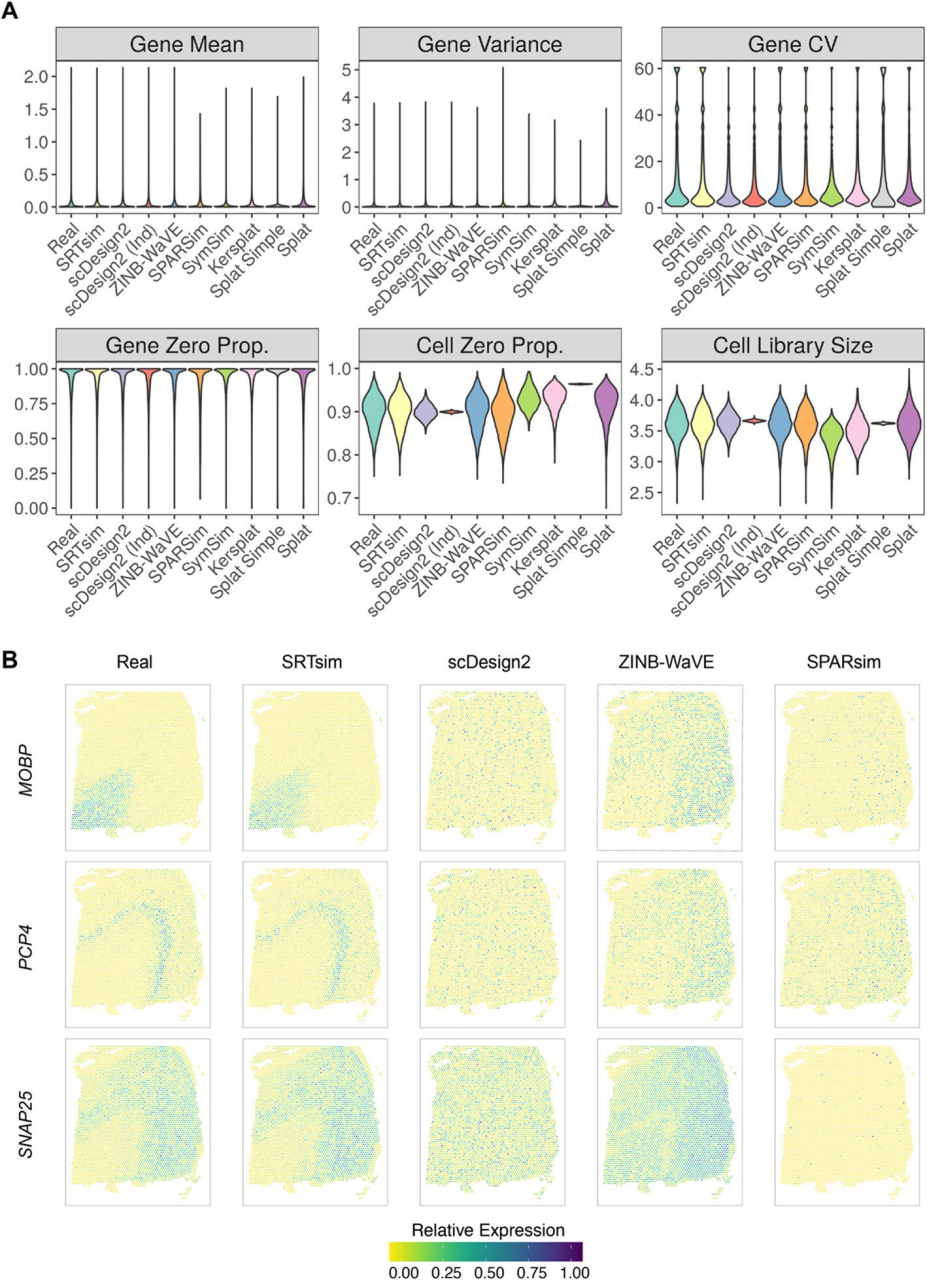
Benchmarking SRTsim against eight existing scRNAseq simulators for generating SRT data based on the DLPFC reference data. **A** Violin plots show the distributions of six metrics (six panels) in the reference data (real; *x*-axis) and in the synthetic data generated by different simulators (*x*-axis). The six metrics include four gene-wise metrics (expression mean; variance; coefficient of variation, cv; and zero proportion) and two location-wise metrics (zero proportion and library size). The examined simulators include SRTsim, scDesign2, scDesign2 without copula (scDesign2 (ind)), ZINB-WaVE, SPARSim, SymSim, and three variants of the splatter package (kersplat, splat simple, and splat). Results are shown for the reference sample DLPFC 151673. Results for the other reference samples are provided in the supplemental figures. **B** The spatial expression patterns of three representative genes (*MOBP*, *PCP4*, *SNAP25*) are displayed in the reference data and in the synthetic data generated by SRTsim, scDesign2, ZINB-WaVE, and SPARSim

**Fig. 3:**
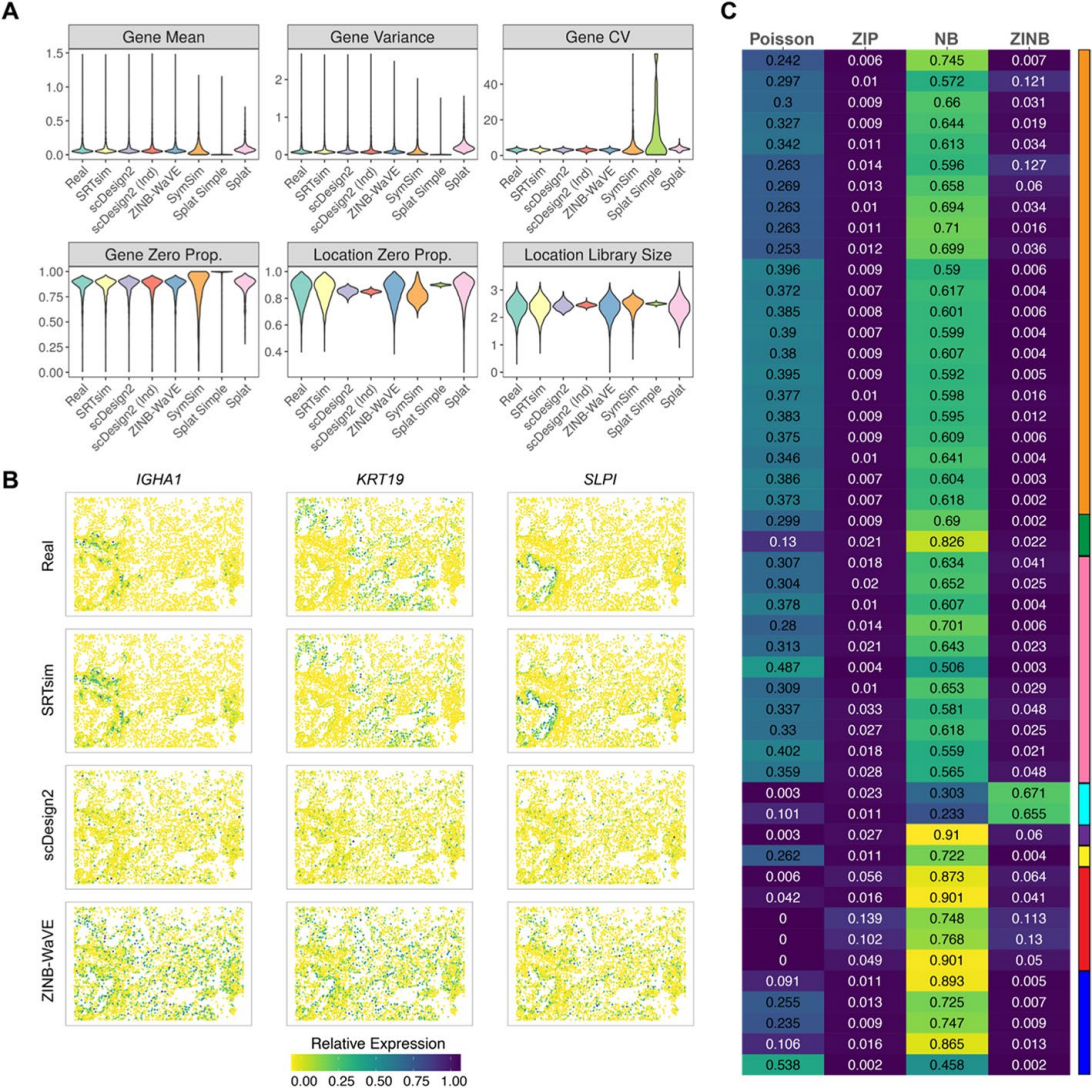
Benchmarking SRTsim against six existing scRNAseq simulators for generating SRT data based on the CosMx SMI reference data. **A** Violin plots show the distributions of six metrics (six panels) in the reference data (real; *x*-axis) and in the synthetic data generated by different simulators (*x*-axis). The six metrics include four gene-wise metrics (expression mean; variance; coefficient of variation, cv; and zero proportion) and two location-wise metrics (zero proportion and library size). The examined simulators include SRTsim, scDesign2, scDesign2 without copula (scDesign2 (ind)), ZINB-WaVE, SymSim, and three variants of the splatter package (splat simple, and splat). SPARsim and kersplat were unable to generate the synthetic data for this sample, therefore were excluded from the comparison. Results are shown for the reference sample Lung12 FOV1. Results for the other four reference samples are provided in the supplemental figures. **B** The spatial expression patterns of three representative genes (*IGHA1*, *KRT19*, *SLPI*) are displayed in the reference data and in the synthetic data generated by SRTsim, scDesign2, and ZINB-WaVE. **C** The heatmap shows the proportion of the preferred count model in each dataset. The right color bar represents the SRT techniques: 10X (orange), Spatial Transcriptomics (dark green), DBiT-seq(pink), seqFISH+ (cyan), STARmap (purple), SlideseqV2 (yellow), CosMx SMI (red), and MERFISH (blue). The corresponding dataset names are listed in Table S1. NB, negative binomial; ZIP, zero-inflated Poisson; ZINB, zero-inflated negative binomial

We compared the empirical distribution of each metric, either across genes for gene-wise metrics or across locations for location-wise metrics, between the synthetic data and the reference data for each simulator (Figs. 2A and 3A). The distributions of the six metrics suggest that only SRTsim and ZINB-WaVE are capable of producing synthetic data that preserve both gene-wise and location-wise properties in the reference. Both variants of scDesign2 can only preserve the gene-wise properties but not location-wise properties, while SPARSim is only able to preserve the location-wise properties but not gene-wise properties. In contrast, SymSim and three variants of Splat cannot preserve either gene-wise or location-wise properties (Figs. 2A and 3A). Quantifications with the Kolmogorov–Smirnov tests support the same conclusion. In particular, all metrics computed on the synthetic data by SRTsim are not statistically significantly different from those computed on the reference data (Additional file 2: Table S2 and Additional file 3: Table S3). However, almost all metrics on the synthetic data by the other simulators are substantially different from that of the reference data, with the only exception of the two location-wise metrics for ZINB-WaVE. The comparison results hold across different samples and different spatial transcriptomics techniques (Additional file 1: Figs. S2, S3, S4, and S5; Additional file 4: Table S4), except for the seqFISH+ data. For the seqFISH+ dataset, all the simulators, including SRTsim, fail to preserve the similarity for one gene-wise metric—the coefficient of variation (Additional file 4: Table S4), suggesting that certain data properties from seqFISH+ techniques may not be easy to capture fully. Indeed, consistent with [57], we found that while the negative binomial model is the preferred model for most datasets, the zero-inflated negative binomial model is the most preferred model for the seqFISH+ datasets (Fig. 3C), supporting distinct data features of seqFISH+.

A unique feature of SRTsim is its ability to preserve the spatial expression pattern of genes in the synthetic data, a feature not possessed by any single-cell expression simulators. To illustrate this benefit, we examined the spatial expression patterns of multiple layer-specific genes in DLPFC and compared them with the corresponding genes in the synthetic data generated by different simulators. Specifically, we examined three well-known marker genes [17]: *MOBP*, which is highly enriched in the white matter; *PCP4*, which is highly enriched in the L5; and *SNAP25*, which is enriched in neurons. We examined all simulators except for SymSim and the three variants of Splat, as these four methods preserve neither gene-wise nor location-wise properties (Fig. 2A). In the analysis, we found that only SRTsim can preserve the spatial expression pattern for all three marker genes in the synthetic data, while none of the other simulators can (Figs. 2B and 3B). In addition, we quantified the spatial expression pattern of all genes using Moran’s I and found that the Moran’s I statistics in the synthetic data generated by SRTsim are highly consistent with those calculated in the reference data, much more so than the other simulators (Additional file 1: Fig. S6), supporting the benefits of SRTsim in preserving spatial expression pattern for spatial transcriptomics simulations.

Another desirable feature of SRTsim is its ability to simulate data with a user-specified number of tissue locations, a feature that only scDesign2 and SymSim possess among the single-cell simulators. Simulating data with a user-specified number of tissue locations would allow us to determine the needed spatial resolution and/or sequencing depth for achieving certain analytical tasks in spatial transcriptomics, thus facilitating experimental design. To illustrate this feature, we applied SRTsim, along with scDesign2 and SymSim, to generate synthetic data on 1000 or 6000 spatial locations based on DLPFC. SRTsim and scDesign2, but not SymSim, preserve gene-wise metrics in the synthetic data regardless of the location number (Additional file 1: Fig. S7A). SRTsim also preserves location-wise metrics in the synthetic data, more so than scDesign2 and SymSim. In addition, SRTsim is the only method that can preserve the spatial expression patterns of the marker genes, though such patterns become less apparent with a lower number of spatial locations as one might expect (Additional file 1: Fig. S7).

Finally, we compared the computational resources required by different simulators on seven example datasets from different SRT platforms. We found that both SRTsim and the three variants of Splat achieve the best computational efficiency, while scDesign2 and ZINB-WaVE achieve the least (Additional file 1: Fig. S8). For example, for a SlideseqV2 data with 23,124 genes and 51,649 locations, it took SRTsim ~2 h to generate the synthetic data, while it took ~64 h for scDesign2 and 83 h for ZINB-WaVE. ZINB-WaVE also requires more than 250 gigabytes (GB) of physical RAM, while SRTsim only requires 50 GB. In terms of computational stability, SRTsim, scDesign2, ZINB-WaVE, Splat Simple, and Splat can generate synthetic data for all real datasets. In contrast, SPARSim failed to generate synthetic data for 20 out of the 49 datasets, and Kersplat failed for three datasets, while SymSim failed for one dataset (Additional file 1: Table S5).

Overall, SRTsim can generate synthetic datasets that closely resemble the real data for all eight SRT platforms in a computationally efficient fashion.

#### Application 1: Benchmarking spatial clustering methods using synthetic SRT data

We demonstrated the utility of SRTsim in benchmarking spatial transcriptomics methods for three analytical tasks. The first analytical task is spatial clustering for tissue domain detection, which aims to detect potentially functional domains or structures in the tissue using spatial transcriptomics information. Here, we focused on six spatial clustering methods for tissue domain detection that include five methods originally developed for spatial clustering (BayesSpace [59], stLearn with Kmeans, stLearn with Louvain [36], SpaGCN [39], HMRF [37, 38]), and one method originally developed for non-spatial clustering but previously used for spatial clustering (SNN in Seurat [35, 60]). For the benchmarking study, we performed reference-based simulations using two reference datasets: a non-single-cell resolution 10x Visium data on DLPFC and another single-cell resolution STARmap data on mouse visual cortex (details in the see the “Methods” section). For each data, we considered two types of simulations: tissue-based simulations where we directly simulated expression across all tissue locations, and domain-specific simulations where we simulated expression across locations within each tissue domain separately before combing them across the tissue. The domain-specific simulations create sharper contrasts among the spatial domains compared to the tissue-based simulations, thus representing a likely simpler scenario for domain detection.

**Fig. 4:**
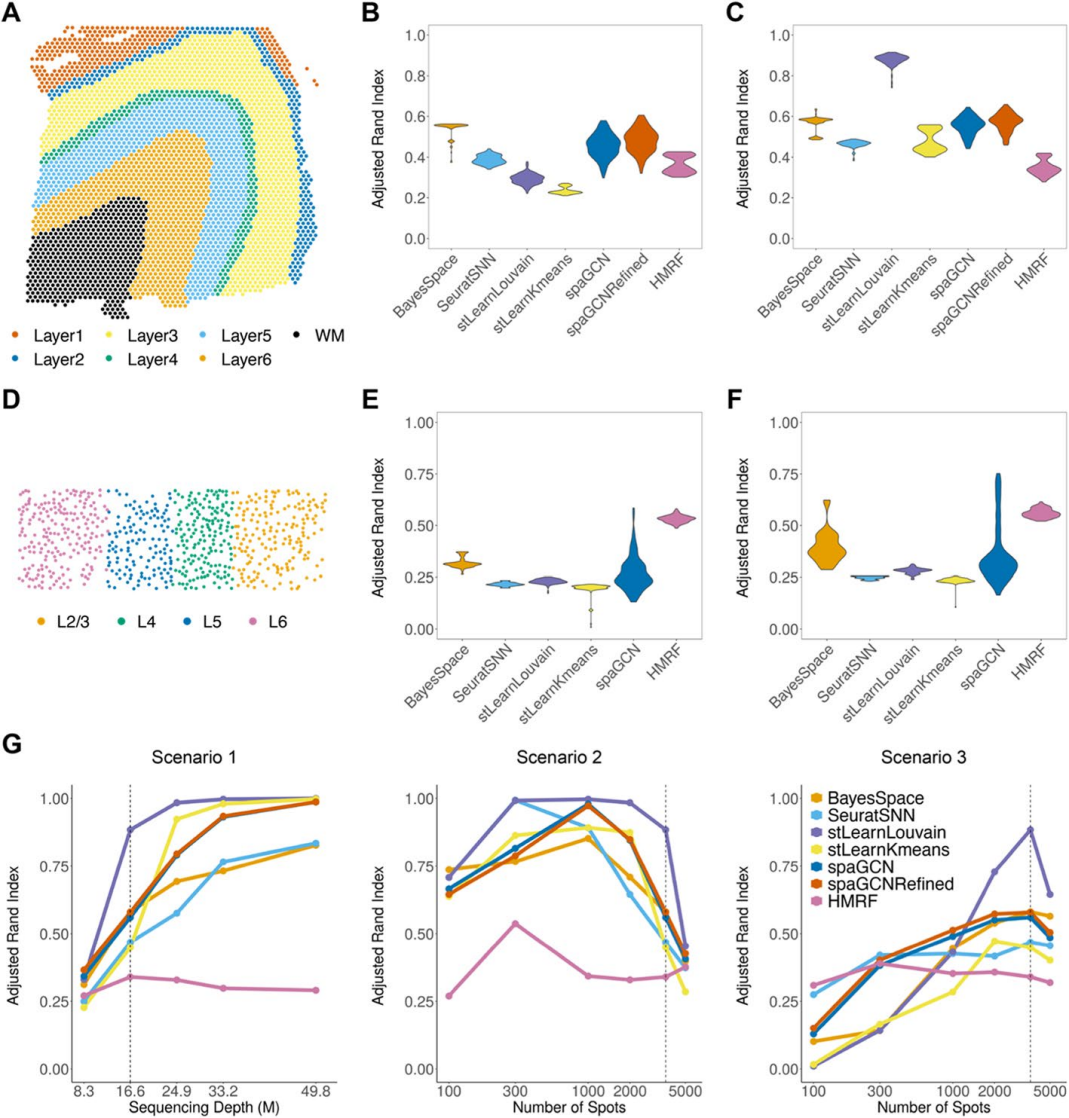
Benchmarking spatial clustering methods using synthetic data from SRTsim. The synthetic data are either generated based on the DLPFC reference data with seven original layers (**A–D**) or the STARmap data with four layers (**E–G**). **A** The reference DLPFC data contains seven layers, including six DLPFC layers and white matter (WM). Sample 151673 in the DLPFC data is used to serve as the reference. **B**, **C** The performances of six spatial clustering methods (*x*-axis) are evaluated based on the adjusted Rand index (*y-*axis) using synthetic data generated from SRTsim via either the tissue-based simulations (**B**) or the domain-specific simulations (**C**) based on the DLPFC reference. The six spatial clustering methods include BayesSpace, SNN, stLearn with Louvain, stLearn with Kmeans, spaGCN, and HMRF. **D** The reference STARmap data contains four cortical layers. **E**, **F** The performances of six spatial clustering methods (*x*-axis) are evaluated based on the adjusted Rand index (*y*-axis) using synthetic data generated from SRTsim via either the tissue-based simulations (**E**) or the domain-specific simulations (**F**) based on the STARmap reference. For **B**, **C** and **E**, **F**, ARI results are summarized across 100 simulation replicates. For the HMRF, we included the best result obtained by examining a range of values for the spatial parameter beta. For the SpaGCN, there is no refined version for the STARmap-based simulation, as no histology image is available for the data generated by this platform. **G** The performances of six spatial clustering methods in terms of ARI (*x*-axis) are evaluated under varying-sequencing-depth (scenario I), varying-location-number (scenario II), and fixing-average-sequencing-depth (scenario III)

We first examined the performance of different spatial clustering methods using the synthetic data generated based on DLPFC, which consisted of seven spatial domains, including six cortical layers and white matter (Fig. 4A). The synthetic data from both tissue-based and domain-specific simulations resemble the real data well (Additional file 1: Fig.S9). In the tissue-based simulations, BayesSpace achieves the highest ARI (mean across replicates = 0.54, median = 0.55, Fig. 4B) and identifies a clear layered structure that qualitatively follows the expected pattern (Additional file 1: Fig.S10). The performance of BayesSpace is followed by refined SpaGCN (mean = 0.48, median=0.49). In contrast, stLearn with Kmeans produces the lowest ARI (mean=0.24, median=0.23) with the inferred spatial clusters almost randomly distributed across tissue locations. In the domain-specific simulations, all methods, except for the HMRF, achieve higher ARI when compared to tissue-based simulations, as one would expect. Here, stLearn with Louvain achieves the highest ARI (mean=0.88, median=0.88) and accurately detects all layers (Additional file 1: Fig.S11). Its performance is followed by refined SpaGCN (mean=0.57, median=0.58) and BayesSpace (mean=0.56, median=0.58, Fig. 4C), both of which detect all seven layers but with layers 2–4 being wider than expected. The distinct clustering performances of these methods in tissue-based and domain-specific simulations suggest that spatial location information can help improve clustering accuracy, especially when the difference in gene expression across different domains is small.

We next examined the performance of different spatial clustering methods using the synthetic data generated based on STARmap, which contains four distinct cortical layers (Fig. 4D). Again, the synthetic data from both tissue-based and domain-specific simulations resemble the real data well (Additional file 1: Fig.S12). In the tissue-based simulations, HMRF achieves the highest ARI (mean = 0.53, median = 0.53, Fig. 4E) and identifies L5 and L6 layers but with mixed L2/3 and L4 layers (Additional file 1: Fig.S13). The performance of HMRF is followed by BayesSpace (mean = 0.32, median=0.31). In contrast, stLearn with Kmeans produces the lowest ARI (mean=0.19, median=0.20). In the domain-specific simulations, all methods again achieve higher ARI as compared to the tissue-based simulations. There, HMRF also reaches the highest ARI (mean=0.56, median=0.56), and its performance is followed by BayesSpace (mean=0.4, median=0.38, Fig. 4F and Additional file 1: Fig. S14). Note that HMRF performs poorly in the synthetic data generated from DLPFC but reasonably well in the synthetic data generated from STARmap data; we discuss the potential reasons in the “Discussion” section.

We performed additional simulations to examine the influence of three crucial experimental design parameters on the performance of spatial clustering. The three design parameters include the total sequencing depth, the average sequencing depth per location, and the number of measured tissue locations. We performed domain-specific simulations based on DLPFC for three scenarios: (I) varying the total sequencing depth while keeping the number of measured locations constant, (II) varying the number of measured locations while keeping the total sequencing depth constant, and (III) keeping the average sequencing depth per-location constant, while varying both the number of locations and the total sequencing depth (Fig. 4G). We found that the performance of all methods initially improves with increasing total sequencing depth in scenario I and reaches a saturation point beyond which further increase in total sequencing depth no longer substantially improves spatial clustering performance. Similarly, the performance of all methods initially improves with the increasing number of locations in both scenarios II and III. Afterwards, their performance reaches an optimal point in scenario II beyond which further increasing the number of locations reduces spatial clustering performance, and reaches a saturation point in scenario III beyond which further increasing the number of locations does not substantially influence spatial clustering performance. These results allow us to identify the optimal number of spatial locations and the optimal number of sequencing depths (Additional file 1: Fig. S15). For example, measuring 1000 tissue locations with a total sequencing depth of 16.6 million reads is reasonably optimal for spatial clustering analysis on a cortex tissue measured with 10x Visium technology.

**Fig. 5:**
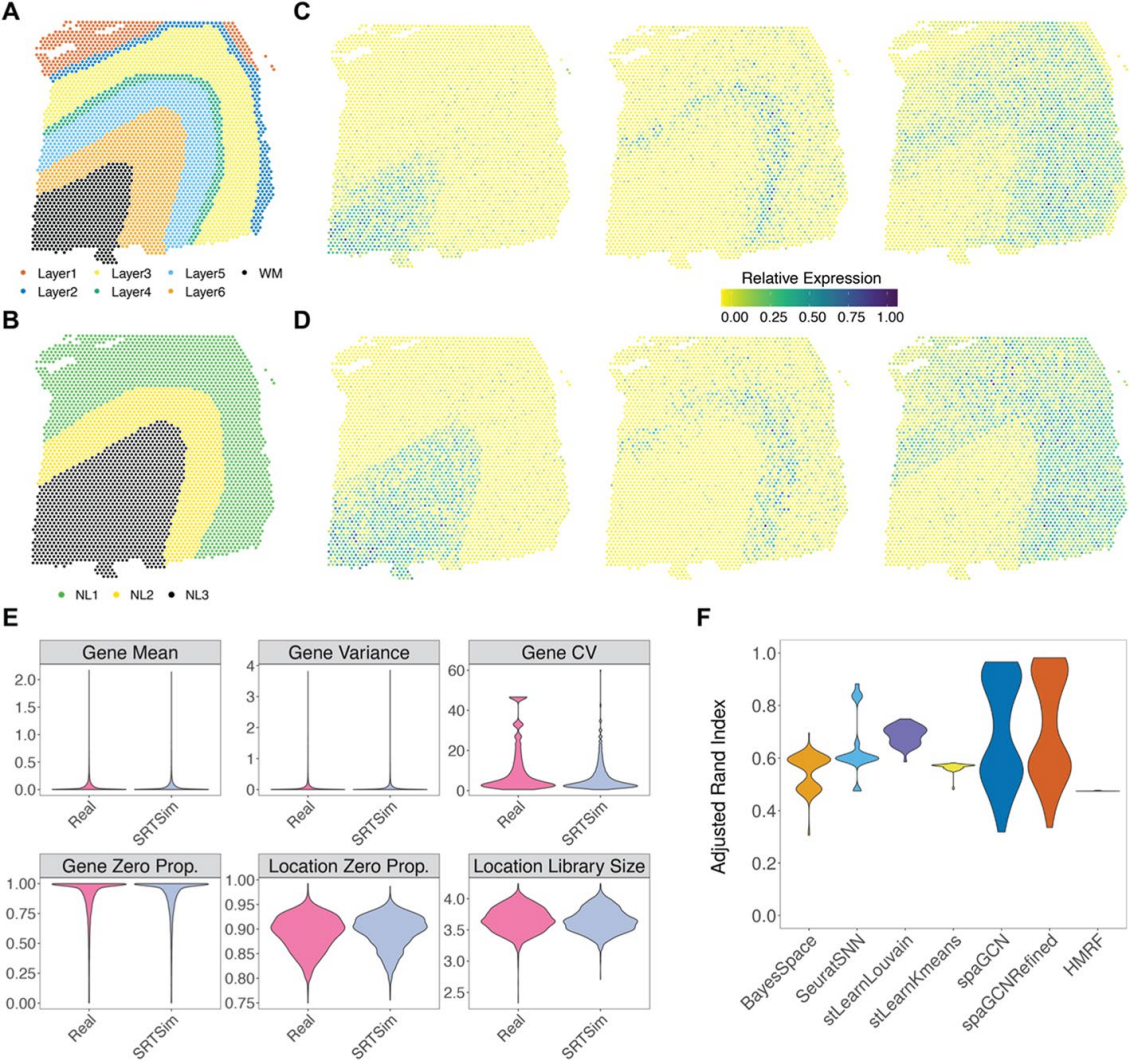
Benchmarking spatial clustering methods using synthetic data from SRTsim based on the DLPFC reference data with redesigned layers. **A** The reference DLPFC data contains seven layers, including six DLPFC layers and white matter (WM). Sample 151673 in the DLPFC data is used to serve as the reference. **B** The spatial distribution of three redesigned layers. NL1 corresponds to the original layers 1–3. NL2 corresponds to the original layers 4–5. NL3 corresponds to the original layer 6—white matter. **C** The spatial expression patterns of three representative genes (*MOBP*, *PCP4*, *SNAP25*) are displayed in the reference data. **D** Spatial expression patterns of three representative genes are displayed in the synthetic data with redesigned layers (nearest neighbors k=20). **E** Violin plots show the distributions of six metrics (six panels) in the reference data (real; *x*-axis) and in the synthetic data generated by SRTsim (*x*-axis). The six metrics include four gene-wise metrics (expression mean; variance; coefficient of variation, cv; and zero proportion) and two location-wise metrics (zero proportion and library size). **F** The performance of six spatial clustering methods (*x*-axis) is evaluated based on the adjusted Rand index (*y*-axis) using synthetic data with redesigned layers generated from SRTsim

Finally, we carried out simulations to examine the influence of the number and size of tissue domains on the performance of spatial clustering. In particular, we used a modified version of the domain-specific simulations to create synthetic data based on DLPFC, where the modification allows us to change the number and size of the tissue domains. To do so, we merged the original seven cortical layers (Fig. 5A) into three new layers (Fig. 5B). We designed the simulation such that the genes on each new layer inherit the spatial expression pattern from one of the original layers. Specifically, the new layer NL1 contains the original layers 1–3 and displays a similar expression pattern as layer 3; the new layer NL2 contains the original layers 4–5 and displays a similar expression pattern as layer 5; the new layer NL3 contains the original layer 6 and white matter and displays similar expression pattern of the white matter layer. The synthetic data preserved the expression characteristics of the reference data (Fig. 5E) and the spatial expression patterns of the marker genes (Fig. 5C, D). With this simulation, the stLearn with Louvain achieves the highest ARI on average (mean = 0.70, median = 0.69, Fig. 5F). The performance of the stLearn with Louvain is followed by refined SpaGCN (mean = 0.70, median=0.60) and SpaGCN (mean = 0.69, median = 0.59). The results suggest that the performance of different spatial clustering methods depends on the number and size of tissue domains. Note that the ARI from SpaGCN displays a bimodal pattern, with relatively high values in five replicates with slightly clearer spatial domain patterns and relatively low ARI in five remaining replicates with slightly less clear spatial domain patterns (Additional file 1: Fig. S16), suggesting the potentially sensitive performance of SpaGCN across replicates.

#### Application 2: Benchmarking SE analysis methods using synthetic SRT data

**Fig. 6:**
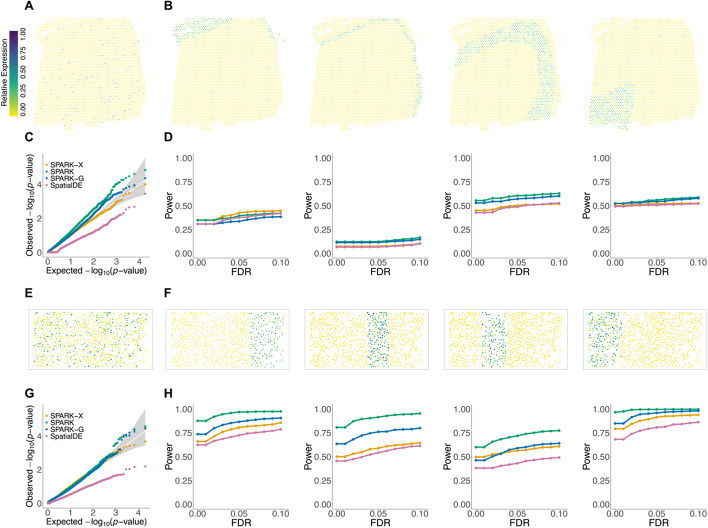
Benchmarking spatial expression analysis methods using synthetic data from SRTsim. Synthetic data are either generated based on the DLPFC reference (**A–D**) or the STARmap reference (**E–H**). **A** A representative gene in the DLPFC reference data (sample 151673) displays a random spatial expression pattern. **B** Representative genes in the DLPFC reference data display four distinct spatial expression patterns corresponding to four different layered structures (Layer1, Layer 2, Layer 3, and WM, respectively). **C** Quantile–quantile plot of the observed −log_10_(*P*) from different methods against the expected −log_10_(*P*) under the null SRTsim simulations based on the DLPFC reference. *P* values were combined across ten simulation replicates. **D** Power plots show the proportion of true positives (*y*-axis) detected by different methods at a range of FDRs (*x*-axis) for the alternative SRT simulations based on the DLPFC reference. Each panel corresponds to the spatial expression pattern displayed in **B**. SE strength is set to be *five-fold*. **E** A representative gene in the STARmap reference data displays a random spatial expression pattern. **F** Representative genes in the STARmap reference data display four distinct spatial expression patterns that correspond to four different layered structures (Layer2/3, Layer 4, Layer 5, and Layer 6, respectively). **G** Quantile–quantile plot of the observed −log_10_(*P*) from different methods against the expected −log_10_(*P*) under the null SRTsim simulations based on the STARmap reference. *P* values were combined across ten simulation replicates. **H** Power plots show the proportion of true positives (*y*-axis) detected by different methods at a range of FDRs (*x*-axis) for the alternative SRT simulations based on the STARmap reference. Each panel corresponds to the spatial expression pattern displayed in **F**. SE strength is set to be *three-fold*

We further demonstrated the utility of SRTsim in benchmarking spatial expression (SE) analysis methods that aim to identify genes with spatial expression patterns. Here, we used synthetic data generated by SRTsim for benchmarking four SE methods that include SPARK [27], SPARK-G, SPARK-X [25], and SpatialDE [24]. To make simulations as realistic as possible, we simulated the data based on the parameters inferred from the same two datasets used in the previous section: the 10x Visium DLPFC data and the STARmap data (see details in the “Methods” section). Rather than preserving the gene-specific spatial expression pattern as shown in the reference-based simulations in the previous section, the synthetic data here are generated in a reference-free fashion to create artificial spatial expression patterns of interest (even though we used the tissue shape profile from the reference). For each data, we considered a set of null simulation settings and a set of alternative simulation settings, with 1000 simulated genes in each setting. Under the null settings, all genes are non-SE genes with expression levels randomly distributed across spatial locations without any spatial expression patterns (Fig. 6A, E). Under the alternative settings, 900 genes are non-SE genes while 100 genes are SE genes with domain-specific spatial expression patterns. In particular, the SE genes display one of the seven spatial patterns corresponding to each spatial domain for the 10x Visium-based simulations (Fig. 6B and Additional file 1: Fig.S17A) and show one of the four spatial patterns corresponding to each spatial domain for STARmap-based simulations (Fig. 6F). For each alternative simulation setting, we consider two scenarios: a scenario where the SE genes display strong spatial expression patterns and another scenario where the SE genes show weak spatial expression patterns. With the synthetic data, we applied different SE methods to identify SE genes in different simulation settings. In total, we examined two null settings and 22 alternative settings (Additional file 1: Table S6, details in Methods). We examined type I error control under the null and examined power under the alternatives.

In the two null simulation settings based on the two datasets, SPARK, SPARK-G, and SPARK-X produce well-calibrated *P* values, while SpatialDE yields overly conservative *P* values (Fig. 6C, G). As SpatialDE produced overly conservative *P* values under the null, we measured power in the alternative simulations based on false discovery rate (FDR) to ensure fair comparison among methods. In the alternative simulations based on DLPFC, we found that SPARK and SPARK-G are more powerful than SPARK-X and SpatialDE for most spatial expression patterns, except for the layer 1 specific expression pattern for which SPARK-X is more powerful than the other three methods. In the simulations, we found that the power of SE analysis depends on two critical factors: the SE strength and the size of the tissue domain on which the SE gene displays a domain-specific expression pattern. For example, it is challenging to detect genes that display layer-specific expression patterns in the thin layers 2 and 4 compared to the other wider layers (Fig. 6D and Additional file 1: Fig. S17B), unless the SE expression pattern is strong (Additional file 1: Fig. S17C). Similar conclusions hold in the alternative simulations based on the STARmap data. For example, SPARK outperforms all three other methods in detecting SE genes across all patterns (Fig. 6H and Additional file 1: Fig. S18B). All methods achieve higher power in detecting genes that display layer-specific expression patterns in the thin layer 4 compared to the wide layer 5. Overall, SRTsim allows us to effectively evaluate the performance of different SE analysis methods using synthetic data that highly resembles the real data but with known underlying truth.

#### Application 3: Benchmarking cell-cell communication identification methods using synthetic SRT data

We performed comprehensive and realistic simulations to evaluate the performance of Giotto [37] and CellphoneDBv3 [61]. The simulation details are provided in the “Methods” section. Briefly, we first estimated the shape profile based on the spatial locations of the reference data and randomly generated 5000 locations within the estimated shape profile to serve as the single cell locations. We assumed that these single cells belong to four different cell types and created four equal size regions on the tissue with distinct cell type compositions in each region. In parallel, we simulated expression counts for 5000 genes and 2000 single cells from the four cell types in two ways. In the first way (homogenous expression setting), we simulated the expression level for each gene for all cells from a common negative binomial distribution and randomly assigned these cells into four cell types. In the second way (heterogeneous expression setting), in each simulation replicate, we randomly selected four cell types in the STARmap dataset and simulated the expression level for each gene in each cell type using a cell type-specific negative binomial distribution, with parameters estimated based on the particular cell type in the reference data. We then assigned the simulated cells onto the single-cell locations of the regions based on the specified cell type composition in each region to create a spatial transcriptomics dataset at single-cell resolution. We finally randomly assigned ligand-receptor (L-R) gene pairs expressed in cell type pairs (A-B) to the simulated cells. We compared the performance of four methods including Giotto with spatial information, Giotto without spatial information, CellphoneDBv3 with spatial information, and CellphoneDBv3 without spatial information.

**Fig. 7:**
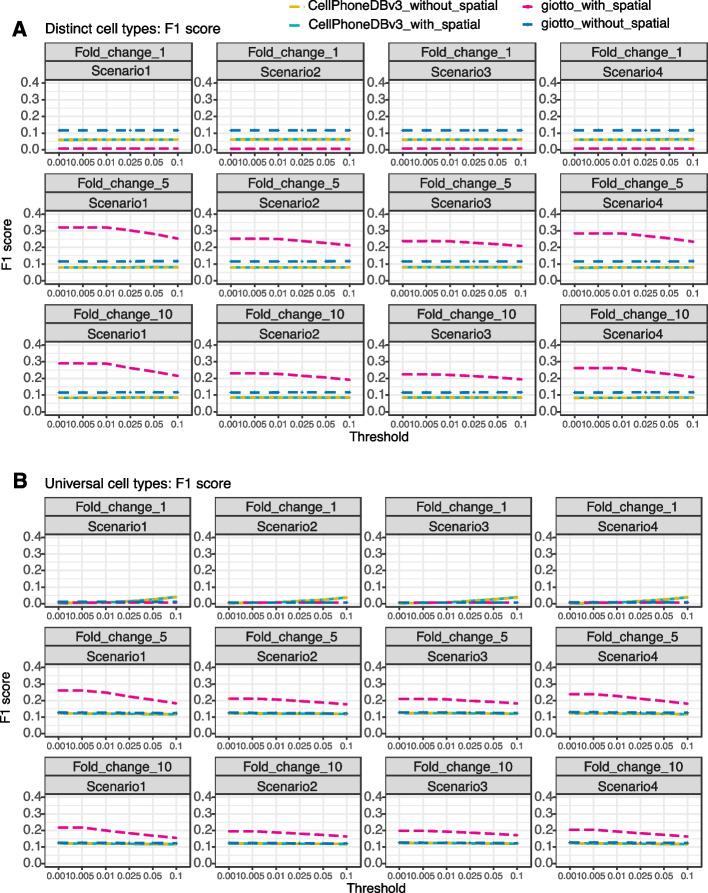
Benchmarking cell-cell communication identification methods using synthetic data from SRTsim. **A** The power of different methods for identifying cell-cell communications is measured by mean F1 scores across a range of adjusted *p*-value thresholds in the heterogeneous expression settings. Compared methods include CellphoneDBv3 with spatial information (green dash line), CellphoneDBv3 without spatial information (yellow dash line), Giotto with spatial information (pink dash line), and Giotto without spatial information (blue dash line). Results are shown for four different scenarios (columns) with different cell-cell communication strength (rows). **B** The power of different methods for identifying cell-cell communications is measured by mean F1 scores across a range of adjusted *p*-value thresholds in the homogeneous expression settings. Results are again shown for four different scenarios (columns) with different cell-cell communication strengths (rows). Each setting includes 10 simulation replicates

Overall, we found that Giotto with spatial information achieves the best performance. Specifically, in the heterogeneous expression settings, in the absence of interaction (fold change=1), Giotto with spatial information achieves the lowest F1 scores. For example, at a *p* value threshold of 0.05, the mean F1 score for Giotto with spatial information is 0.007, 0.006, 0.007, and 0 for the four scenarios, respectively. In contrast, Giotto without spatial information has the highest F1 score (mean=0.117, 0.117, 0.117, and 0.117 for the four scenarios, Fig. 7A). The CellphonDBv3 with spatial information (mean=0.085, 0.085, 0.086 and 0.085) and CellphoneDBv3 without spatial information (mean=0.086, 0.085, 0.086, and 0.084) have similar mean F1 score values. In the presence of moderate cell-cell communications, Giotto with spatial information achieves the highest F1 scores (mean=0.204, 0.188, 0.191, and 0.196 for four scenarios, Fig. 7A), demonstrating its power. Giotto without spatial information (mean=0.117, 0.117, 0.116, and 0.117) performs slightly better than CellphoneDBv3 with spatial information (mean=0.081, 0.08, 0.082, and 0.079) and without spatial information (mean=0.08, 0.081, 0.08, and 0.08, Fig. 7A). The two methods of CellphoneDBv3 perform similarly and are worse than the two Giotto methods, possibly because CellphoneDBv3 only incorporates regional spatial information without fully utilizing cell-cell spatial proximity as used in Giotto with spatial information. Finally, in the presence of high cell-cell communications, Giotto with spatial information also achieves the highest F1 score (Fig. 7A). The results are somewhat similar in the homogenous expression settings (Fig. 7B).

While Giotto with spatial information achieves the best performance in terms of F1 score, we note that all methods can be advantageous when measured with the other metrics. In particular, Giotto with spatial information achieves the highest precision (Additional file 1: Figs. S21A-B); Giotto without spatial information achieves the highest recall (Additional file 1: Figs. S19A-B); and the two CellphoneDBv3 methods generally achieve the highest specificity (Additional file 1: Figs. S20A-B).

## Discussion

We have presented SRTsim, an efficient and flexible framework for reproducible simulations of SRT data. We have benchmarked SRTsim along with eight scRNA-seq simulators using 49 SRT datasets from eight distinct SRT platforms, demonstrating the ability of SRTsim in capturing various gene expression characteristics in real data. Importantly, SRTsim is the only current method capable of preserving the spatial gene expression patterns observed on the tissue, facilitating SRT-specific applications. SRTsim is also computationally efficient and is an order of magnitude faster than scDesign2 and ZINB-WaVE, which are the only two scRNA-seq simulators capable of preserving certain gene expression characteristics in real data. In addition, SRTsim is well-documented and reproducible, with a simulation strategy that is independent of any specific SRT-analysis method, ensuring transparent, reproducible, and fair comparisons across SRT methods. We have illustrated the benefits of SRTsim in assessing the performance of spatial clustering methods, spatial expression analysis methods, and cell-cell communication identification methods.

SRTsim relies on four popular count models that include Poisson, ZIP, NB, and ZINB for generating the synthetic data. Two of the count models, ZIP and ZINB, are zero-inflated models that model zero-inflation not accounted for on top of the Poisson and NB models, respectively. While previous scRNA-seq literature has observed excessive dropouts in non-UMI datasets and subsequently recommended the modeling of zero-inflation, recent studies have shown that modeling zero-inflation is not necessary for UMI-based scRNA-seq datasets [62]. Similarly, studies in SRT have also shown that modeling zero inflation may not be necessary for the majority of spatial transcriptomics datasets [57], as excessive zero values in those data can often be accounted for by over-dispersion rather than zero-inflation on top of the Poisson model. Consequently, using the NB model or other types of over-dispersed Poisson models such as the Poisson mixed model [63, 64] is sufficient for effective SRT simulation for the majority of genes in almost all spatial transcriptomics techniques, with the only exception of seqFISH+. Indeed, ZINB is the preferred model for more than 60% of genes in the two seqFISH+ datasets, suggesting distinctive data features of seqFISH+.

In the analysis, we found that HMRF performs poorly in the synthetic data generated from DLPFC but reasonably well in the synthetic data generated from STARmap data. The performance disparity of HMRF is presumably due to two reasons. First, the two data are of different sparsity: the DLPFC data has 93.4% zero entries in the gene expression matrix, while the STARmap data only has 69.5%. The high sparsity in the gene expression could violate the Gaussian assumption of HMRF and lead to poor performance in the DLPFC data. The second reason is that the DLPFC tissue structure is more complex than the STARmap tissue structure. In particular, DLPFC data was collected in a large capture area (6.5×6.5mm) that contains 7 spatial domains, while STARmap data was collected in a much smaller capture area (11.78×11.78um) with only 4 spatial domains. Because HMRF relies on a Potts model to incorporate neighborhood similarity in the spatial domain assignment, it may perform well when the tissue structure is simple and small but less so when the tissue structure is large and complex.

We have demonstrated SRTsim’s ability to generate synthetic datasets resembling the real data collected from eight different SRT techniques. These techniques include the three commonly applied commercial platforms such as 10x Visium, NanoString CosMx SME, and Vizgen MERFISH. We note that another SRT commercial platform is NanoString GeoMx DSP [65–67], which is primarily used to measure gene expression on a few targeted regions of interest (ROI). Because each ROI from GeoMx is relatively large and contains many cells, the GeoMx data likely resemble bulk RNAseq. Consequently, GeoMx data may be simulated directly using standard bulk RNA simulators, potentially with some additional modifications to account for the general correlation structure among different ROIs. Therefore, we did not explore the simulations for GeoMx data in the present study.

One important benefit of SRTsim is its ability to produce realistic synthetic datasets under different parameter settings for evaluating the performance of various SRT-specific methods in a cost-effective fashion. We have illustrated such benefit by using SRTsim to assess the performance of spatial clustering methods, spatial expression analysis methods, and cell-cell communication identification methods. Importantly, SRTsim is not limited to these three applications and can be used for method evaluation in many other SRT analytic tasks. For example, one can generate synthetic SRT data with continuous expression programs using the reference-free module in SRTsim for evaluating trajectory analysis in the spatial context [40, 68]. In addition, one can generate synthetic SRT data using SRTsim with the single-cell resolution for spatial charting analysis [69, 70]. Certainly, SRTsim focuses directly on simulating gene expression data without explicitly modeling the underlying cell type composition and is thus not applicable for evaluating certain SRT analytical tasks such as cell type deconvolution [29]. Subsequently, an important future direction of SRTsim is to incorporate cell type composition into the SRT simulation framework, while properly accounting for the spatial correlation of cell type composition across tissue locations. Overall, SRTsim represents an effective SRT simulation framework that is expected to benefit and facilitate the rapid development of computational methods for various SRT-specific analytic tasks, in a similar way as scRNA-seq simulators have benefited the development of scRNA-seq methods and systematic benchmark studies there [71–74].

While we have primarily focused on using synthetic data from SRTsim to evaluate computational method performance, we note that SRTsim also has the potential to guide experimental design. For example, in the spatial clustering application, we have explored how the accuracy of spatial clustering for different computational methods may change under the influence of sequencing depth and the number of spatial locations measured on the tissue. In that setting, we showed that SRTsim could help us identify the likely optimal sequencing depth and the number of measured locations for detecting spatial domains on the mouse cortex, thus facilitating experimental design for maximizing the effectiveness of spatial transcriptomics.

## Conclusions

In conclusion, SRTsim is an effective simulation tool for SRT studies. SRTsim provides scalable, reproducible, and realistic simulations, while preserving expression characteristics and spatial patterns. We have demonstrated the utility of SRTsim in evaluating the performance of spatial clustering methods, spatial expression analysis methods, and cell-cell communication identification methods.

## Methods

### The SRTsim framework

SRTsim is a general and flexible computational framework for simulating gene expression count data for spatial transcriptomics. SRTsim primarily performs reference-based simulations where the simulated gene expression resembles a reference spatial transcriptomics data supplied by the user. SRTsim can also perform reference-free simulations without using a reference dataset, which will be described in detail later. In the reference-based simulations, SRTsim relies on the reference spatial transcriptomics data to obtain the spatial coordinates of the measured locations, create a new set of tissue locations for a user-specified location number if needed, and simulate expression count data based on the parameters estimated in the reference data. SRTsim provides two simulation options. The first option is a tissue-based simulation, where the expression data are directly simulated on all spatial locations of the entire tissue. The second option is a domain-specific simulation, where the expression data are first simulated on locations within each tissue domain separately before being combined across domains. Like existing scRNA-seq simulators, the gene expression data simulated by SRTsim resembles the gene expression pattern in the reference data based on a wide variety of count characteristics. Unlike existing scRNA-seq simulators, the expression data simulated by SRTsim also preserves the spatial expression pattern of the reference data. The spatial expression pattern is preserved either on the entire tissue for tissue-based simulations or on each specific tissue domain for domain-specific simulations. The reference-based simulations in SRTsim consist of the following three steps:

- Step 1. Obtain or create location coordinates in the synthetic data. This is achieved by either directly obtaining the coordinates of the tissue locations measured in the reference data to serve as the locations in the synthetic data, or, if requested by the user, creating new locations in the synthetic data with the number of locations specified by the user. To create new locations, SRTsim first fits the measured locations in the reference data using a concave hull algorithm [75] to obtain a list of measured locations that define the outskirt of the tissue in the shape of a polygon. SRTsim then creates a set of new locations within the polygon using two different approaches chosen by the user. In the first approach, SRTsim creates a square grid within the polygon, with the number of the grid points inside the polygon determined by the user. In the second approach, SRTsim uses a random point process to create locations within the polygon using the R function *spsample*, again with the number of locations determined by the user. The first approach mimics the spatial location layout in certain spatial transcriptomics technologies such as 10x Visium while the second approach mimics the spatial location layout in certain spatial transcriptomics technologies such as MERFISH or seqFISH+.

Once the coordinates for the locations are obtained or created in the synthetic data, the user can then proceed to perform either tissue-based or domain-specific simulations. For tissue-based simulations, only the location coordinates are needed for the next two steps described below. For domain-specific simulations, SRTsim also requires the user to input the tissue domain labels for the measured locations in the reference data. The input tissue domain labels are either directly used when the locations in the synthetic data are the same as the measured locations in the reference data or are used to extrapolate the tissue domain labels for the newly created locations in the synthetic data. In the latter case, for each new location, SRTsim assigns its domain label based on the domain labels from the *k* (default = 3) nearest measured locations through a Boyer-Moore majority voting algorithm. The domain labels are then used along with the location coordinates in the next two steps for the domain-specific simulations.

- Step 2. Infer the gene-specific expression count model based on the reference data and generate the expression counts for each gene in the synthetic data. For tissue-based simulations, SRTsim uses all measured locations on the entire tissue to infer the expression model. For domain-specific simulations, SRTsim uses the measured locations on each tissue domain to infer domain-specific expression models. In either case, SRTsim examines one gene at a time and fits the reference expression count data using four different count models. The four count models include the Poisson model, the zero-inflated Poisson model (ZIP), the negative binomial model (NB), and the zero-inflated negative binomial model (ZINB). After modeling fitting, SRTsim selects the model with the lowest Akaike information criterion (AIC) as the gene-specific count model and uses the estimated model parameters to generate expression counts for the gene of focus in the synthetic data. The expression counts are generated for all locations on the entire tissue in the tissue-based simulations or locations on each tissue domain in turn in the domain-specific simulations.
- Step 3. Assign the generated gene expression counts to the locations in the synthetic data in a way that preserves the spatial expression pattern of every gene. To preserve the spatial expression pattern, SRTsim first rank orders the locations in the synthetic data for every gene based on its expression level across locations in the reference data. Afterwards, SRTsim assigns the simulated gene expression to the locations in the synthetic data based on the obtained rank order. Specifically, in the setting where the locations in the synthetic data are identical to the locations in the reference data, SRTsim directly ranks the locations in the synthetic data based on the reference and assigns the simulated expression counts to locations in the synthetic data based on the rank. In the setting where new locations are created in the synthetic data, SRTsim first identifies for each new location the *k* (default = 3) nearest neighboring locations measured in the reference data, randomly samples an expression count for the gene of focus from the identified neighbors, assigns the sampled count to the new location, ranks all new locations in the synthetic data based on the assigned reference expression counts, and finally assigns the simulated expression counts to the new locations based on the resulting rank. This way, the spatial expression pattern of the gene of focus in the synthetic data will mimic the spatial expression pattern of the same gene in the reference data, thus preserving the spatial expression pattern. In the tissue-based simulations, the ranking of locations and the assignment of the simulated expression counts are performed for all locations on the entire tissue. In the domain-specific simulations, the ranking of locations and the assignment of the simulated expression counts are performed on each tissue domain in turn.

In the reference-based simulations, besides preserving the spatial expression pattern of the gene of focus, SRTsim can also allow the user to redesign the spatial expression pattern of the gene in any target tissue region in the synthetic data by extrapolating the spatial expression pattern of the gene from another tissue region in the reference. To do so, SRTsim first uses a concave hull algorithm to obtain the shape profile for the target region, which consists of a set of measured locations that define the outskirt of the target tissue region in the synthetic data. In addition, SRTsim obtains the shape profile for the reference region, which consists of a set of measured locations that define the outskirt of the reference tissue region. SRTsim then maps the estimated shape profile of the reference region to the shape profile of the target region through the affine transformation [76], projects the spatial locations inside the reference region into a new set of projected locations inside the target region using the calculated affine transformation matrix, and directly assigns the reference expression counts of the reference locations to the projected locations. The projected locations then served as the new reference locations for the target region. In particular, for each location in the target region in the synthetic data, SRTsim identifies the *k* (default = 3) nearest neighboring projected locations, randomly samples an expression count for the gene of focus from the identified neighbors, assigns the sampled count to the location of focus, ranks all locations in the target region in the synthetic data based on the assigned projected reference expression counts, and finally assigns the simulated expression counts to locations in the target region based on the resulting rank. This way, the spatial expression pattern of the gene of focus in the target region will mimic the spatial expression pattern of the same gene in the reference region, thus creating potentially new and realistic spatial patterns on the tissue.

Besides reference-based simulations, SRTsim can also perform reference-free simulations without using a reference spatial transcriptomics dataset. To do so, SRTsim allows the user to first create an arbitrary tissue shape in two different ways: the user can either choose from a set of customizable shapes that are constructed based on various circles and squares, or provide a shape profile in the form of a set of location coordinates that define the outskirt of the desired tissue shape. With the defined tissue shape, SRTsim creates spatial locations on the tissue based on a user-specified location number. The spatial locations are then created either through a grid-based approach or a random sampling-based approach, as detailed in Step 1. For domain-specific simulations, the user is also required to assign a tissue domain label for each spatial location and specify a domain-specific fold change parameter that defines the mean expression fold-change for gene expression inside the domain versus that on the entire tissue. SRTsim then simulates the expression count data for one gene at a time on each location based on a user-specified count model. The user has the option to choose one of the four count models described in Step 2 and provide a set of model parameters that include the mean parameter (for all models), the dispersion parameter (for the two over-dispersed models), and the zero-proportion parameter (for the two zero-inflated models). Afterwards, SRTsim allocates the simulated expression counts to locations either randomly across all locations on the entire tissue for tissue-based simulations or randomly across locations within each tissue domain for domain-specific simulations. Note that the genes in the reference-free simulations are restricted to display domain-specific expression patterns but not general spatial expression patterns obtainable in reference-based simulations. Importantly, all reference-free simulations can be carried out in an R shiny framework, which allows the user to easily visualize the simulated spatial transcriptomics data.

For both reference-based and reference-free simulations, the output is an S4 object that mainly contains two matrices for the synthetic data: one matrix contains the spatial coordinates for all tissue locations, along with their domain labels for domain-specific simulations; and the other matrix contains the simulated expression counts for all genes across all locations. The output S4 object also contains all the estimated model parameters and user-defined parameter inputs, which, when paired further with the input random seed, ensure the reproducibility of the synthetic data. After the simulation, user can also compare the synthetic dataset to the corresponding reference dataset using functions implemented in the package.

Both reference-based and reference-free simulation procedures are implemented in an R CRAN package, SRTsim. The R package contains the R shiny app, which can be easily installed and accessed on any local computer. The reference-free simulations in SRTsim can also be carried out through an online shiny app available at https://jiaqiangzhu.shinyapps.io/srtsim, which is powered by RStudio. The software SRTsim, along with the scripts for reproducing all results in the present study, is freely available at www.xzlab.org/software.html.

### Real data-based simulations and benchmarking

We obtained 49 SRT datasets across eight different spatial transcriptomics platforms (Additional file 1: Table S1). Among them, 22 datasets are generated with the 10x Visium technique, including 10 obtained from the 10x Visium spatial gene-expression repository on various tissues and 12 obtained from the spatialLIBD study on the human dorsolateral prefrontal cortex [17]. Two datasets are generated with the original spatial transcriptomics technique, including one collected on a human breast cancer tissue and the other collected on the mouse olfactory bulb [5]. Eleven datasets are generated with DBiT-seq on various tissues [9]. Five datasets are generated with MERFISH on various tissues. Five datasets are generated with CosMx SMI on the human NSCLC tissue [58]. Two datasets are generated with seqFISH+, including one on the mouse olfactory bulb and the other on the mouse cortex [2]. One dataset is generated with STARmap on the mouse visual cortex [13]. One dataset is generated with SlideseqV2 on the mouse embryo [8]. The number of genes in these datasets ranges from 140 to 25,072, and the number of locations ranges from 251 to 51,649. We used all 45 SRT datasets for reference-based simulations.

We benchmarked SRTsim against eight existing single-cell simulators that include ZINB-WaVE (implemented in Splatter v.1.14.1), SPARSim (v.0.9.5), SymSim (v.0.0.0.9000), two variations of scDesign2 (with/without copula, v.0.1.0), and three variations of Splat in the Splatter package (Splat Simple, Splat, and Kersplat, v1.14.1). These single-cell simulators allow users to perform reference-based simulations with provided single-cell reference data. We supplied each of the 40 SRT datasets one at a time to serve as the reference data and applied these simulators to simulate spatial transcriptomics. We evaluated the performance of different simulators by six metrics that include four gene-specific metrics and two location-specific metrics. The gene-specific metrics measure for each gene how similar the simulated gene expression counts resemble that of the reference data across locations. The four gene-specific metrics include expression mean, variance, coefficient of variation, and the proportion of zero counts. The location-specific metrics measure for each location how similar the simulated gene expression counts resemble that of the reference data across genes. The two location-specific metrics include the proportion of zero counts and the library size. We examined the empirical distribution of each gene-specific metric across genes and the distribution of each location-specific metric across locations. In addition, we examined whether the simulated gene expression counts preserve the spatial expression pattern of the reference gene through visualization. We also examined the computational efficiency of different simulators by recording the computation time and memory usage and examined their stability by recording the number of datasets each simulator failed. For computation time and memory usage, we focused on eight datasets, one from each spatial transcriptomics platform: ST (Rep11_MOB), 10x Visium (LIBD_151673), DBiT (GSM4189615_0719cL), STARmap (visual_1020), seqFISH+ (cortex_svz), CosMx SMI (Lung12_FOV1), MERFISH (HumanOvarianCancerPatient1_FOV1051), and SlideseqV2 (Puck_190926_03). The number of genes in these seven datasets ranges from 550 to 23,124, and the number of locations ranges from 262 to 51,649.

### Evaluating spatial clustering performance based on simulations

We illustrate the benefits of SRTsim for benchmarking spatial domain detection methods using simulations. To do so, we obtained two reference SRT datasets for simulations: a non-single-cell resolution 10x Visium dataset, which has relatively low gene expression counts with the median value of the mean gene-specific expression count across genes being 0.03, and a single-cell resolution STARmap dataset, which has relatively moderate gene expression counts with the median value of the mean gene-specific expression count across genes being 0.38.

The 10x Visium dataset is a human dorsolateral pre-frontal cortex (DLPFC) data obtained from spatialLIBD and has been widely used for benchmarking studies [36, 39, 59]. For this dataset, we obtained the sample 151673, with 21,842 genes measured on 3639 locations on the tissue. The majority of these locations (*n*=3611) were clustered by the original study based on cytoarchitecture and gene markers into seven tissue domains that include six dorsolateral prefrontal cortex (DLPFC) layers and the white matter (Fig. 3A). We focused on the locations with known domain annotations and discarded the remaining unannotated locations (*n*=28) for analysis.

The STARmap dataset is a primary visual cortex data, with 1020 measured genes on 930 cells. Following the annotations based on the spatial expression pattern of gene markers in [13, 77], we focused on the 673 cortical layer cells in L2-L6 and treated the layer annotations as the ground truth for evaluating the performance of spatial clustering methods.

For either dataset, we performed both tissue-based and domain-specific simulations. In the domain-specific simulations, in addition to directly using the tissue domains from the reference data, we also examined an alternative simulation scenario where we varied the number of tissue domains and their sizes while maintaining their domain-specific spatial gene expression pattern. To do so, we focused on the DLPFC data and grouped the original seven cortical layers into three new layers (NL1, NL2, and NL3; Fig. 4B). NL1 corresponds to the original layers 1–3 and has a spatial expression pattern extrapolated from the original layer 3. NL2 corresponds to the original layers 4–5 and has a spatial expression pattern extrapolated from the original layer 5. NL3 corresponds to the original layer 6—white matter and has a spatial expression pattern extrapolated from the original WM. We performed ten simulation replicates for each of the five simulation scenarios (2 datasets × 2 types of simulations + 1 alternative simulation with modified domain number and size).

To guide experimental design, we also performed additional domain-specific simulations using the DLPFC data. In particular, we considered three different scenarios, where we examined the influence of three critical experimental parameters on the accuracy of different spatial clustering methods in detecting spatial domains. In scenario I, we examined the influence of overall sequencing depth, where we varied the overall sequencing depth while keeping the number of spatial locations constant. In this scenario, we explored five choices for the overall sequencing depth (8.3, 16.6, 24.9, 33.2, and 49.8 million), representing 50%, 100%, 150%, 200%, and 300% of the observed sequencing depth of the reference data, respectively. In scenario II, we examined the influence of the measured location number, where we varied the number of locations on the tissue but kept the overall sequencing depth constant. In this scenario, we varied the number of spatial locations on the tissue to be 100, 300, 1000, 2000, 3611 (=observed location number in the reference), and 5000. In scenario III, we varied both the number of locations and the overall sequencing depth but kept the average number of reads in each spatial location fixed. In this scenario, we examined all pairs of combinations based on the six location number choices in scenario II and five overall sequencing depth choices in scenario I.

We evaluated the performance of six different spatial clustering methods in detecting spatial domains on the tissue in the synthetic data. The six evaluated methods include the SNN (shared nearest neighbor) implemented in Seurat (v.4.0.1), BayesSpace (v.1.2.0), stLearn with Kmeans, stLearn with Louvain (v.0.3.2), SpaGCN (v.1.0.0), and HMRF (implemented in Giotto, v.1.0.3). For stLearn, its two methods would ignore the spatial location information and perform regular Louvain and Kmeans clustering, as the synthetic data do not contain corresponding histological images. For Seurat and Louvain, we examined a range of resolution values and chose the one value that leads to the number of clusters closest to the truth. For SpaGCN, we also included the results with the cluster refinement (refined-SpaGCN) in the DLPFC-based simulation, which utilizes the array coordinates on top of the pixel coordinates information. For HMRF, we varied the spatial parameter beta (from 0 to 100, with an increment of 5) and only reported the results for the beta value that achieves the highest performance in the fitted data—because of this, the results from HMRF may represent model over-fitting and may over-state its performance. BayesSpace is developed specifically for ST/Visium platforms, where the neighborhood structures are well-defined in either square or hexagonal arrangements. Therefore, when applied to the STARmap data, where the spatial locations are randomly distributed, BayesSpace can only perform regular mclust-based clustering. Following [17], we measured spatial clustering performance using the adjusted Rand index (ARI) [78, 79], which quantifies the similarity between the inferred spatial cluster labels and the ground truth. For each method, we used ten different random seeds to account for algorithmic variability in each simulation replicate and obtained ten ARI values per replicate.

### Evaluating spatial expression analysis with simulations

We also illustrate the benefits of SRTsim for benchmarking spatial expression analysis methods using simulations. To do so, we performed similar reference-based simulations using the two reference datasets (DLPFC and STARmap). For each reference data, we considered two simulation settings: a null setting where we simulated 1000 non-SE genes with random spatial expression patterns and an alternative setting where we simulated 100 SE genes that display spatial expression patterns along with 900 non-SE genes with random spatial expression patterns. Here, we used a negative binomial model to simulate the expression counts for both SE and non-SE genes, following the recommendations of [24, 27, 57] for spatial expression analysis. In the model, we set the dispersion parameter for all genes to be the global dispersion parameter estimated in the corresponding data (0.3 for DLPFC and 0.35 for STARmap). We set the mean parameter for the non-SE genes as the median of the mean gene-specific expression level across genes in the corresponding data (0.03 for DLPFC and 0.4 for STARmap). In addition, we set the mean parameter for the SE genes differently in each tissue domain to create different simulation scenarios. Specifically, for 10x Visium-based simulations, we considered seven simulation scenarios, each representing a layer-specific expression pattern for one of the seven cortical layers. In each scenario, we set the mean parameter for locations outside the layer of focus to be 0.03 and set the mean parameter for locations inside the layer to be either 5 or 10 times higher than (for 50 SE genes) or 1/5 or 1/10 of (for 50 SE genes) that for the locations outside the layer, representing low (5 or 1/5) or high (10 or 1/10) SE signal strength, respectively. For STARmap-based simulations, we considered five simulation scenarios, each representing a layer-specific expression pattern for one of the five cortical layers (L2–L6). In each scenario, we set the mean parameter for locations outside the layer of focus to be 0.4 and set the mean parameter for locations inside the layer to be either 3 or 4 times higher than (for 50 SE genes) or 1/3 or 1/4 of (for 50 SE genes) that for the locations outside the layer, representing low (3 or 1/3) or high (4 or 1/4) SE signal strength, respectively. Note that the SE strength in the STARmap-based simulations is lower than that in the DLPFC-based simulations—this is because the expression levels are lower in DLPFC, and thus strong SE strengths are needed for all methods to have sufficient power there.

We evaluated the performance of four SE methods for identifying genes with spatial expression patterns. The four SE methods include SPARK (v.1.1.1), which relies on an over-dispersed Poisson model; SPARK-G, which relies on Gaussian approximation to SPARK; SPARK-X, which uses a non-parametric test; and SpatialDE (v.1.1.3), which relies on a linear mixed model. The first three methods are implemented in the R package SPARK while the last method is implemented in the SpatialDE python package. Besides these four methods, we also explored three other SE methods that include trendsceek [28], BOOST-GP [80], and SOMDE [26]. However, both trendsceek and BOOST-GP incurred a heavy computational burden and were not applicable to the synthetic data: it took trendsceek (v.1.0.0) 10 h and BOOST-GP 8 h to analyze even one synthetic data with 1000 genes and 673 locations, which were 24,000 and 17,700 times slower than SPARK-X, respectively. In addition, SOMDE (v.0.1.8) failed to run in approximately 90% of the genes and produced NA values. Therefore, we did not include these three SE methods for comparison.

### Evaluating method performance in identifying cell-cell communications based on simulations

We illustrate the benefits of SRTsim for benchmarking cell-cell communication methods using simulations. We focus on two commonly used cell-cell communication methods for spatial transcriptomics that include Giotto (v1.0.3) and CellphoneDBv3, both examining the expression of ligand-receptor gene pairs to identify pairs that are used for communication between interacting cells from two cell types. We performed simulations to evaluate and compare the performance of these methods.

Specifically, we used the single-cell resolution STARmap dataset to serve as the tissue shape to generate gene expression on the tissue. To do so, we first estimated the shape profile based on the spatial locations of the reference data and randomly generated 5000 locations within the estimated shape profile to serve as the single-cell locations. We assumed that these single cells belong to four different cell types and we created four equal-sized regions on the tissue with distinct cell type composition in each region. In particular, we set the cell types with the highest to lowest proportions to be cell type 1, 2, 3, and 4 in the first spatial region; to be 2, 3, 4, 1 in the second spatial region; to be 3, 4, 1, 2 in the third spatial region; and to be 4, 1, 2, 3 in the fourth spatial region. We then considered four cell-type composition scenarios in the simulations. In the first scenario, each layer contains one dominant cell type, with 70% of the cells belonging to the dominant cell type and 10% of the cells belonging to each of the three minor cell types. In the second scenario, each layer contains two dominant cell types with equal proportions, each consisting of 45% of the cells, along with two minor cell types each consisting of 5% of the cells. In the third scenario, each layer contains two major cell types with unequal proportion, with one consisting of 60% of the cells and the other consisting of 30% of the cells, along with two minor cell types each consisting of 5% of the cells. In the fourth scenario, each layer contains three major cell types and one minor cell type, consisting of 35%, 30%, 30%, or 5% of the cells, respectively. In each scenario, we randomly assigned each single cell in each region to one of the four cell types based on a multinomial distribution with parameters set to be the cell type composition in the region. We performed 10 simulation replicates for each simulation scenario.

In each simulation replicate, we first simulated the expression level of 5000 genes for each single cell in two different ways. In the first way, we simulated homogeneous gene expression by generating expression level for each gene for a total of 8000 cells from a common negative binomial distribution, with parameters inferred based on all cells in the reference data. We then randomly assigned these cells into four cell types, with 2000 cells for each cell type. In the second way, we simulated heterogeneous gene expression by randomly selecting four cell types in the STARmap dataset and then generating the expression level of each gene in 2000 cells for each cell type using a cell type-specific negative binomial distribution, where the distribution parameters are estimated based on the particular cell type in the reference data. In either way, we randomly sampled cells from the 2000 cells in each cell type and assigned them to the spatial locations according to the cell type composition described in the above paragraph.

Next, we increased the expression levels for the ligand-receptor pairs that mediate the cell-cell communications. To do so, we obtained 445 ligands and 471 receptors from CellphoneDBv3. These ligands and receptors correspond to 691 unique genes and form 930 ligand-receptor (L-R) gene pairs. For each L-R pair in turn, we randomly assign it to a pair of cell types (A-B), in order to create the scenario that cell type A communicate with cell type B through the ligand on cell type A and the receptor on cell type B. We denote such interaction as L-R-A-B pair. For four cell types, we obtained a total of 16 combinations of (ordered) cell type pairs, with the number of L-R pairs for each cell type pair ranging from 47 to 77 (median=57) in the simulations. With the allocated L-R-A-B pairs, we increased the gene expression for the L and R genes so that the L-R pair on adjacent A-B cell types are higher than that in the other cell types. In particular, for each cell in turn, we first identified its nearest four cells on the tissue and treat them as adjacent cells. If an A cell is adjacent to a B cell, we then increased the expression level of the L gene in the A cell by a fixed fold, with the fold increase set to be either 1 (null), 5, or 10, representing zero, moderate, or high level of cell-cell communications.

For each simulated spatial transcriptomics dataset, we evaluated the performance of four cell-cell communication methods to detect interacting L-R-A-B pairs on the tissue. These four methods include (1) Giotto with spatial information; (2) Giotto without spatial information; (3) CellphoneDBv3 with spatial information; and (4) CellphoneDBv3 without spatial information. Each method examines one L-R-A-B pair at a time and tests whether the examined pair is significant. Specifically, for Giotto with spatial information, it calculates the mean of two values: the average ligand expression level of cells in cell type A that interact with cell type B; and the average receptor expression level of cells in cell type B that interact with cell type A. Afterwards, it calculates the proportion of the means in the permutations that are higher or lower than the actual mean. For Giotto without spatial information, it calculates the mean of two values: the average ligand expression level of all cells in cell type A and the average receptor expression level of all cells in cell type B. Afterwards, it calculates the proportion of the means in the permutations that are higher or lower than the observed mean. For CellphoneDBv3 with spatial information, it calculates the mean of the average receptor expression level of cell type A and the average ligand expression level of the cell type B. Afterwards, it calculates the proportion of the means in the permutations that are as high or higher than the actual mean, considering only combinations of cell type pairs that co-exist in a spatial domain. For CellphoneDBv3 without spatial information, it calculates the mean of the average receptor expression level of cell type A and the average ligand expression level of the cell type B. Afterwards, it calculates the proportion of the means in the permutations that are as high or higher than the actual mean, considering all combinations of cell type pairs.

We evaluated the performances of different methods in detecting the L-R-A-B pairs based on the F1 score, which is the harmonic mean of precision and recall, at different adjusted *p*-value thresholds of 0.001, 0.005, 0.01, 0.025, 0.05, or 0.1 [81]. While we primarily focused on using F1 score, which is the most commonly used metric in the literature of cell-cell communications [81–83], we also examined other metrics including precision (TP/TP+FP), recall (TP/TP+FN), and specificity (TN/TN+FP).

## Supplementary Information

**Additional file 1: Fig. S1.** An overview of the Shiny-app component of SRTsim for reference-free simulations. **Fig. S2.** Benchmarking SRTsim against eight existing scRNA-seq simulators for generating SRT data based on seven SRT data by 10x Visium. **Fig. S3.** Benchmarking SRTsim against eight existing scRNA-seq simulators for generating SRT data based on eight DLPFC SRT data by 10x Visium. **Fig. S4.** Benchmarking SRTsim against eight existing scRNA-seq simulators for generating SRT data based on six SRT data by DBiT-seq and one SRT data by ST. **Fig. S5.** Benchmarking SRTsim against eight existing scRNA-seq simulators for generating SRT data based on SRT data by single-cell resolution SRT techniques. **Fig. S6.** Scatter plots comparing Moran’s I in the synthetic data generated by different simulators versus that in the reference data. **Fig. S7.** Benchmarking SRTsim against scDesign2 and SymSim for generating SRT data with a different number of locations. **Fig. S8.** Computational cost of different simulators for generating synthetic data based on references from different spatial transcriptomics techniques. **Fig. S9.** Distributions of six metrics and Moran’s I for the simulation based on the reference DLPFC sample 151673. **Fig. S10.** Spatial distributions of the domain assignments by six spatial clustering methods on the synthetic data generated by SRTsim in the tissue-based simulations. **Fig. S11.** Spatial distributions of domain assignments by six spatial clustering methods on the synthetic data generated by SRTsim in the domain-specific simulations. **Fig. S12.** Distributions of six metrics for the simulations based on STARmap data. **Fig. S13.** Spatial distributions of domain assignments by six spatial clustering methods based on the synthetic data generated by SRTsim via the tissue-based simulation. **Fig. S14.** Spatial distributions of domain assignments by six spatial clustering methods based on the synthetic data generated by SRTsim via the domain-specific simulation. **Fig. S15.** The performance of stLearn with Louvain measured by the adjusted Rand index is evaluated under simulations with varying-sequencing-depth (y-axis) and varying-location-number (x-axis). **Fig. S16.** Comparisons between the reference and synthetic data generated by SRTsim. **Fig. S17.** Benchmarking spatial expression analysis methods using synthetic data from SRTsim based on the DLPFC reference. **Fig. S18.** Benchmarking spatial expression analysis methods using synthetic data from SRTsim based on the STARmap reference. **Fig. S19.** Benchmarking cell-cell communication identification methods using synthetic data from SRTsim (precision). **Fig. S20.** Benchmarking cell-cell communication identification methods using synthetic data from SRTsim (recall rate). **Fig. S21.** Benchmarking cell-cell communication identification methods using synthetic data from SRTsim (specificity). **Table S1.** Summary of 49 SRT datasets used in the present study. **Table S5.** Computational stability of all simulators. X represents failure, while – represents success. **Table S6.** Total number of alternatives in SE analysis.

**Additional file 2: Supplementary Table S2.** *P*-values from the Kolmogorov–Smirnov (KS) test for the DFPLC 151673 sample. The KS test is performed for each metrics between the synthetic data and reference data.

**Additional file 3: Supplementary Table S3.** *P*-values from the Kolmogorov–Smirnov (KS) test for the Lung12 FOV1 sample. The KS test is performed for each metrics between the synthetic data and reference data.

**Additional file 4: Supplementary Table S4.** Number of significance (*P*-value < 0.05) in the Kolmogorov–Smirnov (KS) test across ten replicates for all the data (n=29). Datasets with at least one failure in selected simulators were excluded. The KS test is performed for each metrics between the synthetic data and reference data.

**Additional file 5.** Review history.

## Data Availability

All codes, processed data, and analysis results in this paper are publicly available at GitHub [84] and Zenodo [85]. The source code is released under the GPL-3.0 license. The R package SRTsim is also available at CRAN. The 10x Visium data are available at https://www.10xgenomics.com/resources/datasets. The DFPLC data generated with 10x Visium are available at http://spatial.libd.org/spatialLIBD/. The ST data are available at https://www.spatialresearch.org/resources-published-datasets/doi-10-1126science-aaf2403/. The DBiT-seq data are available at https://www.ncbi.nlm.nih.gov/geo/query/acc.cgi?acc=GSE137986. The seqFISH+ data are available at https://github.com/CaiGroup/seqFISH-PLUS. The STARmap data are available at https://xzhoulab.github.io/SRTsim/.The Slide-seqV2 data are available at the Broad institute’s single-cell repository https://singlecell.broadinstitute.org/single_cell/study/SCP815. The CosMx SMI data are available at https://nanostring.com/products/cosmx-spatial-molecular-imager/ffpe-dataset/. The MERFISH data are available at https://vizgen.com/data-release-program/.
