# Supplementary material for: SRTsim: spatial pattern preserving simulations for spatially resolved transcriptomics
Source: Genome Biol. 2023 Mar 3;24:39. doi: 10.1186/s13059-023-02879-z (PMC9983268; doi:10.1186/s13059-023-02879-z)
Supplement: Supplementary file 1 — Additional file 1: Fig. S1. An overview of the Shiny-app component of SRTsim for reference-free simulations. Fig. S2. Benchmarking SRTsim against eight existing scRNA-seq simulators for generating SRT data based on seven SRT data by 10x Visium. Fig. S3. Benchmarking SRTsim against eight existing scRNA-seq simulators for generating SRT data based on eight DLPFC SRT data by 10x Visium. Fig. S4. Benchmarking SRTsim against eight existing scRNA-seq simulators for generating SRT data based on six SRT data by DBiT-seq and one SRT data by ST. Fig. S5. Benchmarking SRTsim against eight existing scRNA-seq simulators for generating SRT data based on SRT data by single-cell resolution SRT techniques. Fig. S6. Scatter plots comparing Moran’s I in the synthetic data generated by different simulators versus that in the reference data. Fig. S7. Benchmarking SRTsim against scDesign2 and SymSim for generating SRT data with a different number of locations. Fig. S8. Computational cost of different simulators for generating synthetic data based on references from different spatial transcriptomics techniques. Fig. S9. Distributions of six metrics and Moran’s I for the simulation based on the reference DLPFC sample 151673. Fig. S10. Spatial distributions of the domain assignments by six spatial clustering methods on the synthetic data generated by SRTsim in the tissue-based simulations. Fig. S11. Spatial distributions of domain assignments by six spatial clustering methods on the synthetic data generated by SRTsim in the domain-specific simulations. Fig. S12. Distributions of six metrics for the simulations based on STARmap data. Fig. S13. Spatial distributions of domain assignments by six spatial clustering methods based on the synthetic data generated by SRTsim via the tissue-based simulation. Fig. S14. Spatial distributions of domain assignments by six spatial clustering methods based on the synthetic data generated by SRTsim via the domain-specific simulation. Fig. S15. The performan [file 13059_2023_2879_MOESM1_ESM.docx]

**Supplementary Figures**


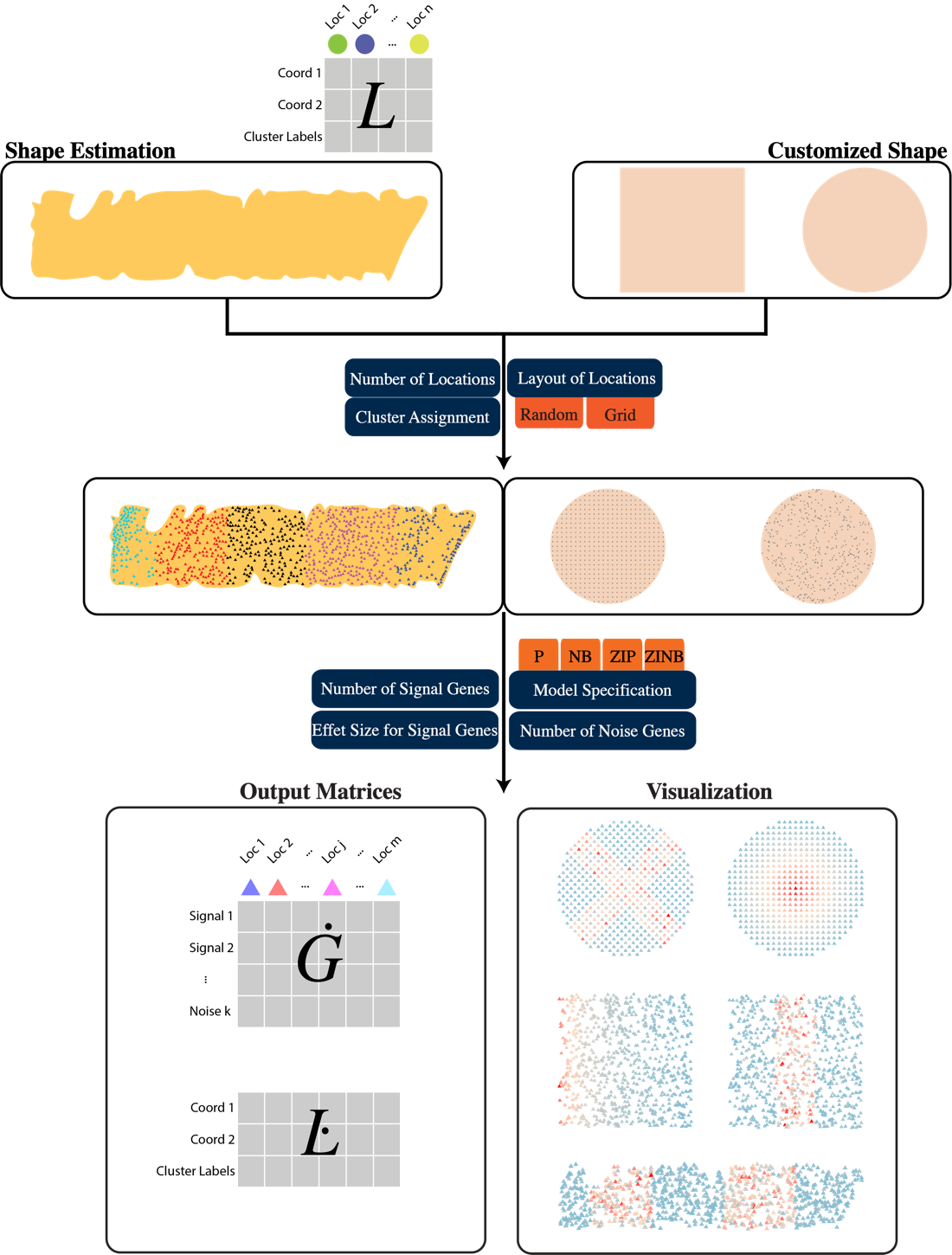


**Fig. S1 An overview of the Shiny-app component of SRTsim for reference-free simulations.** In reference-free simulations, SRTsim allows users to either select from a predefined set of shapes or use a customized shape to serve as the tissue shape. Afterwards, SRTsim creates locations on the tissue based on a user-specified location number and further creates domain-specific spatial expression patterns by a set of user-specified parameters. SRTsim then outputs the generated synthetic data in the form of an expression matrix and a location matrix. SRTsim perform all these steps through a shiny interface, facilitating data visualization.


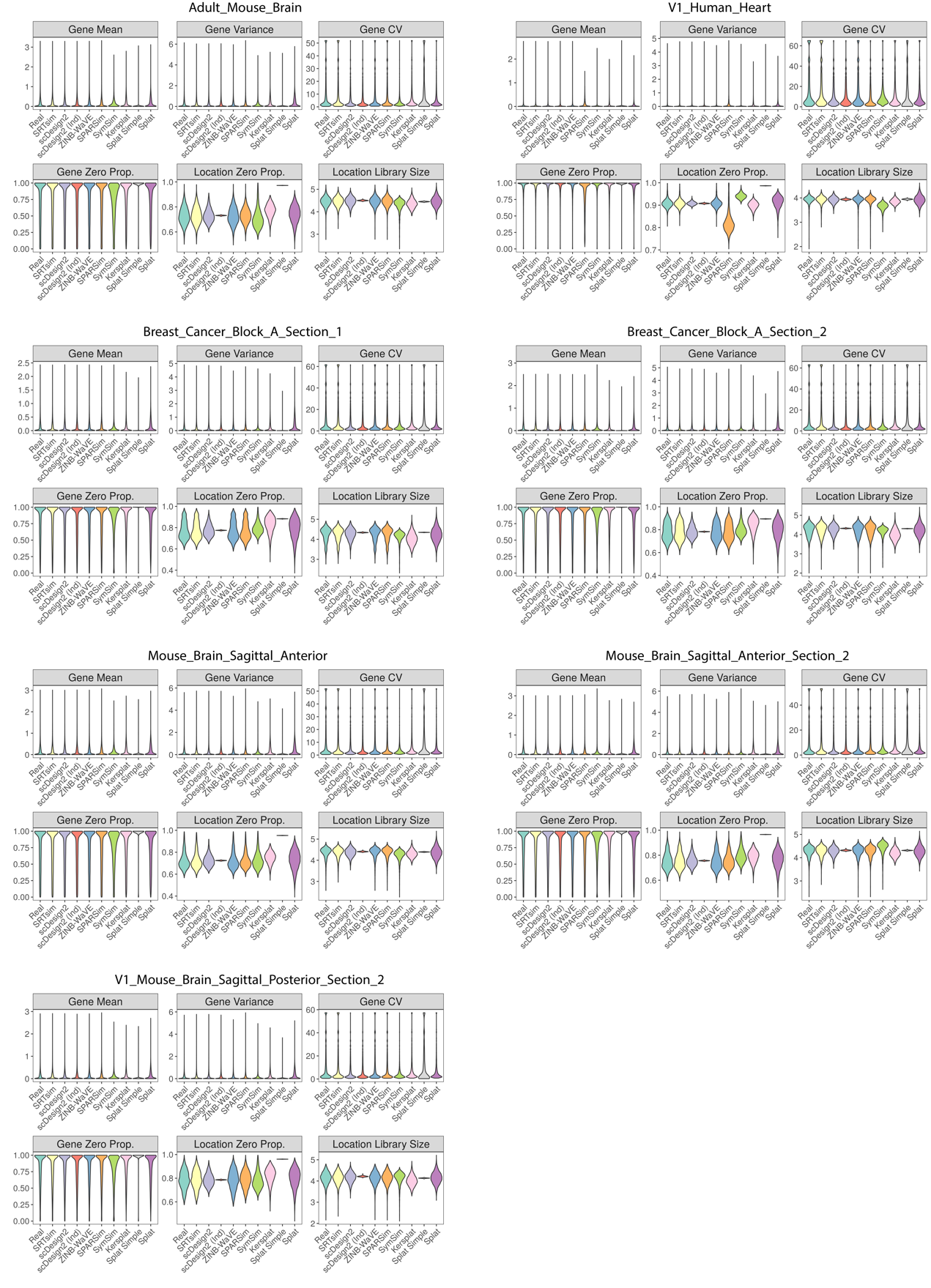


**Fig. S2 Benchmarking SRTsim against eight existing scRNA-seq simulators for generating SRT data based on seven SRT data by 10x Visium.** Violin plots show the distributions of six metrics (six panels) in the reference data (real; x-axis) and in the synthetic data generated by different simulators (x-axis). The six metrics include four gene-wise metrics (expression mean; variance; coefficient of variation, cv; and zero proportion) and two location-wise metrics (zero proportion and library size). The examined simulators include SRTsim, scDesign2, scDesign2 without copula (scDesign2 (ind)), ZINB-WaVE, SPARSim, SymSim, and three variants of the splatter package (kersplat, splat simple, and splat).


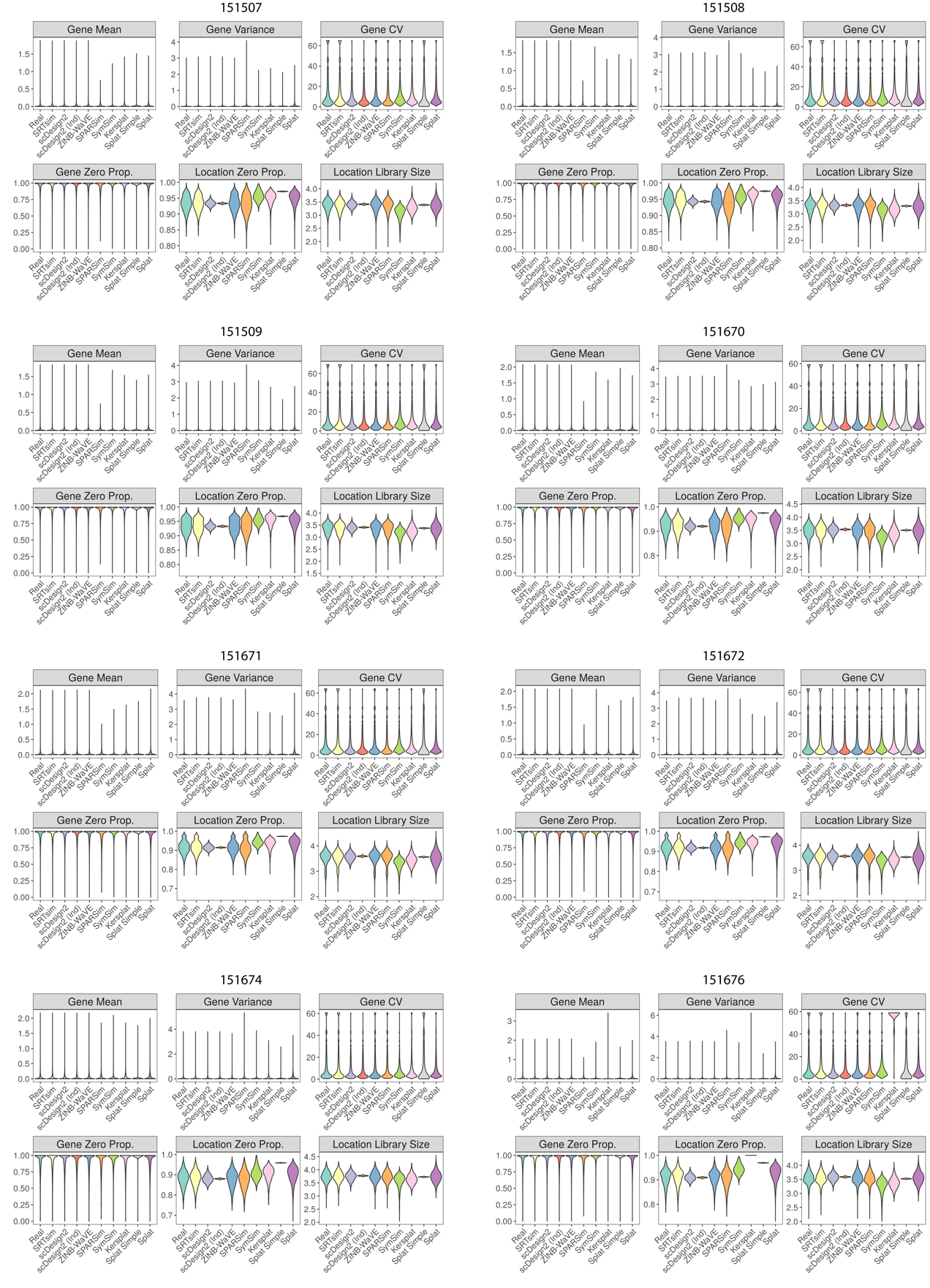


**Fig. S3 Benchmarking SRTsim against eight existing scRNA-seq simulators for generating SRT data based on eight DLPFC SRT data by 10x Visium.** Violin plots show the distributions of six metrics (six panels) in the reference data (real; x-axis) and in the synthetic data generated by different simulators (x-axis). The six metrics include four gene-wise metrics (expression mean; variance; coefficient of variation, cv; and zero proportion) and two location-wise metrics (zero proportion and library size). The examined simulators include SRTsim, scDesign2, scDesign2 without copula (scDesign2 (ind)), ZINB-WaVE, SPARSim, SymSim, and three variants of the splatter package (kersplat, splat simple, and splat).


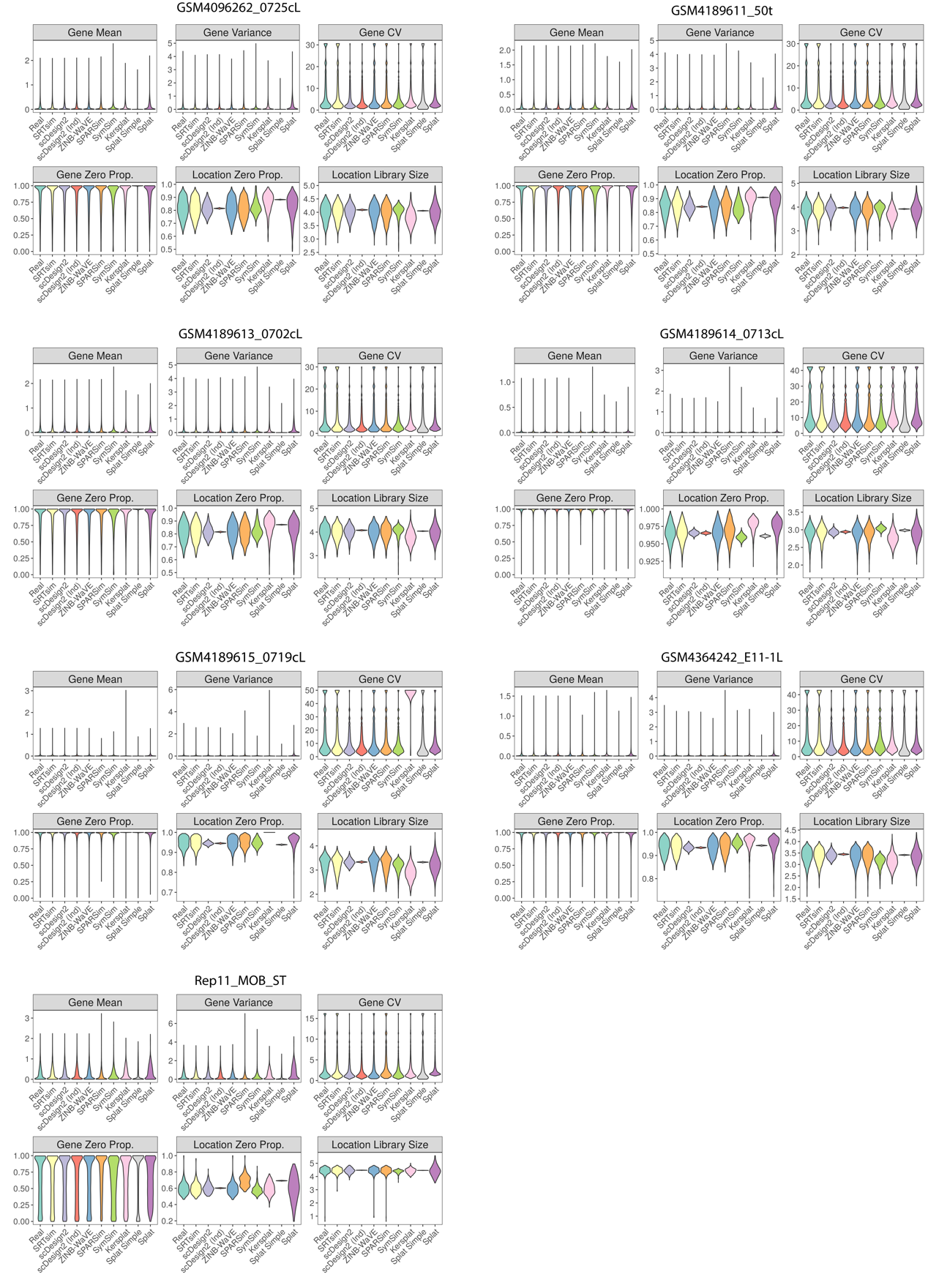


**Fig. S4 Benchmarking SRTsim against eight existing scRNA-seq simulators for generating SRT data based on six SRT data by DBiT-seq and one SRT data by ST.** Violin plots show the distributions of six metrics (six panels) in the reference data (real; x-axis) and in the synthetic data generated by different simulators (x-axis). The six metrics include four gene-wise metrics (expression mean; variance; coefficient of variation, cv; and zero proportion) and two location-wise metrics (zero proportion and library size). The examined simulators include SRTsim, scDesign2, scDesign2 without copula (scDesign2 (ind)), ZINB-WaVE, SPARSim, SymSim, and three variants of the splatter package (kersplat, splat simple, and splat).


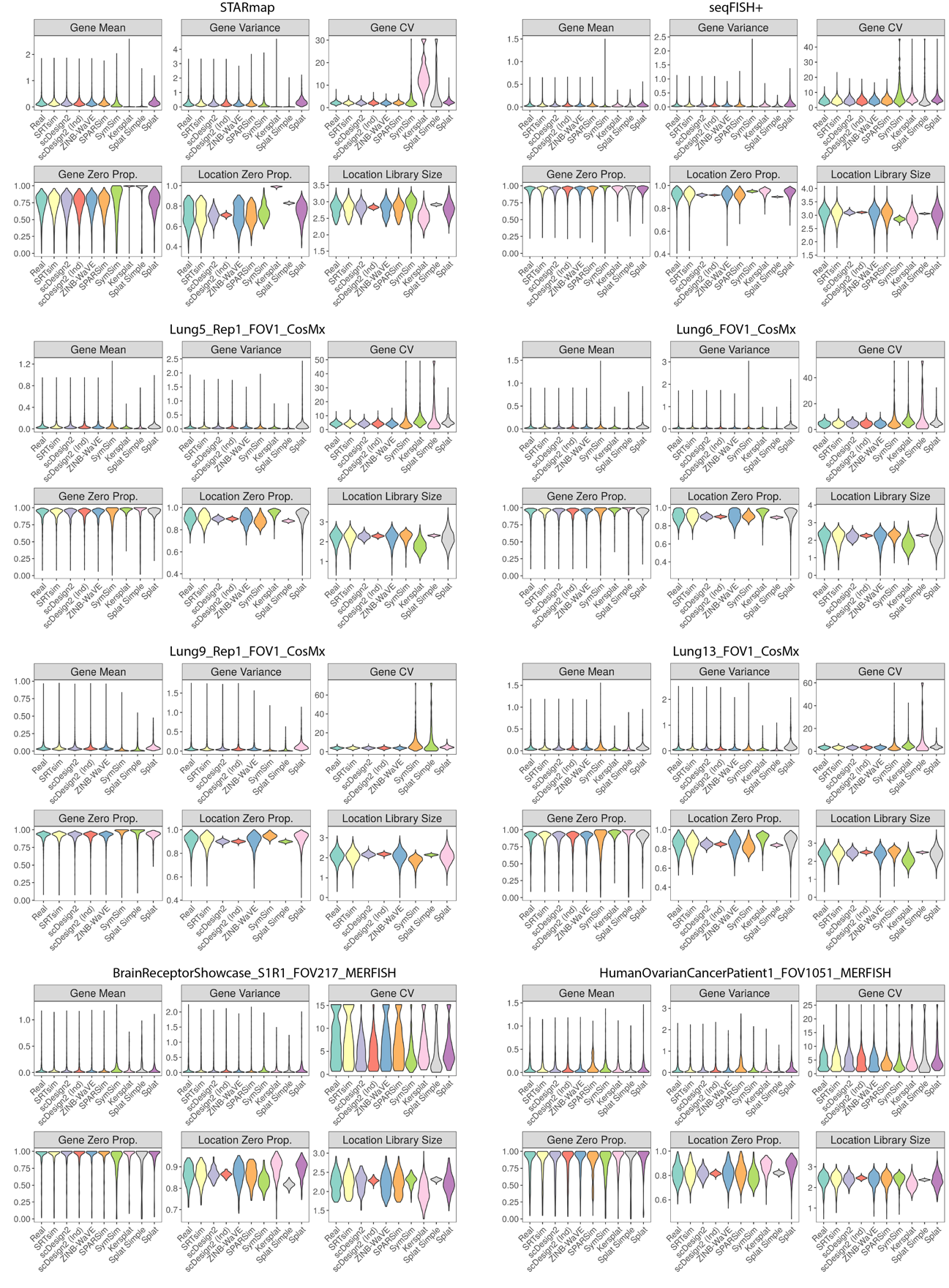


**Fig. S5 Benchmarking SRTsim against eight existing scRNA-seq simulators for generating SRT data based on SRT data by single-cell resolution SRT techniques.** Violin plots show the distributions of six metrics (six panels) in the reference data (real; x-axis) and in the synthetic data generated by different simulators (x-axis). The six metrics include four gene-wise metrics (expression mean; variance; coefficient of variation, cv; and zero proportion) and two location-wise metrics (zero proportion and library size). The examined simulators include SRTsim, scDesign2, scDesign2 without copula (scDesign2 (ind)), ZINB-WaVE, SPARSim, SymSim, and three variants of the splatter package (kersplat, splat simple, and splat).

**
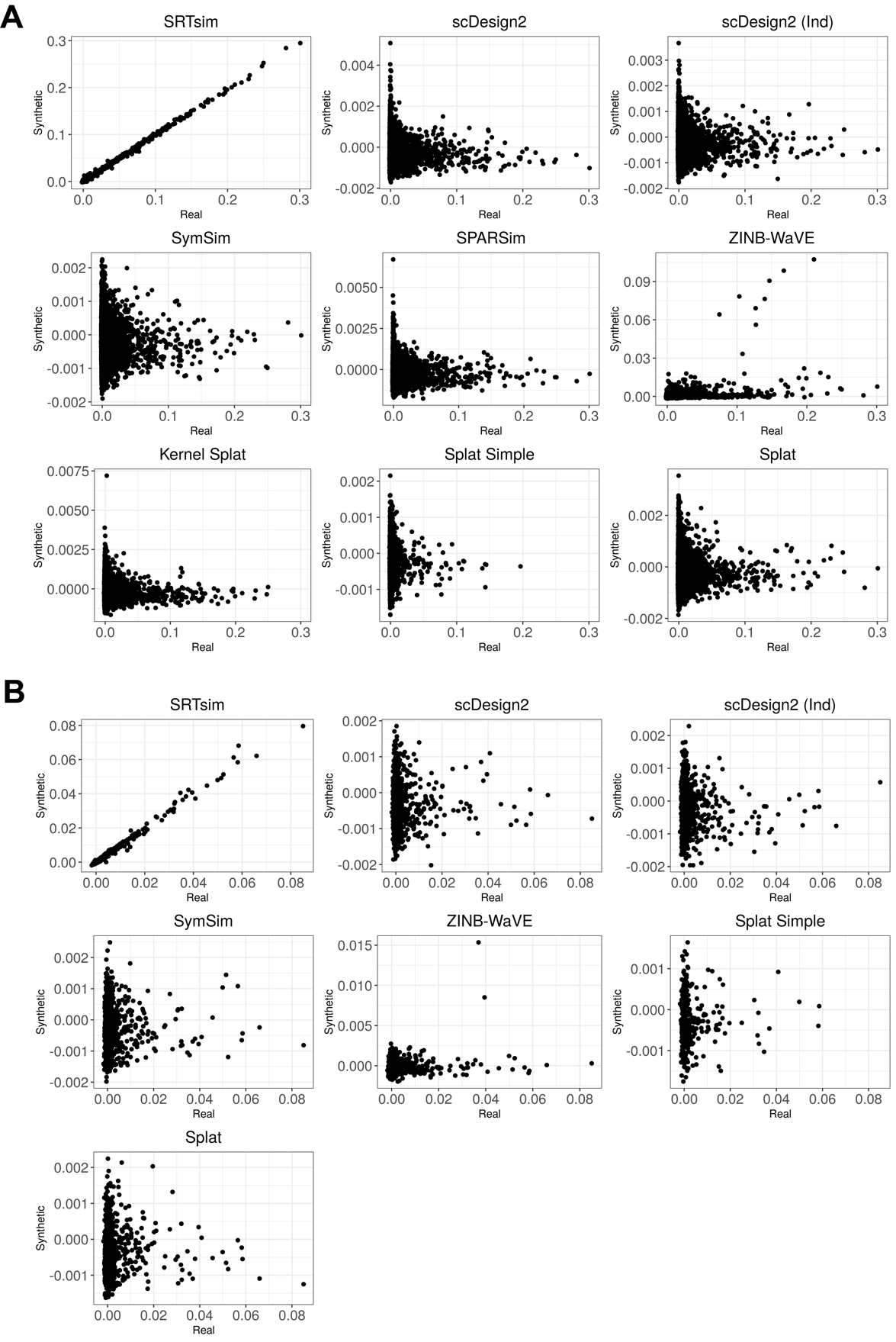
**

**Fig. S6 Scatter plots comparing Moran’s I in the synthetic data generated by different simulators versus that in the reference data. (A)** Sample 151673 in the DLPFC data is used to serve as the reference. **(B)** Sample Lung12 FOV1 in the NSCLC data is used to serve as the reference. SPARsim and kersplat failed to generate synthetic data for NSCLC sample.


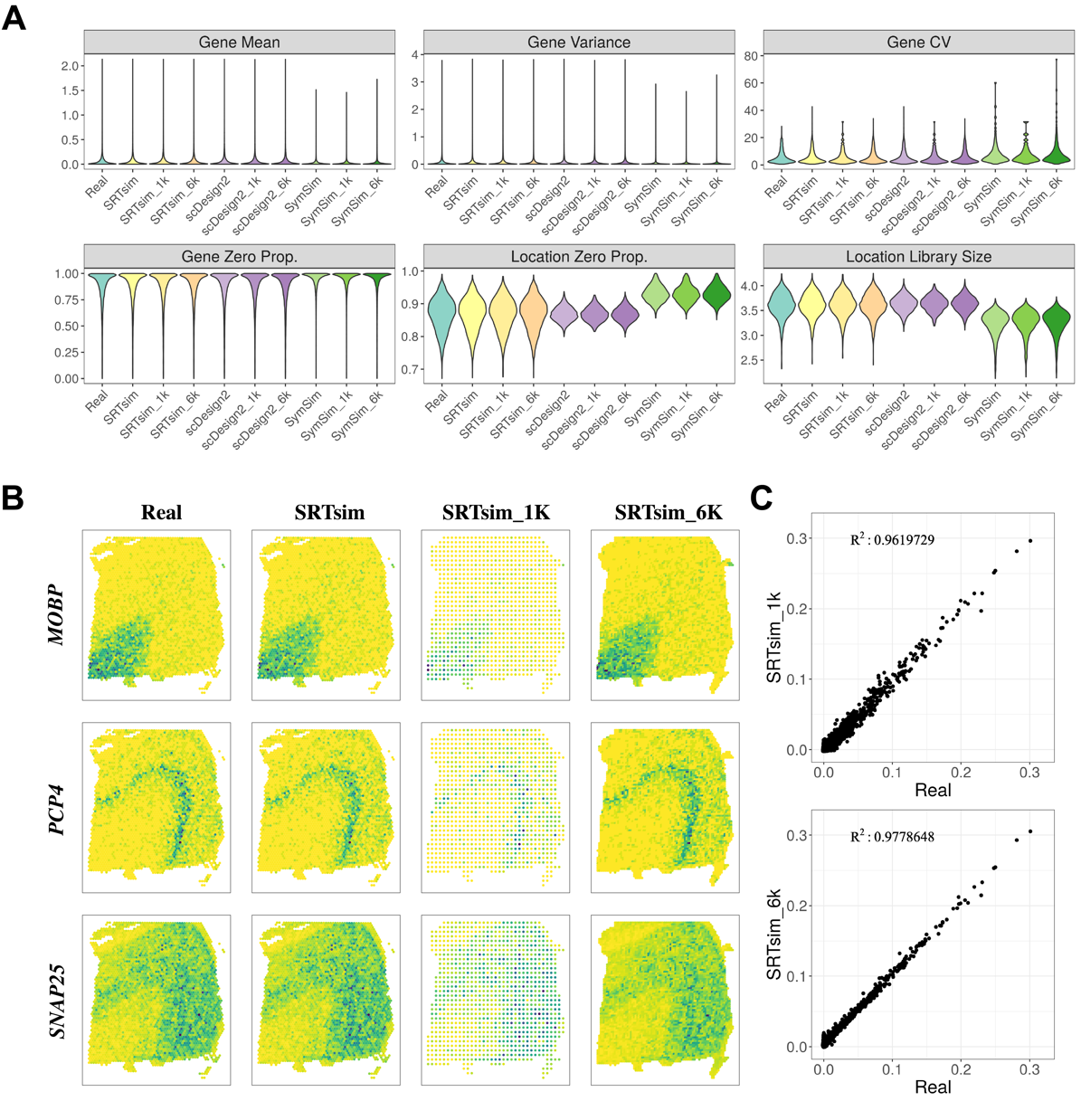


**Fig. S7 Benchmarking SRTsim against scDesign2 and SymSim for generating SRT data with a different number of locations. (A)** Violin plots show the distributions of six metrics (six panels) in the reference data (real; x-axis) and in the synthetic data with a different number of locations generated by different simulators (x-axis). The six metrics include four gene-wise metrics (expression mean; variance; coefficient of variation, cv; and zero proportion) and two location-wise metrics (zero proportion and library size). Genes expressed in less than ten spots in reference data are removed. **(B)** The spatial expression patterns of three representative genes (*MOBP, PCP4, SNAP25*) are displayed in the reference data and in the synthetic data with different sample sizes generated by SRTsim. **(C)** Scatter plots of Moran’s I of reference data and synthetic data with different sample sizes.


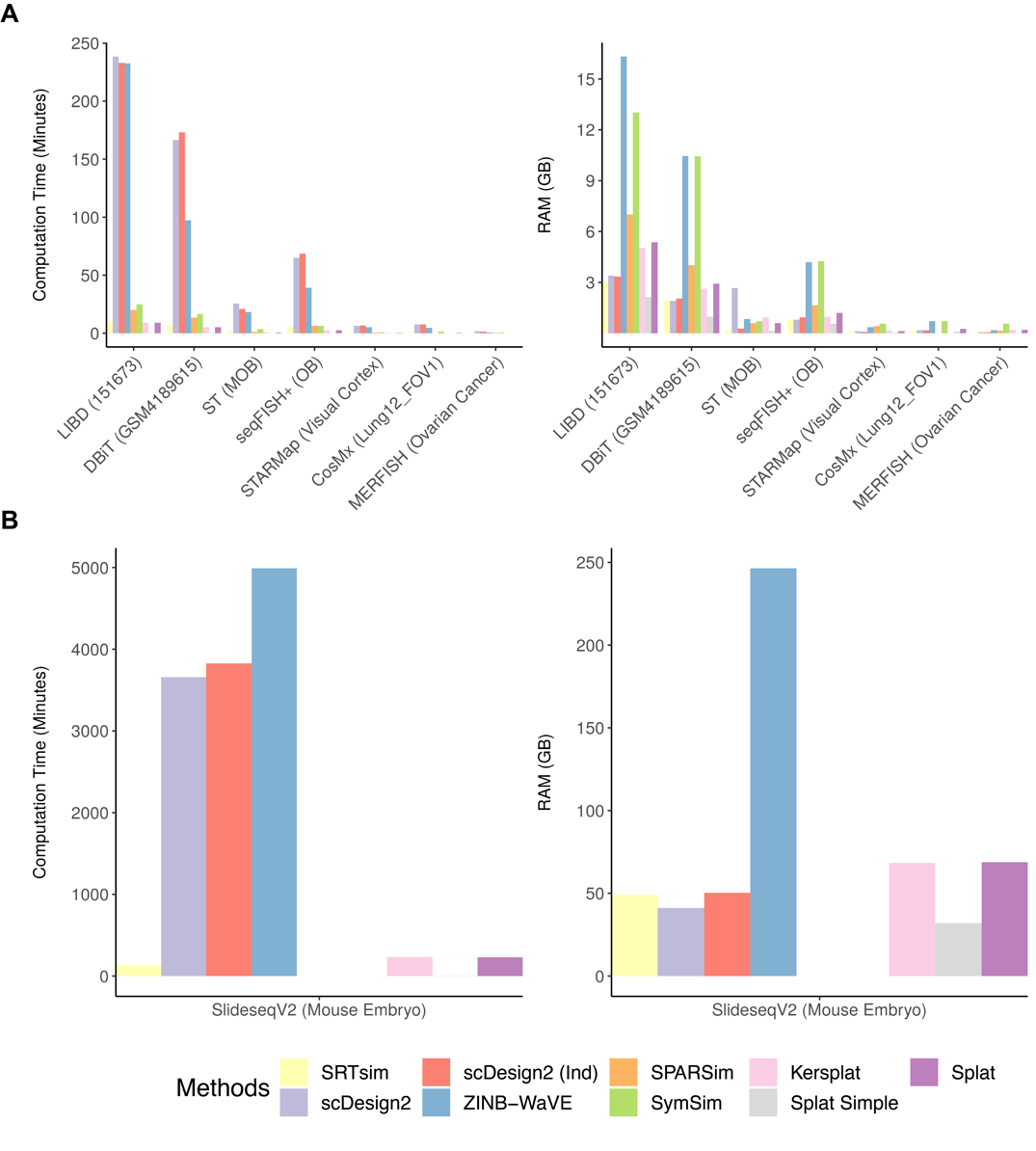


**Fig. S8 Computational cost of different simulators for generating synthetic data based on references from different spatial transcriptomics techniques.** We selected one representative data from each of the seven spatial transcriptomics techniques. (A) Computational time and memory usage for data sets collected through techniques 10x, DBiT, ST, seqFISH+, STARmap, CosMx SMI and MERFISH. SPARsim and kersplat failed to generate synthetic data for CosMx SMI. (B) Computational time and memory usage for a data set collected through SlideseqV2, which is much larger in scale than the other six techniques. SymSim and SPARsim failed to generate synthetic data for SlideseqV2. The examined simulators include SRTsim, scDesign2, scDesign2 without copula (scDesign2 (ind)), ZINB-WaVE, SPARSim, SymSim, and three variants of the splatter package (kersplat, splat simple, and splat).


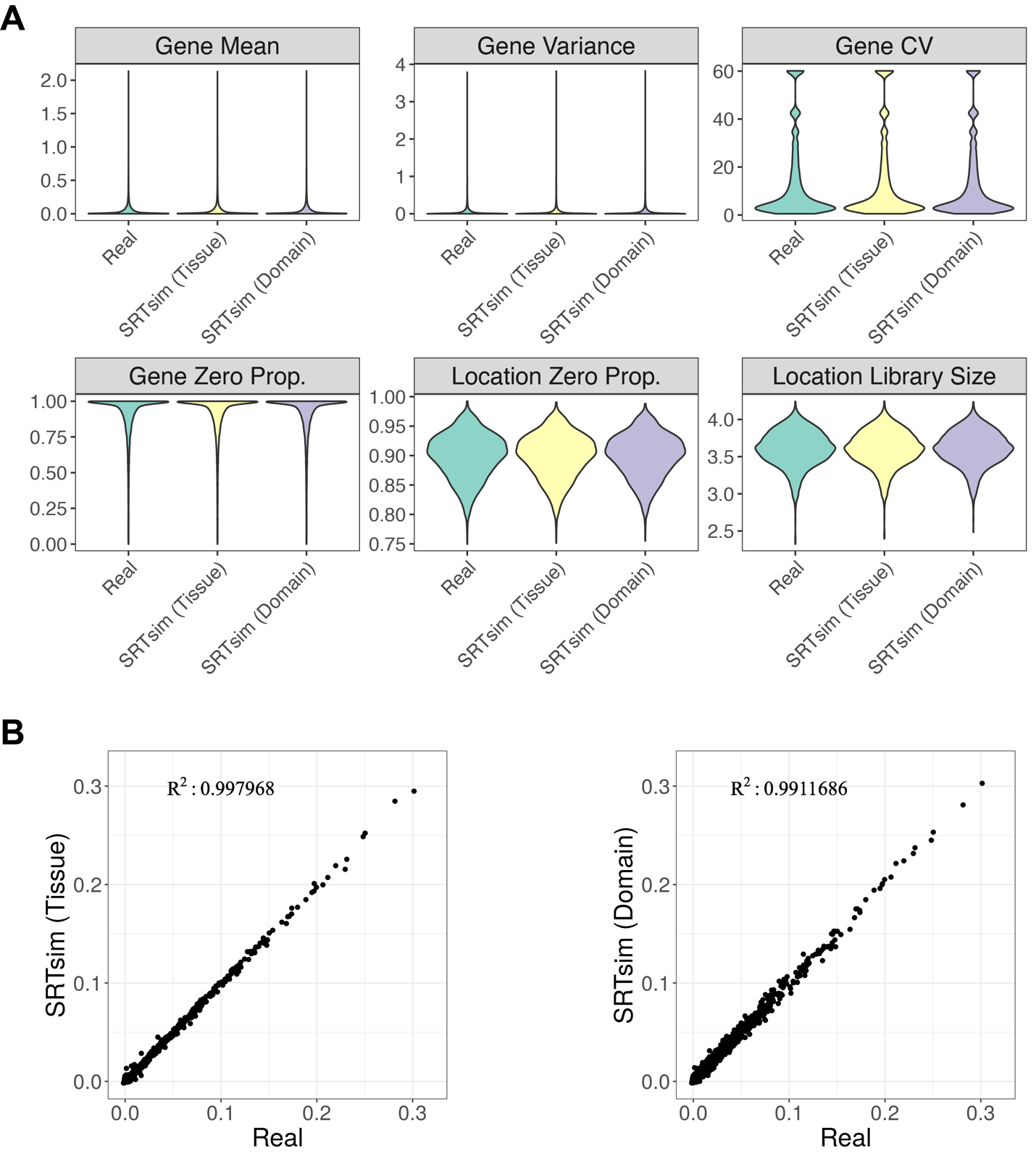
 **Fig. S9 Distributions of six metrics and Moran’s I for the simulation based on the** **reference DLPFC sample 151673.** **(A)** Violin plots show the distributions of six metrics (six panels) in the reference data (real; x-axis) and in the synthetic data generated by SRTsim via either the tissue-based simulations or the domain-specific simulations (x-axis). The six metrics include four gene-wise metrics (expression mean; variance; coefficient of variation, cv; and zero proportion) and two location-wise metrics (zero proportion and library size). **(B)** Scatter plots of Moran’s I in the synthetic data generated by SRTsim (y-axis) via either the tissue-based simulations (left panel) or the domain-specific simulations (right panel) versus that in the reference data (x-axis).


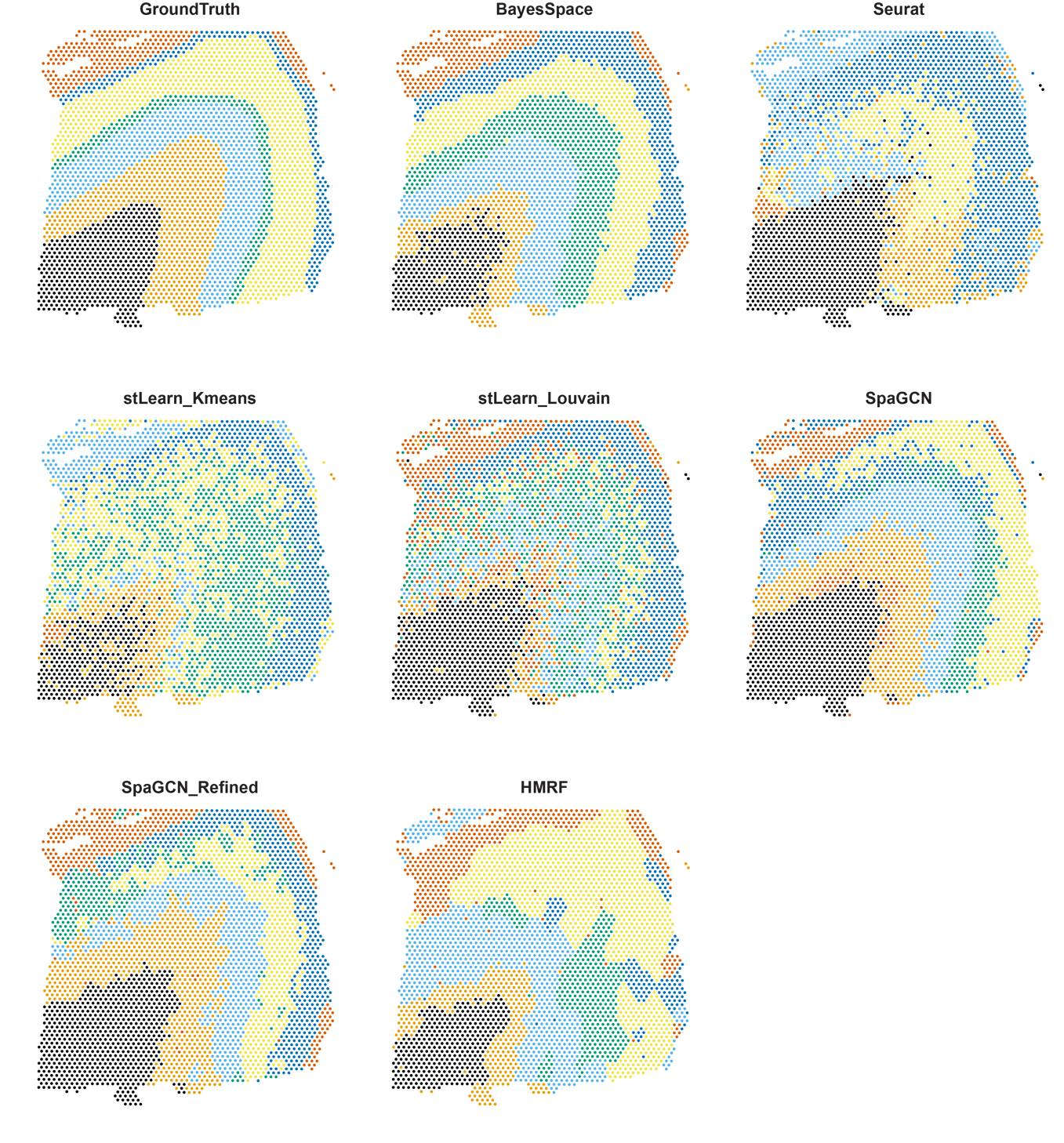


**Fig. S10 Spatial distributions of the ﻿domain assignments by six spatial clustering methods on the synthetic data generated by SRTsim in the tissue-based simulations**. Sample 151673 in the DLPFC data is used as the reference. For each method, the simulation replicate with the median ARI is presented.

**
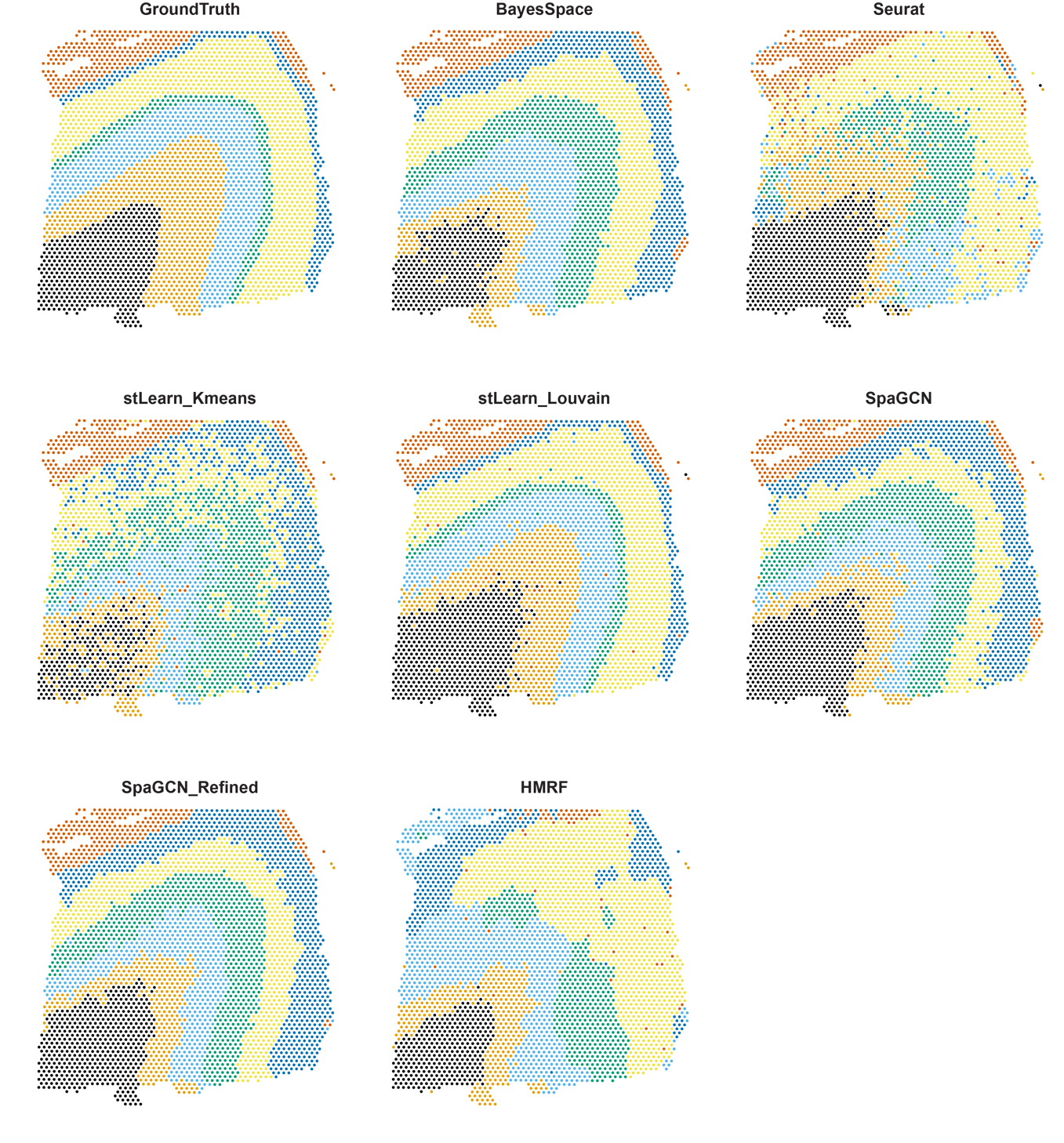
**

**Fig. S11 Spatial distributions of ﻿domain assignments by six spatial clustering methods on the synthetic data generated by SRTsim in the domain-specific simulations**. Sample 151673 in the DLPFC data is used as the reference. For each method, the simulation replicate with the median ARI is presented.

**Fig. S12 Distributions of six metrics for the simulations based on STARmap data**. Violin plots show the distributions of six metrics (six panels) in the reference data (real; x-axis) and in the synthetic data generated by SRTsim via either the tissue-based simulations or the domain-specific simulations (x-axis). The six metrics include four gene-wise metrics (expression mean; variance; coefficient of variation, cv; and zero proportion) and two location-wise metrics (zero proportion and library size).


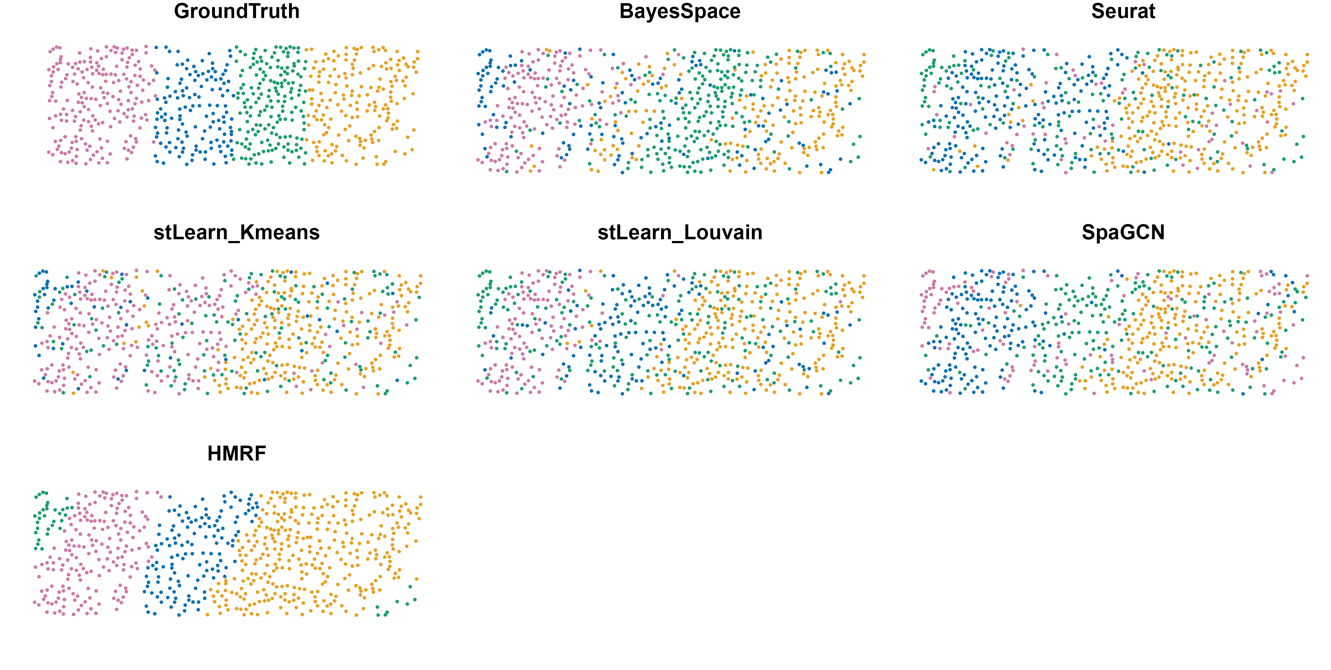


**Fig. S13 Spatial distributions of ﻿domain assignments by six spatial clustering methods based on the synthetic data generated by SRTsim via the tissue-based simulation**. The STARmap data is used to serve as the reference. For each method, the simulation replicate with the median ARI is presented.


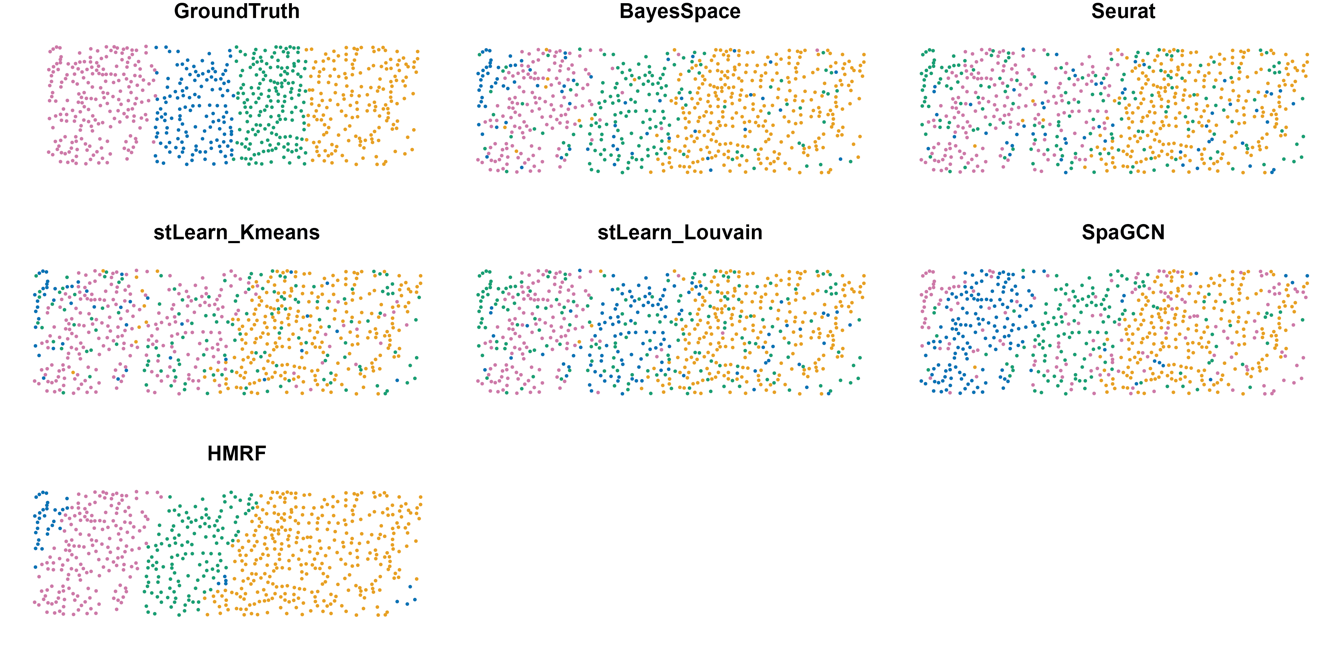


**Fig. S14 ﻿ Spatial distributions of ﻿domain assignments by six spatial clustering methods based on the synthetic data generated by SRTsim via the domain-specific simulation**. The STARmap data is used to serve as the reference. For each method, the simulation replicate with the median ARI is presented.

**
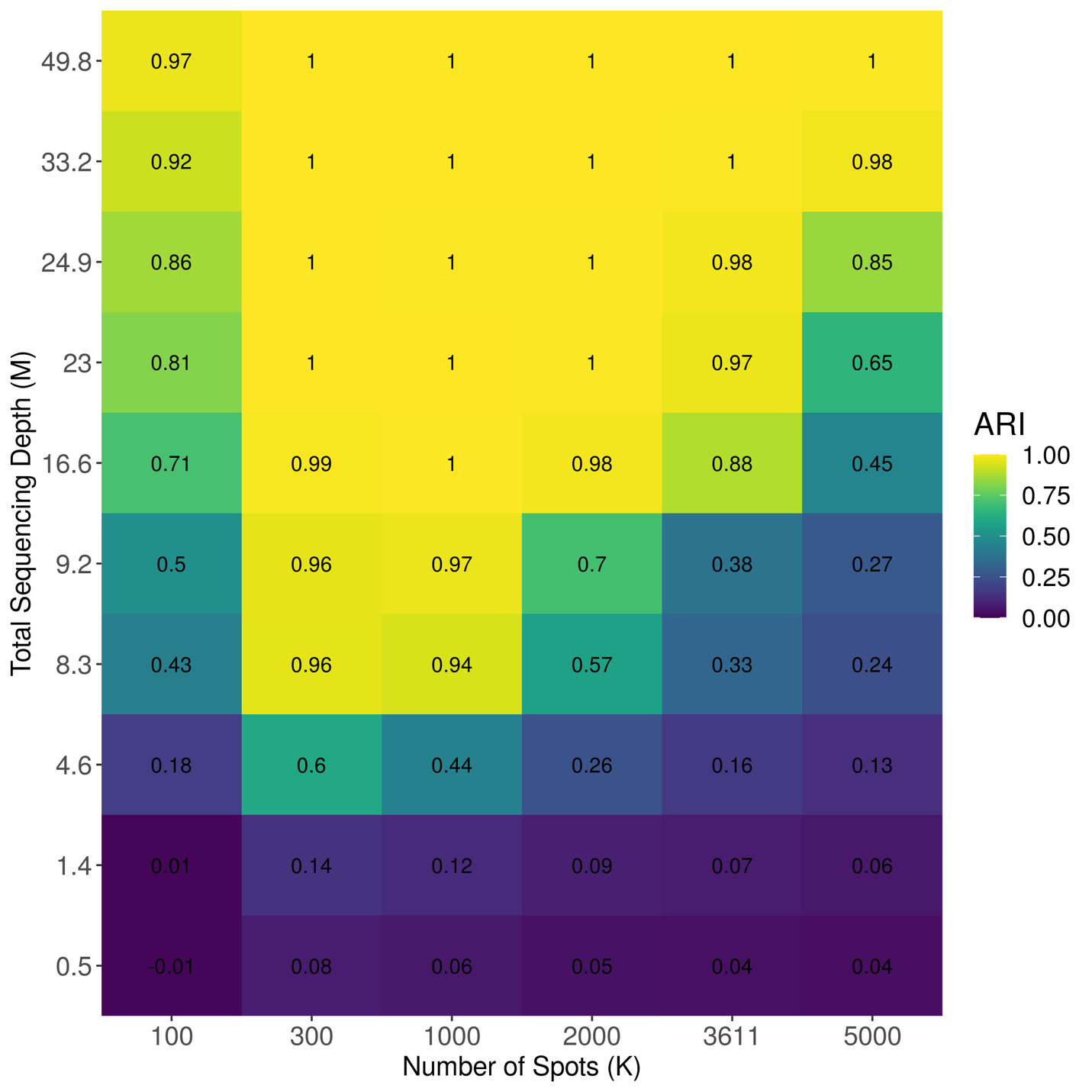
**

**Fig. S15 The performance of stLearn with Louvain measured by the adjusted Rand index is evaluated under simulations with varying-sequencing-depth (y-axis) and varying-location-number (x-axis).** Sample 151673 in the DLPFC data is used to serve as the reference for simulation by SRTsim.


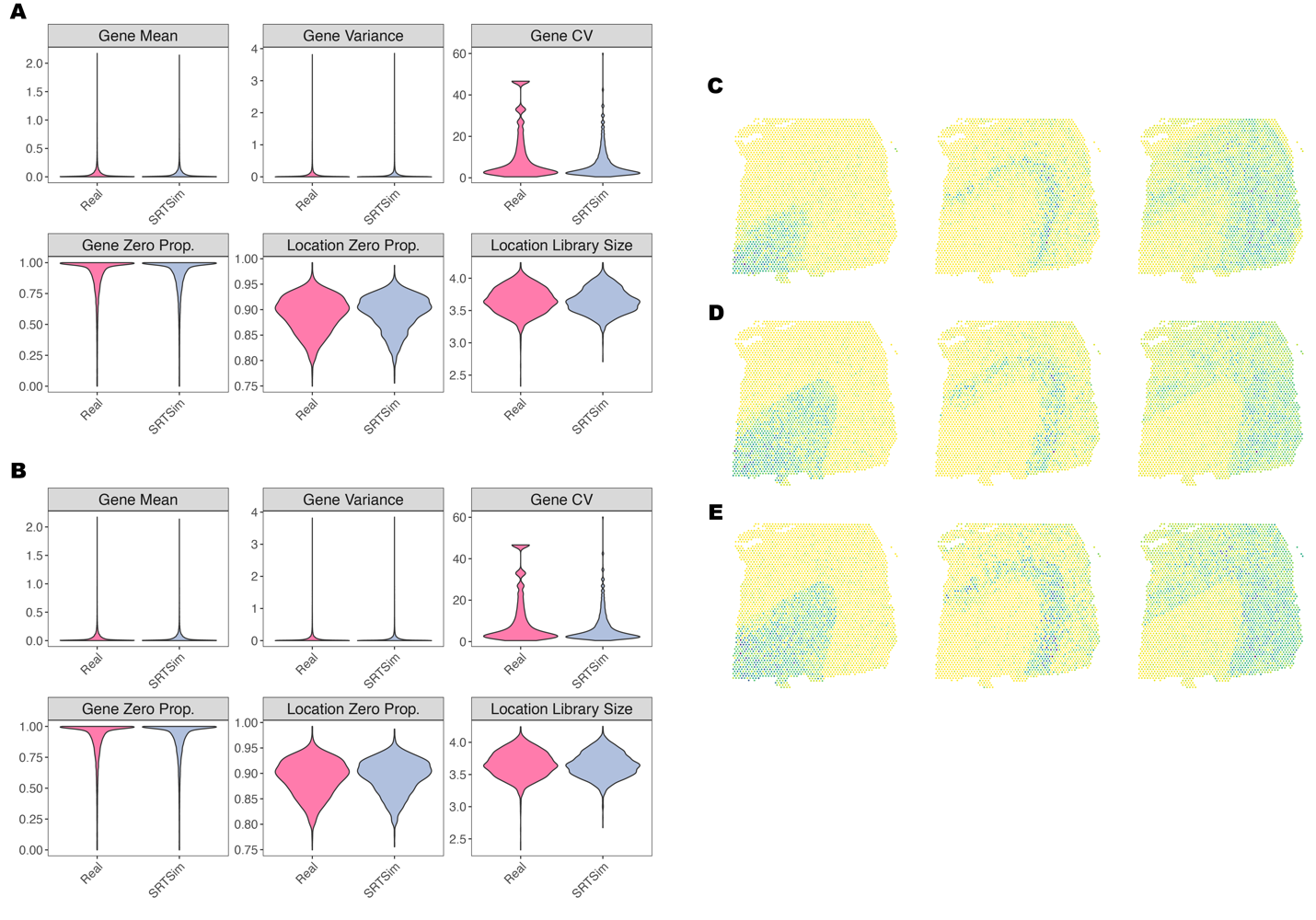


**Fig. S16. Comparisons between the reference and synthetic data generated by SRTsim.** **(A-B)** Violin plots show the distributions of six metrics (six panels) in the reference data (real; x-axis) and in the synthetic data generated by SRTsim (x-axis). The six metrics include four gene-wise metrics (expression mean; variance; coefficient of variation, cv; and zero proportion) and two location-wise metrics (zero proportion and library size). **A:** Distribution of six metrics in the five datasets where SpaGCN achieves high ARI values. **B**: Distribution of six metrics in the five datasets where SpaGCN achieves low ARI values. **(C-E)** The spatial expression patterns of three representative genes (*MOBP*, *PCP4*, *SNAP25*) are displayed in the reference data (**C**), one representative synthetic dataset used in **A** (**D**), and one representative synthetic dataset used in **B** (**E**). The first five datasets are not distinguishable from the second five datasets in terms of the distribution of the six metrics we used (**A** vs. **B**). However, some of the marker genes in the second five datasets in **E** appears to show slightly more similarity in their expression between layers as compared to the first five datasets in **D**. For example, *SNAP25* shows slightly more similar expression pattern between the out-layers in the second five datasets as compared to the first datasets. This observation suggests that SpaGCN could achieve high clustering performance when layers are very distinguished from each other but may suffer when the pattern between layers become less apparent.


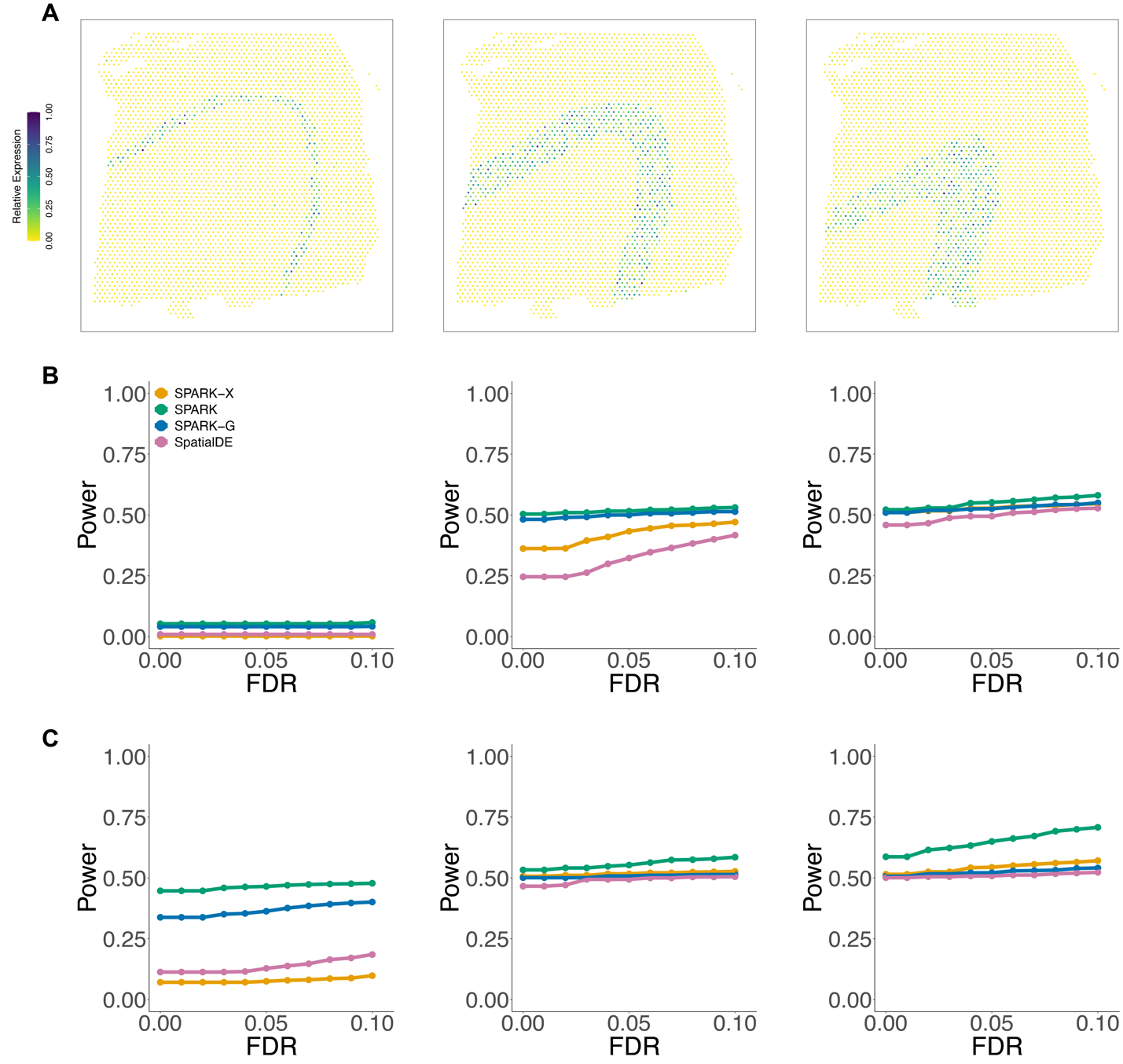


**Fig.S17 Benchmarking spatial expression analysis methods using synthetic data from SRTsim based on the DLPFC reference.** Sample 151673 in the DLPFC data is used to serve as the reference for simulation by SRTsim. **(A)** Representative genes in the DLPFC reference data display three distinct spatial expression patterns corresponding to three different layered structures (Layer4, Layer 5, and Layer 6, respectively). **(B)** Power plots show the proportion of true positives (y-axis) detected by different methods at a range of FDRs (x-axis) for the alternative SRT simulations. SE strength is set to be *five-fold*. **(C)** Power plots show the proportion of true positives (y-axis) detected by different methods at a range of FDRs (x-axis) for the alternative SRT simulations. SE strength is set to be *ten-fold*.


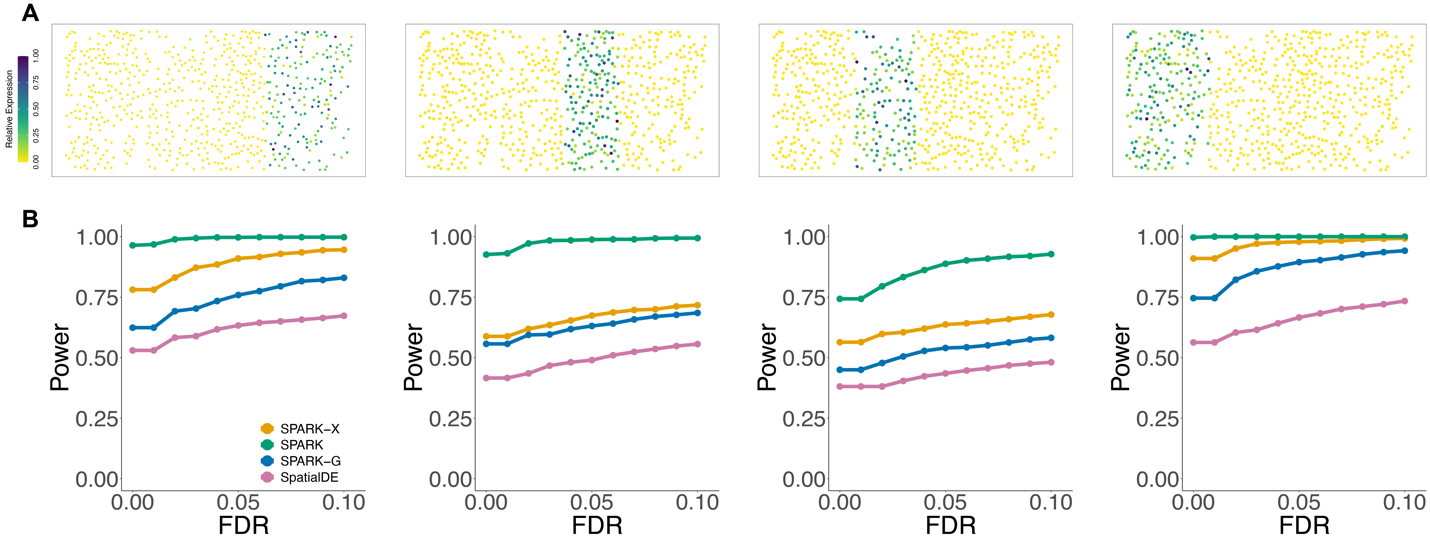


**Fig.S18 Benchmarking spatial expression analysis methods using synthetic data from SRTsim based on the STARmap reference. (A)** Representative genes in the STARmap reference data display four distinct spatial expression patterns that correspond to four different layered structures (Layer2/3, Layer 4, Layer 5, and Layer 6, respectively). **(B)** Power plots show the proportion of true positives (y-axis) detected by different methods at a range of FDRs (x-axis) for the alternative SRT simulations. SE strength is set to be *four-fold*.

**
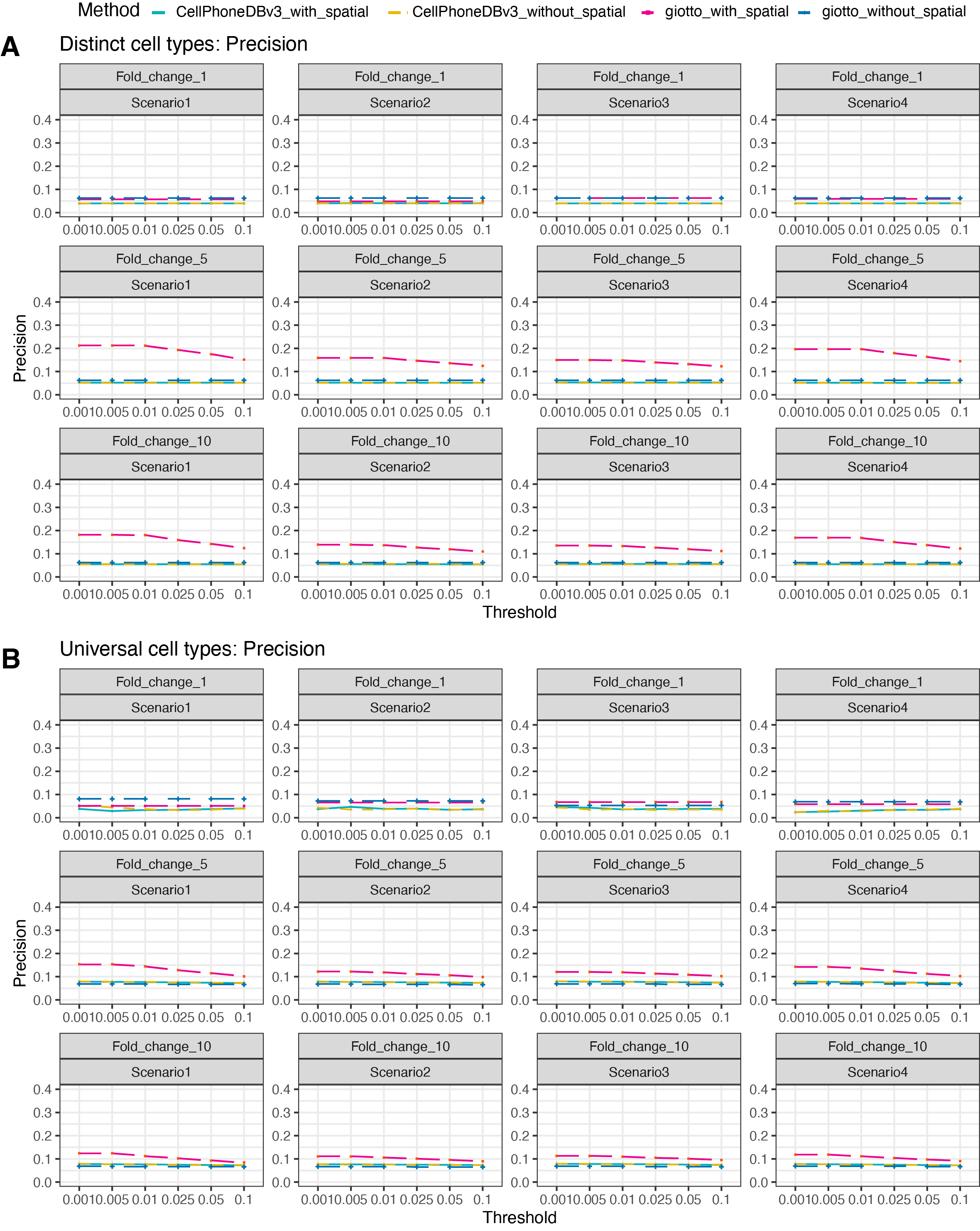
**

**Fig.S19 Benchmarking cell-cell communication identification methods using synthetic data from SRTsim (precision).** (**A**) The power of different methods for identifying cell-cell communications is measured by mean precision (y-axis) across a range of adjusted p-value thresholds in the homogeneous expression settings. Compared methods include CellphoneDBv3 with spatial information (green dash line), CellphoneDBv3 without spatial information (yellow dash line), Giotto with spatial information (pink dash line), and Giotto without spatial information (blue dash line). Results are shown for four different scenarios (columns) with different cell-cell communication strength (rows). (**B**) The power of different methods for identifying cell-cell communications is measured by mean F1 scores across a range of adjusted p-value thresholds in the heterogeneous expression settings. Results are again shown for four different scenarios (columns) with different cell-cell communication strength (rows). Each setting includes 10 simulation replicates.

**
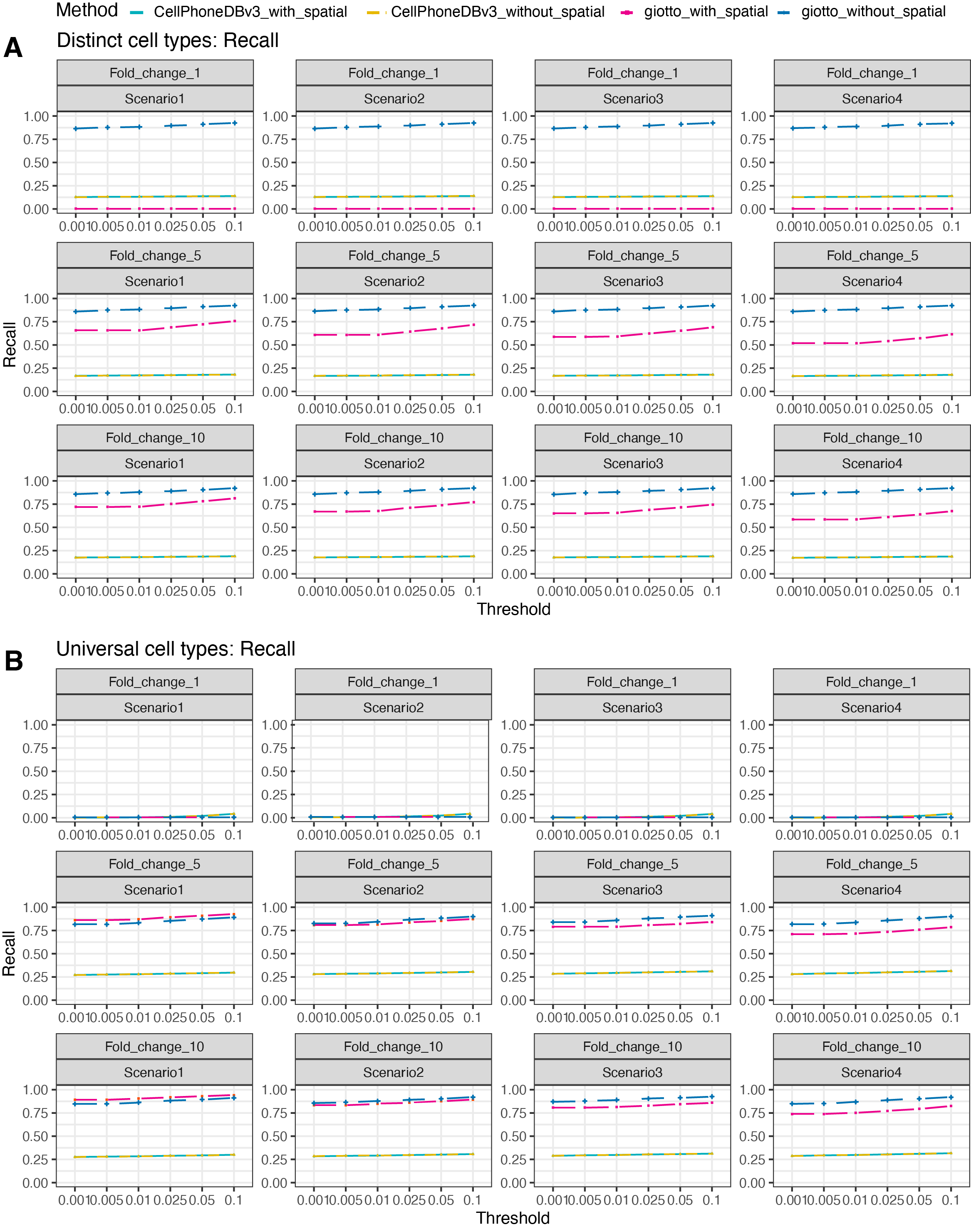
**

**Fig.S20 Benchmarking cell-cell communication identification methods using synthetic data from SRTsim (recall rate).** (**A**) The power of different methods for identifying cell-cell communications is measured by mean recall rate (y-axis) across a range of adjusted p-value thresholds in the homogeneous expression settings. Compared methods include CellphoneDBv3 with spatial information (green dash line), CellphoneDBv3 without spatial information (yellow dash line), Giotto with spatial information (pink dash line), and Giotto without spatial information (blue dash line). Results are shown for four different scenarios (columns) with different cell-cell communication strength (rows). (**B**) The power of different methods for identifying cell-cell communications is measured by mean F1 scores across a range of adjusted p-value thresholds in the heterogeneous expression settings. Results are again shown for four different scenarios (columns) with different cell-cell communication strength (rows). Each setting includes 10 simulation replicates.

**
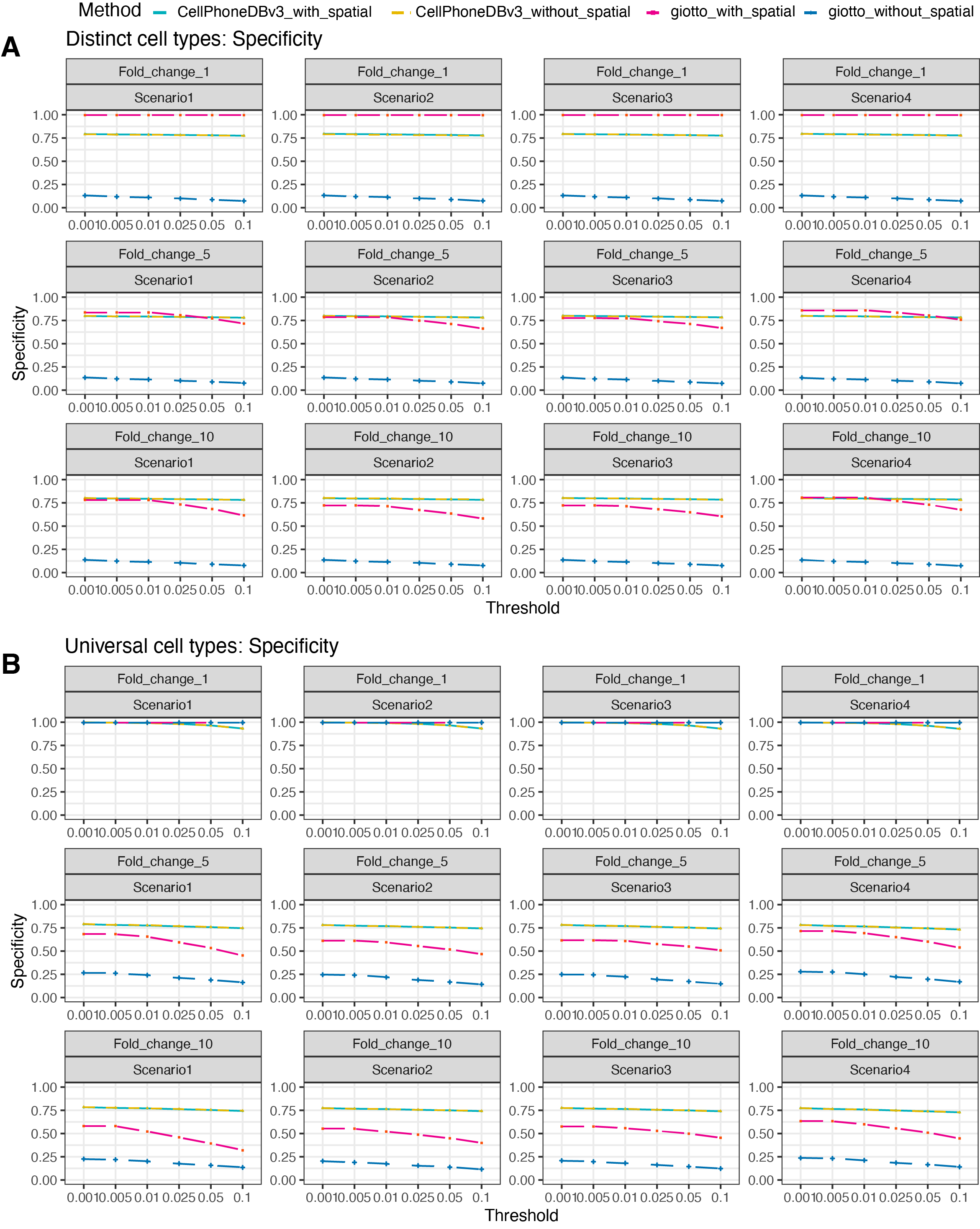
**

**Fig.S21 Benchmarking cell-cell communication identification methods using synthetic data from SRTsim (specificity).** (**A**) The power of different methods for identifying cell-cell communications is measured by mean specificity (y-axis) across a range of adjusted p-value thresholds in the homogeneous expression settings. Compared methods include CellphoneDBv3 with spatial information (green dash line), CellphoneDBv3 without spatial information (yellow dash line), Giotto with spatial information (pink dash line), and Giotto without spatial information (blue dash line). Results are shown for four different scenarios (columns) with different cell-cell communication strength (rows). (**B**) The power of different methods for identifying cell-cell communications is measured by mean F1 scores across a range of adjusted p-value thresholds in the heterogeneous expression settings. Results are again shown for four different scenarios (columns) with different cell-cell communication strength (rows). Each setting includes 10 simulation replicates.

**Table S1: Summary of 49 SRT datasets used in the present study.**

| **Sample Name** | **Number of Locations** | **Number of Genes** | **Technique** | **Resources** |
| --- | --- | --- | --- | --- |
| **V1_Adult_Mouse_Brain** | 2702 | 21949 | 10X Visium | <https://www.10xgenomics.com/resources/datasets> |
| **V1_Breast_Cancer_Block_A_Section_1** | 3798 | 24923 | 10X Visium |  |
| **V1_Breast_Cancer_Block_A_Section_2** | 3987 | 25072 | 10X Visium |  |
| **V1_Human_Heart** | 4247 | 20917 | 10X Visium |  |
| **V1_Human_Lymph_Node** | 4035 | 25187 | 10X Visium |  |
| **V1_Mouse_Brain_Sagittal_Anterior** | 2695 | 21334 | 10X Visium |  |
| **V1_Mouse_Brain_Sagittal_Anterior_Section_2** | 2825 | 21221 | 10X Visium |  |
| **V1_Mouse_Brain_Sagittal_Posterior** | 3355 | 21363 | 10X Visium |  |
| **V1_Mouse_Brain_Sagittal_Posterior_Section_2** | 3289 | 21174 | 10X Visium |  |
| **V1_Mouse_Kidney** | 1438 | 20100 | 10X Visium |  |
| **spatialLIBD_151507** | 4226 | 21151 | 10X Visium | <http://spatial.libd.org/spatialLIBD/> |
| **spatialLIBD_151508** | 4384 | 20737 | 10X Visium |  |
| **spatialLIBD_151509** | 4789 | 21279 | 10X Visium |  |
| **spatialLIBD_151510** | 4634 | 21089 | 10X Visium |  |
| **spatialLIBD_151669** | 3661 | 21235 | 10X Visium |  |
| **spatialLIBD_151670** | 3498 | 21019 | 10X Visium |  |
| **spatialLIBD_151671** | 4110 | 21631 | 10X Visium |  |
| **spatialLIBD_151672** | 4015 | 21343 | 10X Visium |  |
| **spatialLIBD_151673** | 3639 | 21842 | 10X Visium |  |
| **spatialLIBD_151674** | 3673 | 22437 | 10X Visium |  |
| **spatialLIBD_151675** | 3592 | 21344 | 10X Visium |  |
| **spatialLIBD_151676** | 3460 | 21312 | 10X Visium |  |
| **Breast Cancer Layer 2** | 251 | 14789 | ST | <https://www.spatialresearch.org/resources-published-datasets/doi-10-1126science-aaf2403/> |
| **MOB Replicate 11** | 262 | 16218 | ST |  |
| **GSM4096261_10t** | 2500 | 22969 | DBiT-seq | <https://www.ncbi.nlm.nih.gov/geo/query/acc.cgi?acc=GSE137986> |
| **GSM4096262_0725cL** | 936 | 22502 | DBiT-seq |  |
| **GSM4189611_50t** | 902 | 21832 | DBiT-seq |  |
| **GSM4189612_0628cL** | 1667 | 22445 | DBiT-seq |  |
| **GSM4189613_0702cL** | 901 | 22370 | DBiT-seq |  |
| **GSM4189614_0713cL** | 1789 | 18510 | DBiT-seq |  |
| **GSM4189615_0719cL** | 2500 | 20268 | DBiT-seq |  |
| **GSM4364242_E11-1L** | 1837 | 20751 | DBiT-seq |  |
| **GSM4364243_E11-2L** | 1662 | 21764 | DBiT-seq |  |
| **GSM4364244_E11-FL-1L** | 1840 | 21023 | DBiT-seq |  |
| **GSM4364245_E11-FL-2L** | 2109 | 20169 | DBiT-seq |  |
| **cortex_seqfish_plus** | 913 | 10000 | seqFISH+ | <https://github.com/CaiGroup/seqFISH-PLUS> |
| **ob_seqfish_plus** | 2050 | 10000 | seqFISH+ |  |
| **starmap_1020_0410** | 930 | 1020 | STARmap | <https://www.starmapresources.com/data> ^#^ |
| **Puck_190926_03** | 23124 | 51649 | SlideseqV2 | <https://singlecell.broadinstitute.org/single_cell/study/SCP815/highly-sensitive-spatial-transcriptomics-at-near-cellular-resolution-with-slide-seqv2> |
| **Lung5_Rep1_FOV1** | 2408 | 980 | CosMx SMI | <https://nanostring.com/products/cosmx-spatial-molecular-imager/ffpe-dataset/> |
| **Lung6_FOV1** | 2791 | 980 | CosMx SMI |  |
| **Lung9_Rep1_FOV1** | 5250 | 980 | CosMx SMI |  |
| **Lung12_FOV1** | 3241 | 980 | CosMx SMI |  |
| **Lung13_FOV1** | 3588 | 980 | CosMx SMI |  |
| **HumanBreastCancerPatient1_FOV659** | 536 | 550 | MERFISH* | <https://vizgen.com/data-release-program/> |
| **HumanOvarianCancerPatient1_FOV1051** | 635 | 550 | MERFISH |  |
| **HumanProstateCancerPatient1_FOV1013** | 588 | 550 | MERFISH |  |
| **Liver1Slice1_FOV1646** | 335 | 385 | MERFISH |  |
| **BrainReceptorShowcase_S1R1_FOV217** | 230 | 463 | MERFISH |  |

# We initially downloaded the data from starmapresources.org. However, the entire STARmap website is expired as of 11/13/2022. Therefore, we have uploaded the dataset to <https://xzhoulab.github.io/SRTsim/>.

* For MERFISH datasets, fields of view with maximum number of spatial locations were selected

**Table S5: Computational stability of all simulators.** X represents failure, while – represents success.

| **Sample Name** | **SRTsim** | **scDesign2** | **SymSim** | **SPARSim** | **ZINB-WaVE** | **Kersplat** | **Splat Simple** | **Splat** |
| --- | --- | --- | --- | --- | --- | --- | --- | --- |
| **V1_Adult_Mouse_Brain_10X** | - | - | - | - | - | - | - | - |
| **V1_Breast_Cancer_Block_A_Section_1_10X** | - | - | - | - | - | - | - | - |
| **V1_Breast_Cancer_Block_A_Section_2_10X** | - | - | - | - | - | - | - | - |
| **V1_Human_Heart_10X** | - | - | - | - | - | - | - | - |
| **V1_Human_Lymph_Node_10X** | - | - | - | X | - | - | - | - |
| **V1_Mouse_Brain_Sagittal_Anterior_10X** | - | - | - | - | - | - | - | - |
| **V1_Mouse_Brain_Sagittal_Anterior_Section_2_10X** | - | - | - | - | - | - | - | - |
| **V1_Mouse_Brain_Sagittal_Posterior_10X** | - | - | - | X | - | - | - | - |
| **V1_Mouse_Brain_Sagittal_Posterior_Section_2_10X** | - | - | - | - | - | - | - | - |
| **V1_Mouse_Kidney_10X** | - | - | - | X | - | - | - | - |
| **spatialLIBD_151507** | - | - | - | - | - | - | - | - |
| **spatialLIBD_151508** | - | - | - | - | - | - | - | - |
| **spatialLIBD_151509** | - | - | - | - | - | - | - | - |
| **spatialLIBD_151510** | - | - | - | X | - | - | - | - |
| **spatialLIBD_151669** | - | - | - | - | - | - | - | - |
| **spatialLIBD_151670** | - | - | - | - | - | - | - | - |
| **spatialLIBD_151671** | - | - | - | - | - | - | - | - |
| **spatialLIBD_151672** | - | - | - | - | - | - | - | - |
| **spatialLIBD_151673** | - | - | - | - | - | - | - | - |
| **spatialLIBD_151674** | - | - | - | - | - | - | - | - |
| **spatialLIBD_151675** | - | - | - | - | - | - | - | - |
| **spatialLIBD_151676** | - | - | - | - | - | - | - | - |
| **Layer2_BC_ST** | - | - | - | X | - | - | - | - |
| **Rep11_MOB_ST** | - | - | - | - | - | - | - | - |
| **GSM4096261_10t_DBit** | - | - | - | X | - | - | - | - |
| **GSM4096262_0725cL_DBit** | - | - | - | - | - | - | - | - |
| **GSM4189611_50t_DBit** | - | - | - | - | - | - | - | - |
| **GSM4189612_0628cL_DBit** | - | - | - | X | - | - | - | - |
| **GSM4189613_0702cL_DBit** | - | - | - | - | - | - | - | - |
| **GSM4189614_0713cL_DBit** | - | - | - | - | - | - | - | - |
| **GSM4189615_0719cL_DBit** | - | - | - | - | - | - | - | - |
| **GSM4364242_E11-1L_DBit** | - | - | - | - | - | - | - | - |
| **GSM4364243_E11-2L_DBit** | - | - | - | X | - | - | - | - |
| **GSM4364244_E11-FL-1L_DBit** | - | - | - | X | - | - | - | - |
| **GSM4364245_E11-FL-2L_DBit** | - | - | - | X | - | - | - | - |
| **middle_merfish** | - | - | - | - | - | - | - | - |
| **cortex_seqfish_plus** | - | - | - | X | - | X | - | - |
| **ob_seqfish_plus** | - | - | - | - | - | - | - | - |
| **starmap_1020_0410** | - | - | - | - | - | - | - | - |
| **Puck_190926_03_slideseqV2** | - | - | X | X | - | - | - | - |
| **Lung5_Rep1_FOV1_CosMx** | - | - | - | X | - | - | - | - |
| **Lung6_FOV1_CosMx** | - | - | - | X | - | - | - | - |
| **Lung9_Rep1_FOV1_CosMx** | - | - | - | X | - | X | - | - |
| **Lung12_FOV1_CosMx** | - | - | - | X | - | X | - | - |
| **Lung13_FOV1_CosMx** | - | - | - | X | - | - | - | - |
| **HumanBreastCancerPatient1_FOV659_MERFISH** | - | - | - | X | - | - | - | - |
| **HumanOvarianCancerPatient1_FOV1051_MERFISH** | - | - | - | - | - | - | - | - |
| **HumanProstateCancerPatient1_FOV1013_MERFISH** | - | - | - | X | - | - | - | - |
| **Liver1Slice1_FOV1646_MERFISH** | - | - | - | X | - | - | - | - |
| **BrainReceptorShowcase_S1R1_FOV217_MERFISH** | - | - | - | - | - | - | - | - |

**Table S6: Total number of alternatives in SE analysis.**

|  | **Scenario 1** | **Scenario 2** |
| --- | --- | --- |
| **Number of Patterns** | 7 | 4 |
| **SE Strength** | 5 or 10 | 3 or 4 |
| **Number of Alternatives Per Scenario** | 14 | 8 |
| **Total Number of Alternatives** | 22 | |
