# Supplementary material for: SRTsim: spatial pattern preserving simulations for spatially resolved transcriptomics
Source: Genome Biol. 2023 Mar 3;24:39. doi: 10.1186/s13059-023-02879-z (PMC9983268; doi:10.1186/s13059-023-02879-z)
Supplement: Supplementary file 5 — Additional file 5. Review history. [file 13059_2023_2879_MOESM5_ESM.docx]

Review History

**First round of review**

**Reviewer 1**

**Were you able to assess all statistics in the manuscript, including the appropriateness of statistical tests used?**

Yes: Proper use and presentation of statistics.

**Were you able to directly test the methods?**

Yes.

**Comments to author:**

In this manuscript, the authors presented the spatially resolved transcriptomics simulator (SRTsim) and claims that it offers scalable, reproducible, and realistic SRT simulations. The authors presented benchmarking studies for spatial clustering and spatial expression pattern detection comparing SRTsim with other simulation methods including ZINBWaVE, SPARSim, SymSim, two variants of scDesign2 (with/without copula), and three variants of Splat in the Splatter package (Splat Simple, Splat, and Kersplat). The authors used 40 SRT datasets from seven different SRT platforms that include 10x Visium, MERFISH, seqFISH+, STARmap, DBiT-seq, ST, and SlideseqV2. The proposed simulator is of interest to the spatially resolved transcriptomics research community and the authors presented relatively comprehensive benchmarking studies. The reviewer also commends the authors for releasing their code. However, the reviewer has the following concerns:

1. The authors missed some of the frequently used SRT techniques, such as GeoMX and CosMX. This casts concerns on whether the simulator will be biased towards certain techniques but not the others.

2. The authors presented two case studies on benchmarking spatial expression patterns and benchmarking spatial clustering methods. An equally important if not more important utility of SRT techniques is to study cell-cell interactions. The authors are recommended to include a benchmarking study on the investigation of cell-cell interactions to more convincingly demonstrate the utility of the proposed SRTsim method.

**Reviewer 2**

**Were you able to assess all statistics in the manuscript, including the appropriateness of statistical tests used?**

Yes: I assessed the statistical aspects of the paper - they were adequate.

**Were you able to directly test the methods?**

Yes.

**Comments to author:**

This manuscript presents a package for the realistic simulation of spatially resolved transcriptomics. In doing so, they show the ability of the simulated data to mimic the real data using 40 datasets and compared to 8 existing methods for scRNAseq. In particular, they focused on results from the DLPFC as a representative study. They compared properties of the real data to simulated data (gene-wise and location-wise metrics). They focused on the DLPFC study and the expression of three neuron layer-specific genes and found that the SRTsim outperformed the simulation of realistic data. They also compared the computational time for data simulation. In these comparisons, SRTsim outperformed the other simulation approaches used for scRNAseq. They then showed how SRTsim could be used for benchmarking methods (clustering and spatial expression analysis). Overall the paper is well written with extensive supplemental material/results. Below are my comments that arose when I read the paper.

Major:

1. The description and figure that gives the overview of how you preserve the spatial dependency in the tissue needs some more detail as it is not entirely clear how the spatial dependency in the data is preserved. Perhaps some more detail on the spot-ranking would help address this issue.

2. The authors tested SRTsim on a variety of tissues. Nevertheless, the DLPFC corresponds to a well-structured tissue with define layers. The versatility of SRTsim would be demonstrated if the authors also presented details on its performance in less structured tissues such as the cancer data sets they used. Is it possible that the spot ranking method would work well in structured tissues but not on un-structured ones?

3. Why is HMRF so different in the performance for the two datasets? Is there a reason that you might be able to provide?

4. Figure 4F. Why is distribution of ARI for spaGCN and spaGCNrefined so wide and bimodal compared to the other methods? Perhaps providing detail on which samples belong to each mode of the ARI distribution, can also yield useful insights on which tissue types methods work best.

5. Figure 5D and 5H; what is varying in the 4 panels of the FDRs? Maybe add more information to the caption.

6. In the results, it is mentioned that MERFISH and seqFISH data is not as well simulated as the other data types. Can the authors speculate a bit more why SRTsim cannot fully capture the properties of those techniques? Does SRTsim works best with only gridded spatial transcriptomics?

7. In the discussion, say that NB would be preferable model but you don't show the data from the modeling comparisons of ZIP, P, NB and ZINB. In the 40 studies, what model fit the best most of the time? Please present this data/information in the main part of the paper.

8. All the examples presented in the main paper seem to be based on the reference-based approach. Please consider putting some results based on non-reference simulation.

9. In the R package, it would be nice to have the information within the spatialExperiment object that is produced from the simulation (e.g., meta-data such as what gene model was used for each of the genes).

10. For the application 2 where get 22 alternative settings, with two scenarios how did you get 22 alternatives.

11. Adding to the main paper the results from a cancer sample; right now both examples are from brain tissue.

Minor:

1. Switch panel figure 3D with figure 3E-G as reference them in the paper in different order.

2. In application 2, line 53, I would not say that the methods failed to control type I error rate, rather one was conservative. I would say the failed to control type I error rate if the methods said that too many were significant (alpha was not 0.05 but something larger).

3. Would not say that GeoMX is microdissection. Page 1, line 14.

4. Page 1, line 32, please cite the studies that have done simulation of spatial transcriptomics. Li et al 2022 (Nat Met) and Liu et al 2022 (NAR).

5. Can the authors provide more detail on the options used to run each of the clustering methods for reproducibility? For example, the method SpaGCN features a number of options that could affect the outcome. The authors mention that the refined option of SpaGCN takes into account the pixel coordinates, but it's unclear whether the color intensity of the H&E image ( in the case of DLPFC) was used as an input. More details on the algorithm option would help clarify (and benchmark) these methods.

6. Data availability: The link to STARmap data appears to be broken (https://www.starmapresources.com/data). I did not check all the links.

**Authors Response**

**Point-by-point responses to the reviewers’ comments:**

Reviewer: 1#

General Comments:

In this manuscript, the authors presented the spatially resolved transcriptomics simulator (SRTsim) and claims that it offers scalable, reproducible, and realistic SRT simulations. The authors presented benchmarking studies for spatial clustering and spatial expression pattern detection comparing SRTsim with other simulation methods including ZINBWaVE, SPARSim, SymSim, two variants of scDesign2 (with/without copula), and three variants of Splat in the Splatter package (Splat Simple, Splat, and Kersplat). The authors used 40 SRT datasets from seven different SRT platforms that include 10x Visium, MERFISH, seqFISH+, STARmap, DBiT-seq, ST, and SlideseqV2. The proposed simulator is of interest to the spatially resolved transcriptomics research community and the authors presented relatively comprehensive benchmarking studies. The reviewer also commends the authors for releasing their code.

*Response: Thank you for the positive comments and constructive suggestions, which have substantially improved the quality of the manuscript. Our detailed responses to your specific comments are listed below.*

Specific Comments:

1. The authors missed some of the frequently used SRT techniques, such as GeoMX and CosMX. This casts concerns on whether the simulator will be biased towards certain techniques but not the others.

*Response: Thank you for the comment. Following your suggestion, we have carefully examined the properties of the data collected by the GeoMx Digital Spatial Profiler (DSP) and CosMx Spatial Molecular Imaging (SMI) techniques. GeoMx DSP is a commercial spatial transcriptomics platform that is primarily used to measure gene expression on a few targeted regions of interest (ROIs). The number of ROI assayed in each GeoMx DSP study is typically very small. For example, when we examined the few publicly available GeoMx DSP datasets/data sources [1-3] and the example data available from the GeomxTools package, we found that the median number of ROI per sample for a typical GeoMx DSP study is only 4 (mean=8.32; range = 2 – 22). In addition, each ROI in a GeoMx DSP study is relatively large (diameter ranges from 50-600 um [3]) and often contains a relatively large number of cells. Consequently, simulating GeoMx DSP data may be directly achieved by using standard bulk RNAseq simulators, potentially with some additional simple modifications to account for the general correlation structure among the few different locations. Because of the relatively small scale of GeoMx data and the potential applicability of bulk RNAseq simulators there, we did not explore GeoMx-based simulations further. Instead, we included a short discussion in line of above in the updated Discussion section (lines 384-392 on page 9).*

*CosMx SMI, on the other hand, relies on in situ hybridization (ISH) to detect RNA in intact biological samples at subcellular resolution. Through multiple cycles of hybridization, the CosMx SMI is capable of assaying up to 1,000 genes on 100k to 600k cells per tissue slide [4]. Because of the similarity between CosMx SMI and smFISH based spatial transcriptomics techniques (e.g. MERFISH), we expect SRTsim to work well with CosMx. To examine this, we obtained five datasets collected by CosMx SMI on five non-small-cell lung cancer (NSCLC) tissues (with one dataset per tissue). We then applied SRTsim along with the eight other simulators for reference-based simulations. In the analysis, we found that SRTsim could both capture various data properties well (Figs.3A and S5, Tables S3-4) and preserve the spatial expression pattern of genes well (Fig.3B). In contrast, scDesign2 fails to capture the location-wise properties. SymSim, kersplat, splat and splat simple fail to capture either gene-wise properties or location-wise properties. SPARsim was unable to generate synthetic data for this technique and produced software errors. We have included these new results to the updated Figure 3, with details described in the updated Results section (lines 114-146 on page 3).*

2. The authors presented two case studies on benchmarking spatial expression patterns and benchmarking spatial clustering methods. An equally important if not more important utility of SRT techniques is to study cell-cell interactions. The authors are recommended to include a benchmarking study on the investigation of cell-cell interactions to more convincingly demonstrate the utility of the proposed SRTsim method.

*Response: Good Point. Following your comments, we performed comprehensive and realistic simulations using SRTsim to examine its utility on benchmarking different methods for cell-cell communications. Specifically, we evaluated the performance of two cell-cell communication methods, Giotto and CellphoneDBv3, with two versions for each method. The simulation and analysis details are provided in the updated Methods section (lines 703-779 on pages 16-18) and are below.*

*Specifically, we used the single-cell resolution STARmap dataset to serve as the reference to generate gene expression on the tissue. To do so, we first estimated the shape profile based on the spatial locations of the reference data and randomly generated 5,000 locations within the estimated shape profile to serve as the single cell locations. We assumed that these single cells belong to four different cell types and we created four equal sized regions on the tissue with distinct cell type composition in each region. In particular, we set the cell types with the highest to lowest proportions to be cell type 1, 2, 3, and 4 in the first spatial region; to be 2, 3, 4, 1 in the second spatial region; to be 3, 4, 1, 2 in the third spatial region; and to be 4, 1, 2, 3 in the fourth spatial region. We then considered four cell type composition scenarios in the simulations. In the first scenario, each layer contains one dominant cell type, with 70% of the cells belonging to the dominant cell type and 10% of the cells belong to each of the three minor cell types. In the second scenario, each layer contains two dominant cell types with equal proportion, each consisting of 45% of the cells, along with two minor cell types each consisting of 5% of the cells. In the third scenario, each layer contains two major cell types with unequal proportion, with one consisting of 60% of the cells and the other consisting of 30% of the cells, along with two minor cell types each consisting of 5% of the cells. In the fourth scenario, each layer contains three major cell types and one minor cell type, consisting of 35%, 30%, 30%, or 5% of the cells, respectively. In each scenario, we randomly assigned each single cell in each region to one of the four cell types based on a multinomial distribution with parameters set to be the cell type composition in the region. We performed 10 simulation replicates for each simulation scenario.*

*In each simulation replicate, we first simulated the expression level of 5,000 genes for each single cell in two different ways. In the first way, we simulated homogeneous gene expression by generating expression level for each gene for a total of 8,000 cells from a common negative binomial distribution, with parameters inferred based on all cells in the reference data. We then randomly assigned these cells into four cell types, with 2,000 cells for each cell type. In the second way, we simulated heterogeneous gene expression by randomly selecting four cell types in the STARmap dataset and then generating the expression level of each gene in 2,000 cells for each cell type using a cell type specific negative binomial distribution, where the distribution parameters are estimated based on the particular cell type in the reference data. In either way, we randomly sampled cells from the 2,000 cells in each cell type and assigned them to the spatial locations according to the cell type composition described in the above paragraph.*

*Next, we increased the expression levels for the ligand-receptor pairs that mediate the cell-cell communications. To do so, we obtained 445 ligands and 471 receptors from CellphoneDBv3. These ligands and receptors correspond to 691 unique genes and form 930 ligand-receptor (L-R) gene pairs. For each L-R pair in turn, we randomly assign it to a pair of cell types (A-B), in order to create the scenario that cell type A communicate with cell type B through the ligand on cell type A and the receptor on cell type B. We denote such interaction as L-R-A-B pair. For four cell types, we obtained a total of 16 combinations of (ordered) cell type pairs, with the number of L-R pairs for each cell type pair ranging from 47 to 77 (median=57) in the simulations. With the allocated L-R-A-B pairs, we increased the gene expression for the L and R genes so that the L-R pair on adjacent A-B cell types are higher than that in the other cell types. In particular, for each cell in turn, we first identified its nearest four cells on the tissue and treat them as adjacent cells. If an A cell is adjacent to a B cell, we then increased the expression level of the L gene in the A cell by a fixed fold, with the fold increase set to be either 1 (null), 5, or 10, representing zero, moderate, or high level of cell-cell communications.*

*For each simulated spatial transcriptomics dataset, we evaluated the performance of four cell-cell communication methods to detect interacting L-R-A-B pairs on the tissue. These four methods include: (1) Giotto with spatial information; (2) Giotto without spatial information; (3) CellphoneDBv3 with spatial information; and (4) CellphoneDBv3 without spatial information. Each method examines one L-R-A-B pair at a time and tests whether the examined pair is significant. Specifically, for Giotto with spatial information, it calculates the mean of two values: the average ligand expression level of cells in cell type A that interact with cell type B; and the average receptor expression level of cells in cell type B that interact with cell type A. Afterwards, it calculates the proportion of the means in the permutations that are higher or lower than the actual mean. For Giotto without spatial information, it calculates the mean of two values: the average ligand expression level of all cells in cell type A; and the average receptor expression level of all cells in cell type B. Afterwards, it calculates the proportion of the means in the permutations that are higher or lower than the observed mean. For CellphoneDBv3 with spatial information, it calculates the mean of the average receptor expression level of cell type A and the average ligand expression level of the cell type B. Afterwards, it calculates the proportion of the means in the permutations that are as high or higher than the actual mean, considering only combinations of cell type pairs that co-exist in a spatial domain. For CellphoneDBv3 without spatial information, it calculates the mean of the average receptor expression level of cell type A and the average ligand expression level of the cell type B. Afterwards, it calculates the proportion of the means in the permutations that are as high or higher than the actual mean, considering all combinations of cell type pairs.*

*We evaluated the performances of different methods in detecting the L-R-A-B pairs based on the F1 score, which is the harmonic mean of precision and recall, at different adjusted p-value thresholds of 0.001, 0.005, 0.01, 0.025, 0.05, or 0.1 [5]. While we primarily focused on usingF1 score, which is the most commonly used metric in the literature of cell-cell communications [5-7], we also examined other metrics including precision (TP/TP+FP), recall (TP/TP+FN), and specificity (TN/TN+FP). These results are included in the Supplementary Figures.*

*Overall, we found that Giotto with spatial information achieves the best performance. Specifically, in the heterogeneous simulation settings, in the absence of interaction (fold change=1), Giotto with spatial information achieves the lowest F1 scores. For example, at a p value threshold of 0.05, the mean F1 score for Giotto with spatial information is 0.007, 0.006, 0.007, and 0 for the four scenarios, respectively. In contrast, Giotto without spatial information has the highest F1 score (mean=0.117, 0.117, 0.117 and 0.117 for the four scenarios, Fig. 7A). The CellphonDBv3 with spatial information (mean=0.085, 0.085, 0.086 and 0.085) and CellphoneDBv3 without spatial information (mean=0.086, 0.085, 0.086, and 0.084) have similar mean F1 score values. In the presence of moderate cell-cell communications, Giotto with spatial information achieves the highest F1 scores (mean=0.204, 0.188, 0.191, and 0.196 for four scenarios, Fig. 7A), demonstrating its power. Giotto without spatial information (mean=0.117, 0.117, 0.116, and 0.117) performs slightly better than CellphoneDBv3 with spatial information (mean=0.081, 0.08, 0.082, and 0.079) and without spatial information (mean=0.08, 0.081, 0.08 and 0.08, Fig. 7A). The two methods of CellphoneDBv3 perform similarly and are worse than the two Giotto methods, possibly because CellphoneDBv3 only incorporates regional spatial information without fully utilizing cell -cell spatial proximity as used in Giotto with spatial information. Finally, in the presence of high cell-cell communications, Giotto with spatial information still achieves the highest F1 score (Fig. 7A). The results are somewhat similar in the homogenous simulation settings (Fig. 7B).*

While Giotto with spatial information achieves the best performance in terms of F1 score, we note that all methods can be advantageous when measured with the other metrics. In particular, Giotto with spatial information achieves the highest precision (Figs. S21A-B); Giotto without spatial information achieves the highest recall (Figs. S19A-B); and the two CellphoneDBv3 methods generally achieve the highest specificity (Figs. S20A-B).

*We have included these simulation details in the Methods section (lines 703-779 on pages 16-17) and presented the new results in the Results section (lines 300-343 on pages 7-8).*

Reviewer: 2#

General Comments:

This manuscript presents a package for the realistic simulation of spatially resolved transcriptomics. In doing so, they show the ability of the simulated data to mimic the real data using 40 datasets and compared to 8 existing methods for scRNAseq. In particular, they focused on results from the DLPFC as a representative study. They compared properties of the real data to simulated data (gene-wise and location-wise metrics). They focused on the DLPFC study and the expression of three neuron layer-specific genes and found that the SRTsim outperformed the simulation of realistic data. They also compared the computational time for data simulation. In these comparisons, SRTsim outperformed the other simulation approaches used for scRNAseq. They then showed how SRTsim could be used for benchmarking methods (clustering and spatial expression analysis). Overall the paper is well written with extensive supplemental material/results.

*Response: Thank you for the positive comments and constructive suggestions, which have substantially improved the quality of the manuscript. Our detailed responses to your specific comments are listed below.*

Specific Comments:

1. The description and figure that gives the overview of how you preserve the spatial dependency in the tissue needs some more detail as it is not entirely clear how the spatial dependency in the data is preserved. Perhaps some more detail on the spot-ranking would help address this issue.

*Response: Thank you for the comment. Following your suggestion, we have modified Figure 1 to include additional information on how the spatial dependency is preserved. Specifically, we modified the visualization of step 3 in Figure 1 to better illustrate the expression ordering/ranking step, where we ordered the synthetic expression data for a gene across spots based on the rank order of the same gene across spots observed in the reference data. In the case where the locations in the synthetic data are identical to the locations in the reference data, such ranking is direct and one to one matching. In the case where the locations in the synthetic data (n1) are different to the locations in the reference data (n2), we designed a two-step spot ranking approach: for each location in the synthetic dataset, we identify its k-nearest neighbors in the reference data, sample randomly one expression value from the k-nearest neighbors to create an intermediate reference expression data vector with length of n1, and assigned the synthetic expression data based on the ranking observed in the intermediate reference data. In either case, the spot ranking step allows SRTsim to capture the gene-wise and location-wise properties and preserve the spatial expression patterns in the reference data.*

2. The authors tested SRTsim on a variety of tissues. Nevertheless, the DLPFC corresponds to a well-structured tissue with define layers. The versatility of SRTsim would be demonstrated if the authors also presented details on its performance in less structured tissues such as the cancer data sets they used. Is it possible that the spot ranking method would work well in structured tissues but not on un-structured ones?

*Response: Thank you for the comment. Following your suggestion, we have included additional analysis by applying SRTsim to five new datasets collected from non-small-cell lung cancer (NSCLC) tissues using the CosMx SMI technique. The results are similar to what were observed on the DLPFC data. In particular, SRTsim captured the data properties in the NSCLC data well (Fig.3A) and preserved the spatial expression pattern observed there (Fig. 3B). The new results are shown in the updated Figure 3, with details included in the Results section (lines 114-146 on pages 3-4).*

3. Why is HMRF so different in the performance for the two datasets? Is there a reason that you might be able to provide?

*Response: Thank you for the comment. The HMRF model implemented in smfishhmrf combines a Gaussian component that models the gene expression with a spatial component that encourages spatial domain similarity for adjacent cells. In the two datasets, HMRF performs poorly in the synthetic data generated from DLPFC but reasonably well in the synthetic data generated from STARmap data. We think that the HMRF performance difference in the two datasets is likely due to two reasons. First, the two data are of different sparsity: the DLPFC data has 93.4% zero elements in the gene expression matrix, and the STARmap data only has 69.5% zeros. Because HMRF assumes that gene expression within each spatial region follows a Gaussian distribution [8], the high sparsity in the gene expression could violate the Gaussian assumption and lead to poor performance in DLPFC. The second reason is that the DLPFC tissue structure is more complex than the STARmap tissue structure. Specifically, DLPFC data was collected in a large capture area (6.5x6.5mm) that contains 7 spatial domains, while STARmap data was collected in a much smaller capture area (11.78x11.78um) with only 4 spatial domains. Because HMRF relies on a Potts model to incorporate neighborhood similarity in the spatial domain assignment, it may perform well when the tissue structure is simple and small but less so when the tissue region is large and when the structure is complex. We have provided a short explanation in line of the above in the updated discussion section (lines 372-382 on page 9).*

4. Figure 4F. Why is distribution of ARI for SpaGCN and SpaGCNrefined so wide and bimodal compared to the other methods? Perhaps providing detail on which samples belong to each mode of the ARI distribution, can also yield useful insights on which tissue types methods work best.

*Response: Thank you for the comment. We carefully investigated the cause for the bimodal distribution pattern of ARI with SpaGCN. We found that, in five out of the ten synthetic datasets simulated by SRTsim, SpaGCN produced high ARI values, with an average value across ten replicates above 0.8 (highest=0.91). In the other five synthetic datasets, SpaGCN produced ARI values below 0.6 (lowest=0.39). The first five datasets are not distinguishable from the second five datasets in terms of the distribution of the six count metrics we use (Fig.S16A vs B). However, some of the marker genes in the second five datasets (Fig.S16E) appear to show slightly more similar expression between layers as compared to the first five datasets (Fig.S16D). For example, SNAP25 shows slightly more similar expression pattern between the out-layers in the second five datasets as compared to the first datasets. This observation suggests that SpaGCN could achieve high clustering performance when layers are very distinguished from each other but may suffer when the pattern between layers become less apparent (due to the stochasticity of simulations). We have briefly explained this in the updated Results section (lines 253-256 on page 6).*

5. Figure 5D and 5H; what is varying in the 4 panels of the FDRs? Maybe add more information to the caption.

*Response: Thank you for the comment. Each panel in 5D (now 6D) represents the power for detecting the corresponding pattern displayed in 5B (now 6B). Each panel in 5H (now 6H) represents the power for detecting the corresponding pattern displayed in 5F (now 6F). We have clarified these in the updated figure captions.*

6. In the results, it is mentioned that MERFISH and seqFISH data is not as well simulated as the other data types. Can the authors speculate a bit more why SRTsim cannot fully capture the properties of those techniques? Does SRTsim works best with only gridded spatial transcriptomics?

*Response: Thank you for the comment. Indeed, while SRTsim was able to capture all the other metrics, it failed to capture one particular metric -- the gene-wise coefficient of variation (CV) -- in the MERFISH and seqFISH+ datasets. For the MERFISH data, we realized that this data was collected using an early version of MEERFISH that was developed five years ago. Therefore, we decided to obtain five new datasets generated via Vizgen, which is the commercial platform of the MERFISH, and examined the performance of SRTsim on these datasets. These five datasets were collected from mouse brain, mouse liver, human breast cancer, human ovarian cancer, and human prostate cancer, respectively. In these five datasets, we found that all gene-wise and location-wise metrics from the synthetic data generated by SRTsim closely resemble that of the real data (Fig. S5 and Tables S1, S3, and S4), suggesting that SRTsim is able to capture the count properties of the MERFISH data. Therefore, we suspect that SRTsim had relatively poor CV performance in the previous MERFISH dataset because of the poor data quality of that particular data, likely due to the early version of MERFISH technology [10]. Following your comment, we have now updated all our results based on the five Vizgen datasets (Fig. S5 and Tables S1, S3 and S4).*

*For the seqFISH+ data, as we will also explain in the response to your comment #7, this technique appears to be very different from the other spatial transcriptomics. Specifically, among the four count models we examined, while NB model is the most preferred model for the majority of genes in all other spatial transcriptomics techniques, ZINB model is the most preferred model for the majority of the genes in seqFISH+. In addition, because we only examined four different count models, it is possible that even the ZINB model is insufficient to capture all the count properties of seqFISH+. Therefore, SRTsim, just like all other simulators, does not work very well in seqFISH+. We have included a short paragraph in the Discussion section to discuss the unique features of the seqFISH+ data (lines 367-370 on page 9).*

7. In the discussion, say that NB would be preferable model but you don't show the data from the modeling comparisons of ZIP, P, NB and ZINB. In the 40 studies, what model fit the best most of the time? Please present this data/information in the main part of the paper.

*Response: Thank you for the comment. Following your comment, we have summarized the proportions of the preferred model for all 49 datasets, including the newly added five CosMx cancer datasets, in Fig. 3C. In the analysis, we found that NB is the preferred model for all datasets, with the only exception of the seqFISH+ datasets. We have included these results in the updated main Figure 3, with details provided in the Results section (lines 129-132 on page 4). The observation is consistent with a recent study that NB is the preferred model across a range of spatial transcriptomics datasets [11].*

8. All the examples presented in the main paper seem to be based on the reference-based approach. Please consider putting some results based on non-reference simulation.

*Response: Thank you for the comment. We apologize for not making it clear previously the key difference between reference-based and reference-free simulations. We should have also emphasized earlier that the boundary between reference-based and reference-free simulations is actually not that crystal clear. For example, when we perform reference-free simulations, we would still want to use the count distributions with parameters estimated from a real data to simulate the gene expression, in order to keep the simulations realistic. Sometimes we may also want to use the tissue shape profile from a real data to serve as the backbone even when we perform reference-free simulations. Therefore, we feel that the key difference between reference-based and reference-free simulations is not whether we used a reference or not at all (because we always use a reference), but rather on whether we actively used the reference to keep the spatial expression pattern of the simulated genes consistent with that of the reference. Specifically, we feel that the key difference is that the gene expression from the reference-based simulations either preserves or builds upon the spatial expression pattern observed in the reference while the gene expression from the reference-free simulations displays de novo spatial expression patterns defined by the user. If we agree with this key difference, then the previous application of SRTsim for benchmarking SE analysis methods is in fact using reference-free simulations (lines 258-298, Fig.6). We have now explicitly stated this key difference in the method overview subsection (lines 87-92 on page 3) and have included an additional sentence in the second application in the Results section (lines 265-268 on page 6) to make this point clear. In addition, following your comment as well as the comment #2 from reviewer #1, we have included an additional application of SRTsim, which also uses a reference-free framework, for benchmarking methods on detecting cell-cell communications (lines 300-343, 703-779, Fig.7). The results for this new application are shown in the updated Results section (lines 300-343 on pages 6-7).*

9. In the R package, it would be nice to have the information within the spatialExperiment object that is produced from the simulation (e.g., meta-data such as what gene model was used for each of the genes).

*Response: Great suggestion. We have modified our simulator to include the additional meta-data information in the form of spatialExperiment object. Specifically, we added refrowData, refcolData, simrowData and simcolData slots in the SRTsim object to store gene-wise and location-wise information.*

10. For the application 2 where get 22 alternative settings, with two scenarios how did you get 22 alternatives.

*Response: Thank you for the comment. We apologize for not explaining this clearly in the previous version of the manuscript. We previously examined multiple simulation settings in each of the two scenarios. Specifically, in the first scenario, we examined seven different spatial expression patterns (Figs. 6B and S16A). For each pattern, we examined two SE strength levels: 5-fold (Figs. 6D and S16B) and 10-fold (Fig. S16C). Therefore, we have a total of 14 alternative settings in scenario 1. Similarly, in the second scenario, we examined four different spatial expression patterns (Fig. 6F). For each pattern, we examined two SE strength levels: 3-fold (Fig.6H) and 4-fold (Fig.S17B). Therefore, we have a total of 8 alternative settings in scenario 2. In conclusion, we have 22 alternatives across the two scenarios. These details were previously provided in the Methods section (now lines 665-689 on pages 15-16). Following your comment, we have included one additional sentence in the Results section (lines 278-279 on page 6) to point readers to these details to the corresponding Methods section. We have also created a new table (Table S6) to explicitly list the 22 alternative settings across the two scenarios.*

11. Adding to the main paper the results from a cancer sample; right now both examples are from brain tissue.

*Response: Thank you for the comment. We have included five cancer datasets collected via CosMx SMI technique, and added one of them in the main paper. Please refer to our response to your comment #2 above.*

Minor comments:

1. Switch panel figure 3D with figure 3E-G as reference them in the paper in different order.

*Response: Thank you. We have switched the panel order as suggested.*

2. In application 2, line 53, I would not say that the methods failed to control type I error rate, rather one was conservative. I would say the failed to control type I error rate if the methods said that too many were significant (alpha was not 0.05 but something larger).

*Response: Thank you. We have modified the text to read “As SpatialDE produced overly conservative P values under null …” (line 284 on page 7).*

3. Would not say that GeoMX is microdissection. Page 1, line 14.

*Response: Thank you. We have removed GeoMX from this category.*

4. Page 1, line 32, please cite the studies that have done simulation of spatial transcriptomics. Li et al 2022 (Nat Met) and Liu et al 2022 (NAR).

*Response: Thank you. We have cited both studies.*

5. Can the authors provide more detail on the options used to run each of the clustering methods for reproducibility? For example, the method SpaGCN features a number of options that could affect the outcome. The authors mention that the refined option of SpaGCN takes into account the pixel coordinates, but it's unclear whether the color intensity of the H&E image ( in the case of DLPFC) was used as an input. More details on the algorithm option would help clarify (and benchmark) these methods.

*Response: Thank you. To ensure reproducibility, we have provided additional model fitting details in the Methods section (lines 643-661 on page 15) and have hosted all analysis code used in the present study on github (https://xzhoulab.github.io/SRTsim/). For your specific question on the refined option of SpaGCN, we didn’t use the color intensity of the H&E image for DLPFC but included two sets of spatial coordinates including both array coordinates and pixel coordinates. We used pixel coordinates in the regular version of SpaGCN. These details are now available in the aforementioned Methods section. We note that while stLearn and SpaGCN can make use of histology information, including histology image/color intensity in this particular dataset does not increase their performance, as histology image for this data does not contain clear spatial domain segmentation information.*

6. Data availability: The link to STARmap data appears to be broken (https://www.starmapresources.com/data). I did not check all the links.

*Response: Thank you. We downloaded the STARmap data from this link. However, the STARmap team changed the domain name to https://www.starmapresources.org/ early this year. As of 10/20/2022, the new domain name worked. As of 11/13/2022, the link expires again. So, we have uploaded this dataset to (https://xzhoulab.github.io/SRTsim/) and updated the link in the text accordingly (line 789 on page 18). We have carefully examined all the other links.*

*References:*

*1. Park J, Foox J, Hether T, Danko DC, Warren S, Kim Y, et al. System-wide transcriptome damage and tissue identity loss in COVID-19 patients. Cell Reports Medicine. 2022;3(2):100522.*

*2. Yerly L, Pich-Bavastro C, Di Domizio J, Wyss T, Tissot-Renaud S, Cangkrama M, et al. Integrated multi-omics reveals cellular and molecular interactions governing the invasive niche of basal cell carcinoma. Nature communications. 2022;13(1):1-16.*

*3. Pujadas E, Beaumont M, Shah H, Schrode N, Francoeur N, Shroff S, et al. Molecular profiling of coronavirus disease 2019 (COVID-19) autopsies uncovers novel disease mechanisms. The American Journal of Pathology. 2021;191(12):2064-71.*

*4. He S, Bhatt R, Brown C, Brown EA, Buhr DL, Chantranuvatana K, et al. High-plex imaging of RNA and proteins at subcellular resolution in fixed tissue by spatial molecular imaging. Nature Biotechnology. 2022:1-13.*

*5. Liu Q, Hsu CY, Li J, Shyr Y. Dysregulated ligand-receptor interactions from single-cell transcriptomics. Bioinformatics. 2022;38(12):3216-21. Epub 2022/04/29. doi: 10.1093/bioinformatics/btac294. PubMed PMID: 35482476; PubMed Central PMCID: PMCPMC9191214.*

*6. Wang DD, Ou-Yang L, Xie H, Zhu M, Yan H. Predicting the impacts of mutations on protein-ligand binding affinity based on molecular dynamics simulations and machine learning methods. Comput Struct Biotechnol J. 2020;18:439-54. Epub 2020/03/11. doi: 10.1016/j.csbj.2020.02.007. PubMed PMID: 32153730; PubMed Central PMCID: PMCPMC7052406.*

*7. Wang S, Zheng H, Choi JS, Lee JK, Li X, Hu H. A systematic evaluation of the computational tools for ligand-receptor-based cell-cell interaction inference. Brief Funct Genomics. 2022;21(5):339-56. Epub 2022/07/14. doi: 10.1093/bfgp/elac019. PubMed PMID: 35822343; PubMed Central PMCID: PMCPMC9479691.*

*8. Teng H, Yuan Y, Bar-Joseph Z. Cell type assignments for spatial transcriptomics data. bioRxiv. 2021.*

*9. Walker BL, Cang Z, Ren H, Bourgain-Chang E, Nie Q. Deciphering tissue structure and function using spatial transcriptomics. Communications biology. 2022;5(1):1-10.*

*10. Moffitt JR, Hao J, Wang G, Chen KH, Babcock HP, Zhuang X. High-throughput single-cell gene-expression profiling with multiplexed error-robust fluorescence in situ hybridization. Proceedings of the National Academy of Sciences. 2016;113(39):11046-51.*

*11. Zhao P, Zhu J, Ma Y, Zhou X. Modeling zero inflation is not necessary for spatial transcriptomics. Genome Biology. 2022;23(1):1-19.*

*12. Hu J, Li X, Coleman K, Schroeder A, Ma N, Irwin DJ, et al. SpaGCN: Integrating gene expression, spatial location and histology to identify spatial domains and spatially variable genes by graph convolutional network. Nature Methods. 2021;18(11):1342-51. doi: 10.1038/s41592-021-01255-8.*

**Second round of review**

**Reviewer 1**

The authors have answered well all the questions previously raised by this reviewer.

**Reviewer 2**

Thank you for your responses and the revised manuscript. Based on the number of changes/additions to the manuscript, the resulting manuscript is much improved.
